# Ramucirumab, Avelumab, and Paclitaxel as Second-Line Treatment in Esophagogastric Adenocarcinoma: The Phase 2 RAP (AIO-STO-0218) Nonrandomized Controlled Trial

**DOI:** 10.1001/jamanetworkopen.2023.52830

**Published:** 2024-01-23

**Authors:** Peter Thuss-Patience, Anica Högner, Eray Goekkurt, Michael Stahl, Albrecht Kretzschmar, Thorsten Götze, Gertraud Stocker, Peter Reichardt, Frank Kullmann, Daniel Pink, Prisca Bartels, Armin Jarosch, Axel Hinke, Christoph Schultheiß, Lisa Paschold, Alexander Stein, Mascha Binder

**Affiliations:** 1 Department of Hematology, Oncology and Cancer Immunology, Charité-University Medicine Berlin, Berlin, Germany; 2 Hematology-Oncology Practice Eppendorf, Hamburg, Germany; 3 Department of Medical Oncology, Evang. Kliniken Essen-Mitte, Essen, Germany; 4 Hematology-Oncology Practice Medizinisches Versorgungszentrum Mitte, Leipzig, Germany; 5 Institute of Clinical Cancer Research at Krankenhaus Nordwest, University Cancer Center, Frankfurt, Germany; 6 Leipzig University Cancer Center, Leipzig University Hospital, Leipzig, Germany; 7 Sarcoma Center Berlin-Brandenburg, Helios Klinikum Berlin-Buch, Berlin, Germany; 8 Department of Medicine I, Hospital Weiden, Weiden, Germany; 9 Department of Oncology and Palliative Care, Helios Klinikum Bad Saarow, Germany; 10 Department of Internal Medicine C, University Hospital Greifswald, Germany; 11 Laboratory of Molecular Tumor Pathology and Systems Biology, Institute of Pathology, Charité-University Medicine Berlin, Berlin, Germany; 12 Clinical Cancer Research Consulting, Düsseldorf, Germany; 13 Department of Internal Medicine IV, University Hospital Halle, Martin-Luther University Halle-Wittenberg, Germany; 14 University Cancer Center Hamburg, University Medical Center Hamburg-Eppendorf, Hamburg, Germany

## Abstract

**Importance:**

Adding immune checkpoint inhibitors to chemotherapy has been associated with improved outcomes in metastatic esophagogastric adenocarcinoma, but treatment combinations and optimal patient selection need to be established.

**Objective:**

To investigate the efficacy and tolerability of the programmed cell death ligand 1 (PDL-1) inhibitor avelumab with paclitaxel plus ramucirumab.

**Design, Setting, and Participants:**

This multicenter, single-group, phase 2 nonrandomized controlled trial was conducted among patients with second-line metastatic esophagogastric adenocarcinoma. Patients pretreated with platinum plus fluoropyrimidine between April 2019 and November 2020 across 10 German centers (median follow-up, 27.4 months [95% CI 22.0-32.9 months]) were included. Data analysis was performed from January to December 2022.

**Interventions:**

Patients received ramucirumab at 8 mg/kg on days 1 and 15, avelumab at 10 mg/kg on days 1 and 15, and paclitaxel at 80 mg/m^2^ on days 1, 8, and 15 every 4 weeks.

**Main Outcomes and Measures:**

The prespecified primary end point was overall survival (OS) rate at 6 months, with the experimental therapy considered insufficiently active with an OS rate of 50% or less and a promising candidate with an OS rate of 65% or greater.

**Results:**

Of 60 enrolled patients, 59 patients (median [range] age, 64 [18-81] years; 47 males [70.7%]) were evaluable, including 30 patients with metastatic adenocarcinoma of the stomach and 29 patients with gastroesophageal junction. All patients were pretreated with platinum plus fluoropyrimidine, and 40 patients (67.8%) had received prior taxanes; 24 of 56 evaluable patients (42.9%) had a PDL-1 combined positive score (CPS) of 5 or greater, centrally assessed. The OS rate at 6 months was 71.2% (95% CI, 61.5%-83.7%). The median OS in the intention-to-treat population (59 patients) was 10.6 months (95% CI, 8.4-12.8 months) overall. Among patients assessable by central pathology, median OS was 9.4 months (95% CI, 7.2-11.7 months) in 32 patients with a PDL-1 CPS less than 5 and 14.0 months (95% CI, 6.0-22.1 months) in 24 patients with a PDL-1 CPS of 5 or greater (*P* = .25). Treatment was generally well tolerated, without unexpected toxicities. Patients with higher vs lower than median T cell repertoire richness showed an increased median OS of 20.4 months (95% CI, 7.7-33.0 months) compared with 8.3 months (95% CI, 3.7-12.9 months; hazard ratio, 0.43; 95% CI, 0.23-0.81; *P* = .008). Patients with lower vs higher than median cell-free DNA burden had a median OS of 19.2 months (95% CI, 8.9-29.6 months) compared with 7.3 months (95% CI, 3.2-11.4 months; hazard ratio, 0.30; 95% CI, 0.16-0.59; *P* < .001).

**Conclusions and relevance:**

In this study, the combination of avelumab with paclitaxel plus ramucirumab showed favorable efficacy and tolerability in the second-line treatment for metastatic esophagogastric adenocarcinoma. A PDL-1 CPS score of 5 or greater, cell-free DNA level less than the median, and T cell repertoire richness greater than the median were associated with increased median OS.

**Trial Registration:**

ClinicalTrials.gov Identifier: NCT03966118

## Key Points

**Question:**

Is the combination of avelumab with paclitaxel plus ramucirumab effective and possibly synergistic in the second-line treatment for metastatic esophagogastric adenocarcinoma?

**Findings:**

In this nonrandomized controlled trial of 59 patients who had progressed on platinum plus fluoropyrimidine, overall survival rate at 6 months was 71.2%, with a median of 10.6 months overall and 14.0 months in those with a programmed cell death ligand 1 combined positive score of 5 or greater. A statistically significant survival benefit was seen in patients with low cell-free DNA levels (19.2 months) or high T cell repertoire richness (20.4 months).

**Meaning:**

In this study, subgroups with superior survival were identified, suggesting that combining avelumab with paclitaxel plus ramucirumab is effective.

## Teaser

This nonrandomized controlled trial investigates efficacy and tolerability of the combination of avelumab with paclitaxel plus ramucirumab in the treatment of metastatic esophagogastric adenocarcinoma.

## Introduction

Gastric cancer is a global problem affecting more than 1 million people worldwide per year.^1^ Most patients are diagnosed with metastatic disease and are amenable only to palliative systemic therapy. First-line systemic treatment is usually based on a platinum plus fluoropyrimidine backbone and, for patients with human epidermal growth factor receptor 2 (HER2)–overexpressing tumors, trastuzumab. More recently, programmed cell death 1 inhibitors combined with chemotherapy have become standard of care for programmed cell death ligand-1 (PDL-1)–overexpressing tumors.^2^ In the Checkmate 649 study,^3^ adding nivolumab to FOLFOX chemotherapy improved overall survival (OS), especially in patients with a PDL-1 combined positive score (CPS) of 5 or greater. Second-line treatment was associated with improved OS in esophagogastric adenocarcinoma (EGA).^4,5^ The most common treatment protocol combines the vascular endothelial growth factor receptor-2 (VEGFR-2) inhibitor ramucirumab with paclitaxel.^6^

It is unclear whether the benefit of a standard second-line treatment can be improved by combination with a checkpoint inhibitor. There is emerging preclinical evidence that simultaneous blockade of VEGFR-2 and immune checkpoints may be associated with enhanced T cell migration and antitumor activity.^7,8,9,10^ Clinically, some synergy between checkpoint inhibition (CPI) and ramucirumab has been suspected in gastric cancer.^11^ A trial in Japan^12^ showed promising results combining CPI, antiangiogenesis, and chemotherapy with paclitaxel. In the Ramucirumab, Avelumab and Paclitaxel (RAP) trial reported in this study, we tested the efficacy and tolerability of ramucirumab and paclitaxel combined with the checkpoint inhibitor avelumab (PDL-1 inhibitor) in the second-line treatment of patients with EGA. Due to the study’s single-group design, all results have to be considered as descriptive. This trial included longitudinal liquid biopsy monitoring to identify patients with prolonged survival.

## Methods

We performed a single-group, multicenter, phase 2 nonrandomized controlled trial, which was approved by ethics committees of participating centers (eTable 1 in Supplement 1) and complied with Good Clinical Practice guidelines and the Declaration of Helsinki. This study is reported following the Transparent Reporting of Evaluations With Nonrandomized Designs (TREND) reporting guideline. All patients signed informed consent. The trial was registered at ClinicalTrials.gov (NCT03966118) and European Union Drug Regulating Authorities Clinical Trials (EudraCT; 2018-002938-20).

### Patient Eligibility

Patients were aged 18 years or older with histologically confirmed adenocarcinoma of the stomach or gastroesophageal junction, an Eastern Cooperative Oncology Group performance status of 0 to 1, metastatic or locally advanced disease, status as incurable by operation, and documented objective radiological or clinical disease progression during or within 6 months of the last dose of first-line platinum and fluoropyrimidine doublet with or without anthracycline, docetaxel, or trastuzumab. Patients with previous treatment with checkpoint inhibitors were excluded. Measurable or nonmeasurable but evaluable disease according to Response Evaluation Criteria in Solid Tumors (RECIST) version 1.1 was mandatory.

### Measures of Outcome

The primary end point was the OS rate at 6 months. Secondary end points were Kaplan-Meier estimated OS and OS rate at 12 months, progression-free survival (PFS), response rate, duration of response, safety and tolerability according to the National Cancer Institute Common Terminology Criteria for Adverse Events version 5.0, and translational research end points, including cell-free DNA (cfDNA) levels and peripheral blood T cell receptor repertoire richness (TRB).

### Assessment of Toxic Effects, Safety, and Efficacy

RECIST version 1.1 assessment (including chest, abdomen, and pelvis) was done within 4 weeks prior to treatment start and every 8 weeks afterward. Clinical and laboratory assessment had to be done at baseline and day 1, 8, and 15 of each cycle. All patients had to be followed up for 2 years after the start of treatment (see the trial protocol in Supplement 2.).

To ensure data quality and safety, a data safety monitoring committee was informed on a regular basis. Pathology specimens were centrally reassessed for HER2, Epstein-Barr virus, microsatellite instability status, and PDL-1 expression level (CPS). Radiological images were reviewed by a central independent radiologist.

### Treatment

Patients received avelumab (10 mg/kg) by intravenous infusion over 60 to 90 minutes on day 1 and 15 of a 28-day cycle, ramucirumab (8 mg/kg) intravenously over 60 minutes on day 1 and 15, and paclitaxel (80 mg/m^2^) intravenously over 60 minutes on day 1, 8, and 15. The maximum treatment duration was 1 year with 1 year of follow-up thereafter.

### Translational Research

The most recent paraffin-embedded archival tissue was analyzed by central pathology (Charité–University Medicine Berlin) for HER2, microsatellite instability status, Epstein-Barr virus, and PDL-1 CPS. PD-L1 staining using E1L3N antibody (Cell Signaling Technology) was performed in a Leica BOND-MAX fully automated immunohistochemistry and in situ hybridization staining system (Leica Biosystems) following manufacturer instructions. Pretreatment tumor slides and peripheral blood samples obtained at baseline were further analyzed for tumor mutations, cfDNA levels, and TRB metrics. (eTable 2 in Supplement 1). Peripheral blood was collected in cfDNA blood-collection tubes (Streck). Plasma was obtained after centrifugation and taken for isolation of cfDNA. A set of selected gene regions covering the most frequent mutation hot spots in gastric cancer (eTable 3 in Supplement 1) was amplified from 100 ng cfDNA or genomic DNA via targeted next-generation sequencing. For TRB immunosequencing, genomic DNA was isolated from blood cell pellets from 250 ng of input leukocyte DNA.^13,14^ TRB richness was defined as the total number of clones in a distinct blood sample.

### Statistical Analysis

In this single-group, phase 2 trial with the primary end point of OS rate at 6 months, efficacy assumptions were derived from historical data. Paclitaxel and ramucirumab achieved an OS rate at 6 months of 66% in the Western population of the Rainbow trial.^15^ We expected worse prognostic parameters in our study population, with at least 50% of patients already pretreated with taxanes, a higher rate of tumors located in the gastroesophageal junction, and the overall broader selection criteria in an investigator-initiated study; thus we targeted an OS rate of 65% at 6 months by combining paclitaxel plus ramucirumab with avelumab. The experimental therapy would be considered a promising candidate for further development if the true OS rate amounted to 65% or more (type I error, 0.10) and insufficiently active if the true OS rate was 50% or lower (type II error, 0.20). A standard 2-stage phase II design was applied,^16^ which amounted to 53 evaluable patients (59 patients including dropouts).

Univariable and multivariable prognostic analyses were performed using Cox models. Parameters with a univariable *P* < .10 were included in the multivariable model, to be reduced by a stepwise backward procedure at a threshold of *P* < .10; all *P* values are 2-sided unless stated otherwise. Statistical analyses were performed using SPSS statistical software version 27.0 (IBM) and R statistical software version 3.0.2 (R Project for Statistical Computing) (see statistical analysis plan in Supplement 2). Data analysis was performed from January to December 2022.

## Results

### Main Analysis

**Figure 1.**
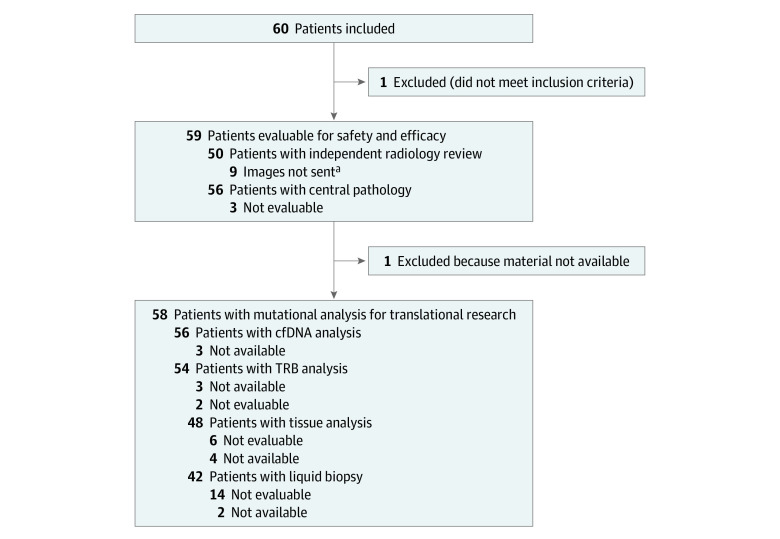
Study Flowchart Patients or samples excluded at different parts of the analysis are visualized. cfDNA indicates cell-free DNA; TRB, T cell receptor repertoire richness. ^a^Compact discs with computed tomography scan images were not sent for central review.

Among 60 enrolled patients between April 2019 and November 2020, 59 patients (median [range] age, 64 [18-81] years; 47 males [70.7%]) were evaluable, including 30 patients with metastatic adenocarcinoma of the stomach and 29 patients with gastroesophageal junction (Figure 1). All patients were pretreated with platinum and fluoropyrimidine, and 40 patients (67.8%) were additionally pretreated with a taxane. There was 1 patient who was not investigated due to violation of inclusion criteria. Median follow-up was 27.4 months (95% CI 22.0-32.9 months), and there was no loss of follow-up in our patient cohort. Among 56 evaluable patients, 24 patients (42.9%) had a PDL-1 CPS of 5 or greater at central pathology (Table 1).

**Table 1.** Baseline Characteristics

Characteristic	Patients, No. (%) (N = 59)
Age, median (range), y	64 (18-81)
Sex	
Male	47 (79.7)
Female	12 (20.3)
ECOG performance status	
0	23 (39.0)
1	36 (61.0)
Primary tumor site	
Gastric	30 (51.8)
GEJ	29 (48.2)
Histology (Lauren classification)	
Intestinal	34 (57.6)
Diffuse	14 (23.7)
Mixed	9 (15.3)
Not available	2 (3.4)
Signet ring cells	
No	46 (78.0)
Yes	10 (16.9)
Not available	3 (5.1)
Metastatic spread	
Metastatic	59 (100)
Locally advanced	0
Metastatic disease site	
Liver	25 (42.4)
Lung	10 (16.9)
Bone	3 (5.1)
Peritoneum or ascites	10 (16.9)
Pleura or pleural effusion	1 (1.7)
Lymph node	18 (30.5)
Central pathology	
HER2 status	
Positive	6 (10.2)
Negative	50 (84.7)
Not available	3 (5.1)
PDL-1 CPS	
0	23 (39.0)
1-4	9 (15.3)
5-9	8 (13.6)
≥10	16 (27.1)
Not available	3 (5.1)
MSI (IHC) status	
Stable	52 (88.1)
Unstable	4 (6.8)
Not available	3 (5.1)
Previous therapy	
Taxanes	
Yes	40 (67.8)
No	19 (32.2)
Platin plus FU	
Yes	59 (100)
Surgery for gastric cancer	
Yes	32 (54.2)
No	27 (45.8)
Discontinuation of last treatment prior to study	
Administered as planned	10 (16.9)
PD	46 (78.0)
Toxic effects	2 (3.4)
Patient decision	1 (1.7)

Abbreviations: CPS, combined positive score; ECOG, Eastern Cooperative Oncology Group; FU, fluorouracil; GEJ, gastroesophageal junction; HER2, human epidermal growth factor receptor 2; IHC, immunohistochemistry; MSI, microsatellite instability; PD, progressive disease; PDL-1, programmed cell death ligand 1.

Treatment was well tolerated, with no unexpected toxic effects observed. The most common grade 3 or 4 toxic effects were leukopenia or neutropenia in up to 23.7% of patients (grade 3: 11 patients [18.6%]; grade 4: 3 patients [5.1 %]), but neutropenic infections were rare. Grade 3 or 4 nonneutropenic systemic infections occurred in 7 patients (11.9%), including grade 3 in 6 patients (10.2%) and grade 4 in 1 patient (1.7%); grade 2 infections occurred in 5 patients (8.5%). The most frequently reported immune-related adverse events were rash (14 patients [23.8%], including grade 1 in 11 patients [18.7%] and grade 2 in 3 patients [5.1%]) and hypothyroidism (9 patients [15.3%], including grade 1 in 5 patients [8.5%] and grade 2 in 4 patients [6.8%]), but these were generally mild (eTable 4 in Supplement 1). There was 1 death due to esophagotracheal fistula, which may have been exacerbated by ramucirumab. In 30 patients (50.5%), there were a total of 51 serious adverse events reported. Of these, 8 events were probably associated with 1 or more treatment component and 1 event was possibly associated with avelumab.

In total, 360 cycles of therapy were initiated, with a cycle defined as days 1, 8, and 15 every 28 days. The median (range) number of cycles was 6 (1-17) cycles. Of 360 cycles, 94 cycles were started without paclitaxel, 31 cycles without ramucirumab, and 15 cycles without avelumab. Paclitaxel was discontinued in 34 patients, while ramucirumab was discontinued in 16 patients and avelumab was discontinued in 11 patients. A total 261 cycles (median [range], 4 [1-10] cycles) were started with all 3 drugs.

Efficacy as assessed by the local investigator as best response in 59 patients showed a complete response in 2 patients (3.4%), a partial response in 16 patients (27.1%), stable disease in 29 patients (49.2%), and progressive disease in 12 patients (20.3%). This gave an overall response rate of 18 patients (30.5%) and a disease control rate (complete response + partial response + stable disease) of 47 patients (79.7%). The median duration of response was 8.2 months (95% CI, 6.7-9.7 months) (Table 2).

**Table 2.** Efficacy^a^

Response assessed by investigator	Patients, No.(%) (N = 59)
CR	2 (3.4)
PR	16 (27.1)
SD	29 (49.2)
PD	12 (20.3)
DCR (CR + PR + SD)	47 (79.9)
ORR (CR + PR)	18 (30.5)
DOR, median (95% CI), mo	8.2 (6.7-9.7)

Abbreviations: CR, complete response; DCR, disease control rate; DOR, duration of response; ORR, overall response rate; PD, progressive disease; PR, partial response; SD, stable disease.

^a^ Efficacy was evaluated according to Response Evaluation Criteria in Solid Tumors (RECIST) version 1.1 by investigator assessment.

**Figure 2.**
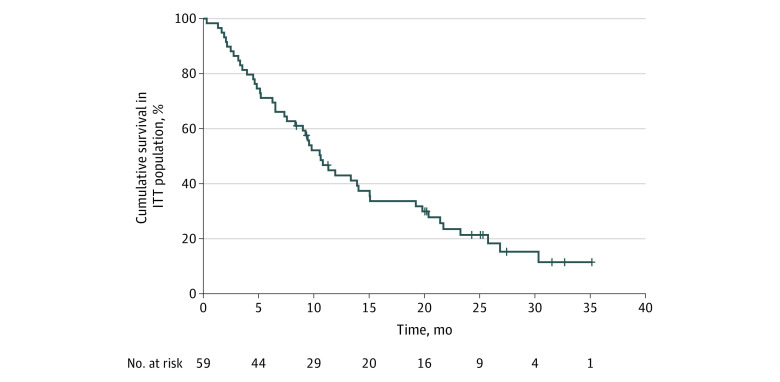
Overall Survival Rate in Intention-to-Treat (ITT) Population

The primary end point of the trial was met, with a 6-month OS of 71.2% (95% CI, 61.5%-83.7%). The 12-month OS rate was 43.0% (95% CI, 31.9%-58.0%). The median OS rate was 10.6 months (95% CI, 8.4-12.8 months) for the intention-to-treat population of 59 patients (Figure 2). The median PFS by investigator assessment was 5.4 months (95% CI, 4.2-6.6 months), with no difference by PDL-1 expression (eFigure 1 in Supplement 1).

**Figure 3.**
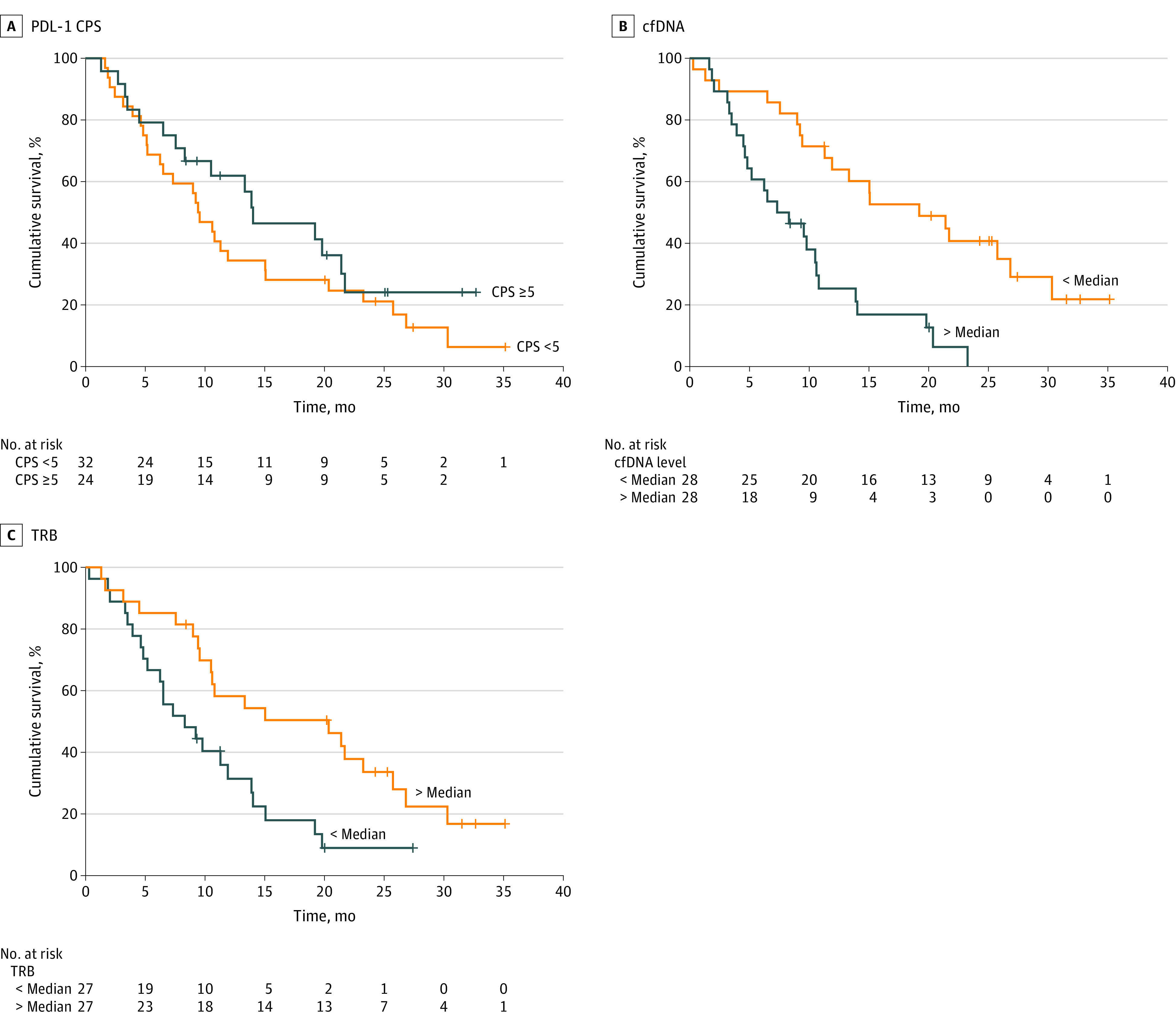
Overall Survival Rate in Subgroups A, Survival is presented in the subgroup by a programmed cell death ligand 1 (PD-L1) combined positive score (CPS) of less than 5 vs 5 or greater, determined from archival tissue by central pathology. B, Survival is presented by cell-free DNA (cfDNA) level determined at baseline, grouped as less than or greater than the median. C, Survival is presented by T cell receptor repertoire richness (TRB) determined in peripheral blood at baseline, grouped as less than or greater than the median.

In subgroups of patients assessable by central pathology with a PDL-1 CPS less than 5 (32 patients) and 5 or greater (24 patients), the median OS was 9.4 months (95% CI, 7.2-11.7 months) vs 14.0 months (95% CI, 6.0-22.1 months; hazard ratio, 0.70; 95% CI, 0.38-1.29; *P* = .25) (Figure 3A). In an independent central radiology review among 50 patients, an objective response was seen in 13 patients (26.0%) and disease control in 41 patients (82.0%) (complete response, 2 patients [4.0%]; partial response, 11 patients [22.0%]; stable disease, 28 patients [56.0%]; progressive disease, 9 patients [18.0%]). A waterfall plot illustrates individual responses (eFigure 2 in Supplement 1). A total of 31 patients (52.5%) received any postdiscontinuation therapy. There was no association of microsatellite instability (4 patients), HER2-positive status (6 patients), or Epstein-Barr virus–positive status (2 patients) with response. However, data are not informative owing to small numbers. In a multivariate analysis, cfDNA levels less than the median and TRB greater than the median showed independent associations with improved OS (eTable 5 in Supplement 1).

### Translational Research

We analyzed tumor tissue and peripheral blood for known cancer-related mutations (eFigure 3 in Supplement 1). The median time between tissue sampling (primary diagnosis) and liquid biopsy taken at baseline was 9.7 months (95% CI, 8.5-11.8 months). In 46 of 58 patients for whom mutational analyses could be performed (79.3%), cancer-related mutations could be identified. Comparing peripheral blood with tumor tissue in 32 patients for whom results for both outcomes were available, concordant mutations were detected in 16 patients (50.0%), indicating a high degree of clonal heterogeneity and presumably evolution (eFigure 3 in Supplement 1). Mutations were identified only from liquid biopsy in 5 patients (15.6%) and only from tumor DNA in 4 patients (12.5%). Of 378 mutations in these 32 patients, 31 mutations (8.2%) were detectable in tumor tissue and peripheral blood, 98 mutations (26.9%) were detectable only in the tumor, and 249 mutations (65.9%) were detectable only in the peripheral blood.

In 58 patients, cfDNA concentrations could be determined from peripheral blood samples at baseline. An association was found between the baseline level of cfDNA prior to treatment initiation and OS. Patients with cfDNA levels greater than and less than the median (eFigure 4 in Supplement 1) showed a significantly different median OS of 7.3 months (95% CI, 3.2-11.4 months) compared with 19.2 months (95% CI, 8.9-29.6 months; hazard ratio, 0.30; 95% CI, 0.16-0.59; *P* < .001) (Figure 3B) and median PFS of 4.5 months (95% CI, 2.9-6.2 months) vs 7.3 months (95% CI, 4.6-10.1 months) (*P* = .006) (eFigure 1 in Supplement 1).

In 54 patients, TRB was analyzed in peripheral blood at baseline. For each patient blood sample, TRB richness was calculated as a surrogate parameter for immune competence.^17,18^ In this group of patients pretreated for cancer, median (range) TRB richness was 1217 (317-2840) unique T cell clones per sample identified in 250 ng of leukocyte DNA (eFigure 4 in Supplement 1). Patients with TRB richness greater than and less than the median showed a significantly different median OS (OS, 20.4 months; 95% CI, 7.7-33.0 months vs 8.3 months; 95% CI, 3.7-12.9 months; hazard ratio; 0.43; 95% CI, 0.23-0.81; *P* = .008) (Figure 3C) and median PFS (6.6 months; 95% CI, 4.7-8.6 months vs 4.6 months; 95% CI, 4.0-5.2 months) (*P* = .04) (eFigure 1 in Supplement 1).

## Discussion

In this single-group, multicenter, phase 2 nonrandomized controlled trial, we explored the efficacy and tolerability of the combination of the checkpoint inhibitor avelumab and standard of care second-line therapy paclitaxel plus ramucirumab, exploiting possible synergism between PDL-1 inhibition and a VEGFR-2 antibody. The trial started before checkpoint inhibitors were available in the first-line setting in PDL-1–positive disease; therefore, all patients in our trial were CPI naive. Today, all patients with PDL-1–positive disease (CPS ≥1 in the US; CPS ≥5 in Europe) have the option to be treated with CPI in the first line. Now, there is high medical need to explore CPI for PDL-1–low or –negative tumors and for tumors beyond progression under CPI. Establishing an efficacious and tolerable regimen in the second line is highly relevant to set the basis for trials attempting to establish checkpoint inhibitors in tumors with low or no expression of PDL-1 or to overcome resistance to checkpoint inhibitors by evaluating treatment beyond progression.

In the RAP trial, we treated 59 patients who had EGA with ramucirumab, avelumab plus paclitaxel who progressed after platinum plus fluoropyrimidine therapy. A significant proportion of patients (67.8%) had received additional treatment with a taxane in their first-line regimen. We did not see any unexpected toxic effects. Treatment was generally well tolerated. Disease control could be achieved in 79.7% of patients. The median OS for the intention-to-treat population was 10.6 months. Comparing patients with a CPS of 5 or greater vs those with a CPS less than 5, median survival differed clearly (14.0 vs 9.4 months), underlining the previously reported predictive value of PDL-1 CPS in patients with EGA.^19^ Low cfDNA levels before treatment initiation (median OS, 19.2 months) and high TRB (median OS, 20.4 months) were associated with a significantly better survival. Similar associations were observed in a previous immunotherapy trial in patients with EGA performed by the Arbeitsgemeinschaft Internistische Onkologie (AIO) study group,^18^ lending more support to the notion that checkpoint inhibitors add particular benefit to the standard of care, especially in patients with lower cfDNA levels and high TRB.

Compared with published data, our results suggest that the RAP regimen may be very promising, supporting the hypothesis of a synergistic efficacy of the 3 components. In the 2022 RAMIRIS trial of the AIO,^20^ a very similar patient population (100% of patients pretreated with platinum plus fluoropyrimidine and 68% of patients pretreated with taxanes) was treated with second-line ramucirumab plus paclitaxel or FOLFIRI. The median OS in RAMIRIS was 7.6 months, which was shorter than for patients with a PDL-1 CPS less than 5 in the RAP study (9.4 months). In the Western population of the RAINBOW trial, in which patients were pretreated with platinum plus fluoropyrimidine but not with taxanes, the median OS was 8.6 months,^15^ also 2 months shorter than in the RAP trial, which had mainly patients treated with taxane.

Synergism between VEGF inhibition and CPI has been investigated in different trials in gastric cancer. Ramucirumab and pembrolizumab were combined in first-line treatment in 28 patients unselected for their PDL-1 expression level,^11^ demonstrating an overall response rate of 25% (PDL-1 positive: 32%; PDL-1 negative: 17%). In a phase I/II study with an Asian population (43 patients), second-line nivolumab combined with paclitaxel plus ramucirumab showed an overall response rate of 37.2%, a 6-month PFS of 46.5%, and a median OS of 13.1 months.^12^ Analyzing subgroups by CPS, the median OS was 13.8 months in patients with a CPS of 1 or greater and 8.0 months for those with a CPS less than 1. Our trial investigated the addition of the PDL-1 antibody avelumab in a larger, White population. The centrally reviewed PDL-1 status in the RAP study suggests that the synergism was higher in PDL-1–overexpressing tumors, but even in patients with a PDL-1 CPS less than 5, median OS was nearly 2 months longer than in the RAMIRIS study.^20^

Translational research performed in the RAP trial may further help to identify patients who may benefit most from the RAP combination. We analyzed archival tumor tissue and peripheral blood cells for tumor mutations. It is of note that only 50% of patients (16 of 32 with both blood and tumor material available at baseline) had at least 1 identical mutation identified from tumor DNA and cfDNA. In 5 of 32 patients (15.6%), mutations were identified only from liquid biopsy, and in 4 patients (12.5%), mutations were identified only from tumor DNA. Of 378 mutations in 32 patients with matching materials, 32 mutations (8.2%) were detectable in tumor tissue and peripheral blood, 98 mutations (26.9%) were detectable only in the tumor, and 249 mutations (65.9%) were detectable only in the peripheral blood. These findings suggest that different tumor subclones may rapidly emerge in gastric cancer. The high degree of mutational heterogeneity is not unexpected given that tumor material was acquired in most patients at the time of diagnosis while the liquid biopsy was performed before initiation of second-line treatment (median time between tissue collection and liquid biopsy was 9.7 months). Rapid clonal evolution is not an isolated finding given that several trials have reported similar results, specifically in gastric cancer.^21,22,23,24^

The high degree of clonal evolution in gastric cancer has led us to examine the amount of cfDNA as a means of assessing the association with the total tumor burden, including all sites and subclones. Our findings showed that high levels of cfDNA were associated with a significantly shorter OS. On the other hand, low baseline levels of cfDNA defined a patient group with a median OS of more than 19 months on the RAP regimen. Another biological marker, high TRB, was identified as a factor associated with favorable outcomes on the RAP regimen. This concept of TRB as a biomarker in cancer treatment has previously been reported based on peripheral blood and tissue-infiltrating T cell analysis.^13,25,26,27,28,29^ It is particularly noteworthy that TRB is influenced by thymic cell output and that high richness likely indicates a higher level of immune fitness.^30^ As such, it is of great interest that high richness was found to be associated with better OS in patients undergoing immune modulatory therapy. However, our single-group trial cannot differentiate between the potential predictive or prognostic significance, and future trials will have to prospectively validate this biomarker.

The RAP trial is a proof-of-concept study that generates hypotheses and is limited by its small patient population and nonrandomized design. Therefore, results can be compared only with historical data from other studies. Despite these limitations, the observed OS of 14, 19, or 20 months in patient subgroups defined by PDL-1 CPS, cfDNA level, or TRB at baseline is highly promising and suggests a substantial synergy among ramucirumab, paclitaxel, and immune-modulatory therapy with avelumab.

### Limitations

There are several limitations to this study: The RAP trial included 59 evaluable patients. Owing to this low number, all results have to be considered as descriptive. There was no comparator group; therefore, survival analyses have to be judged in view of historical control groups. Given that the same AIO study centers included patients under similar inclusion criteria in the AIO RAMIRIS trial, this study may serve as an informative cohort to our RAP trial. Comparing our results with this cohort’s OS rate suggests favorability using the RAP combination. Another limitation of our study is that our translational analyses were restricted to baseline data. Translational analyses of patient samples under treatment would be beneficial for future trials.

## Conclusions

In this nonrandomized controlled trial evaluating the addition of immune CPI to the second-line standard therapy, the combination of ramucirumab, avelumab, and paclitaxel was well tolerated and highly effective in the second-line treatment of a patient population with heavy pretreatment. This study provides valuable insights into how to optimize treatment regimens that include checkpoint inhibitors, even for patients who have low PDL-1 expression or have progressed after previous checkpoint inhibitor treatment. Our findings suggest that T cell repertoire metrics and cfDNA levels may have the potential to become new markers that can help select patients for therapy. Future trials will be necessary to further explore the role of checkpoint inhibitors beyond progression and to determine the best combination partners to overcome or prevent resistance.

## Notes

Supplement 1. **eTable 1.** List of Participating Centers **eFigure 1.** Progression-Free Survival **eFigure 2.** Waterfall Plot **eFigure 3.** Mutations Detected in Liquid Biopsy and Tumor Tissue **eFigure 4.** Visualization of Range and Median of Cell-Free DNA and T Cell Receptor β Values **eTable 2.** Translational Research Method Description **eTable 3.** List of Genes and Lengths of Covered Regions in Custom DNA Next-Generation Sequencing Panel **eTable 4.** Adverse Events **eTable 5.** Multivariate Analysis of Prognostic Factors: Univariable and Multivariable Analysis of Overall Survival Rate (Cox Model)

Supplement 2.
Trial Protocol and Statistical Analysis Plan

Supplement 3.
Data Sharing Statement
