# Supplementary material for: Ramucirumab, Avelumab, and Paclitaxel as Second-Line Treatment in Esophagogastric Adenocarcinoma: The Phase 2 RAP (AIO-STO-0218) Nonrandomized Controlled Trial
Source: JAMA Netw Open. 2024 Jan 23;7(1):e2352830. doi: 10.1001/jamanetworkopen.2023.52830 (PMC10807255; doi:10.1001/jamanetworkopen.2023.52830)
Supplement: Supplement 1. — eTable 1. List of Participating Centers eFigure 1. Progression-Free Survival eFigure 2. Waterfall Plot eFigure 3. Mutations Detected in Liquid Biopsy and Tumor Tissue eFigure 4. Visualization of Range and Median of Cell-Free DNA and T Cell Receptor β Values eTable 2. Translational Research Method Description eTable 3. List of Genes and Lengths of Covered Regions in Custom DNA Next-Generation Sequencing Panel eTable 4. Adverse Events eTable 5. Multivariate Analysis of Prognostic Factors: Univariable and Multivariable Analysis of Overall Survival Rate (Cox Model) [file jamanetwopen-e2352830-s001.pdf]

Supplemental Online Content

Thuss-Patience P, Högner A, Goekkurt E, et al. Ramucirumab, avelumab, and paclitaxel as second-line treatment in esophagogastric adenocarcinoma: the phase 2 RAP (AIO-STO-0218) nonrandomized controlled trial. *JAMA Netw Open*. 2024;7(1):e2352830. doi:10.1001/jamanetworkopen.2023.52830

eTable 1. List of Participating Centers

eFigure 1. Progression-Free Survival

eFigure 2. Waterfall Plot

eFigure 3. Mutations Detected in Liquid Biopsy and Tumor Tissue

eFigure 4. Visualization of Range and Median of Cell-Free DNA and T Cell Receptor β Values

eTable 2. Translational Research Method Description

eTable 3. List of Genes and Lengths of Covered Regions in Custom DNA Next-Generation Sequencing Panel

eTable 4. Adverse Events

eTable 5. Multivariate Analysis of Prognostic Factors: Univariable and Multivariable Analysis of Overall Survival Rate (Cox Model)

This supplemental material has been provided by the authors to give readers additional information about their work.

© 2023 Thuss-Patience P et al. *JAMA Network Open.*

eTable 1. List of Participating Centers

Center	Principal Investigator	Included patients No.
Charité-University Medicine Berlin, Dept. of Hematology, Oncology and Cancer Immunology, Berlin, Germany;	Peter Thuss-Patience	16
Hematology-Oncology Practice Eppendorf (HOPE), Hamburg;	Alexander Stein	22
Department of Medical Oncology, Evang. Kliniken Essen-Mitte	Michael Stahl	5
Hematology-Oncology Practice MVZ Mitte, Leipzig	Albrecht Kretzschmar	5
Institute of Clinical Cancer Research (IKF) at Krankenhaus Nordwest, UCT-University Cancer Center, Frankfurt	Thorsten Götze	3
Leipzig University Cancer Center, Leipzig University Hospital	Florian Lordick	2
Sarcoma Center Berlin-Brandenburg, Helios Klinikum Berlin-Buch	Peter Reichardt	2
Department of Medicine I, Hospital Weiden	Frank Kullmann	2
Department of Oncology and Palliative Care, Helios Klinikum Bad Saarow,	Daniel Pink	1
Department of Internal Medicine IV, University Hospital Halle, Martin-Luther University Halle-Wittenberg	Mascha Binder	2

© 2023 Thuss-Patience P et al. *JAMA Network Open.*

Supplementary Fig S1 A

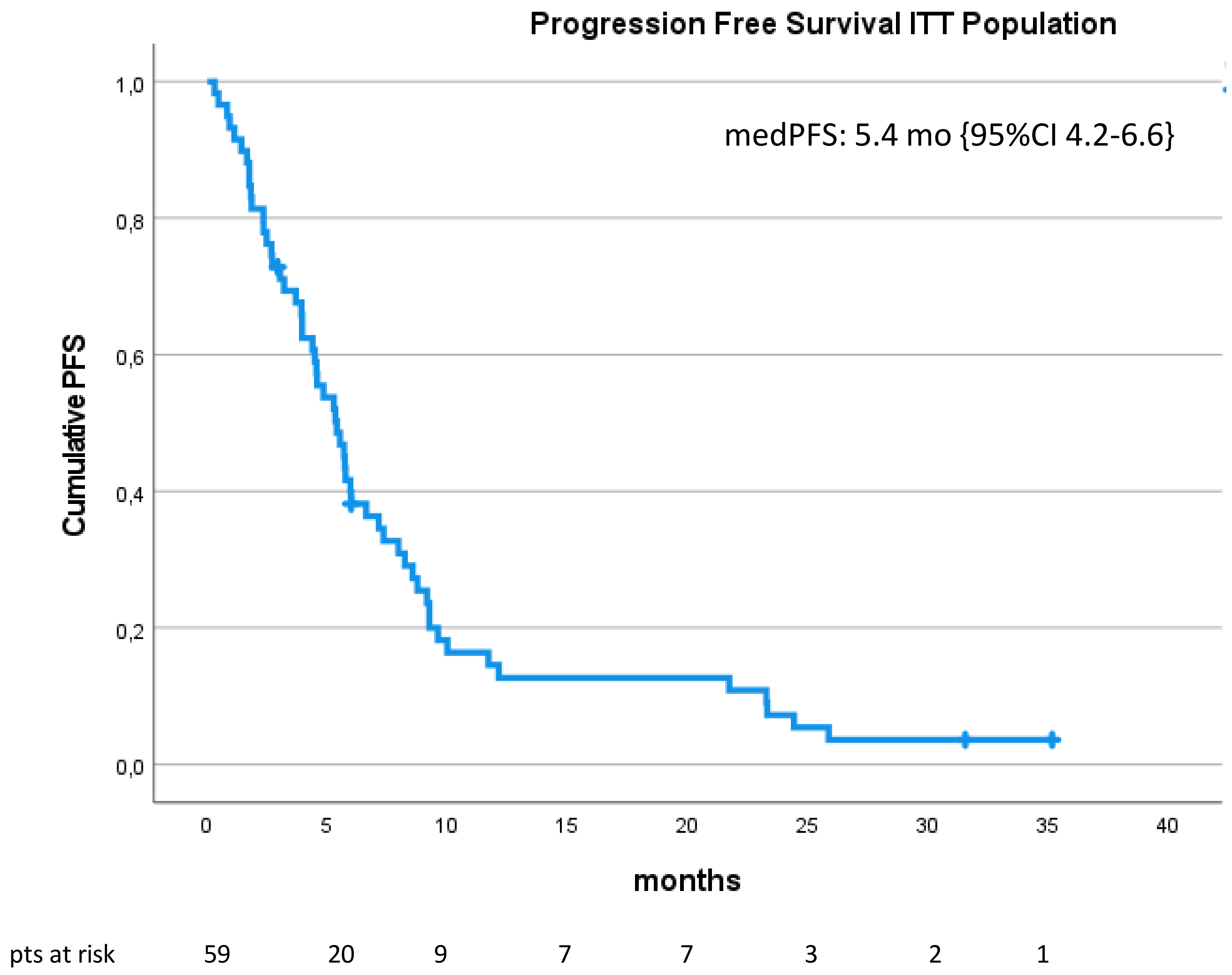

© 2023 Thuss-Patience P et al. *JAMA Network Open.*

Supplementary Fig S1 B

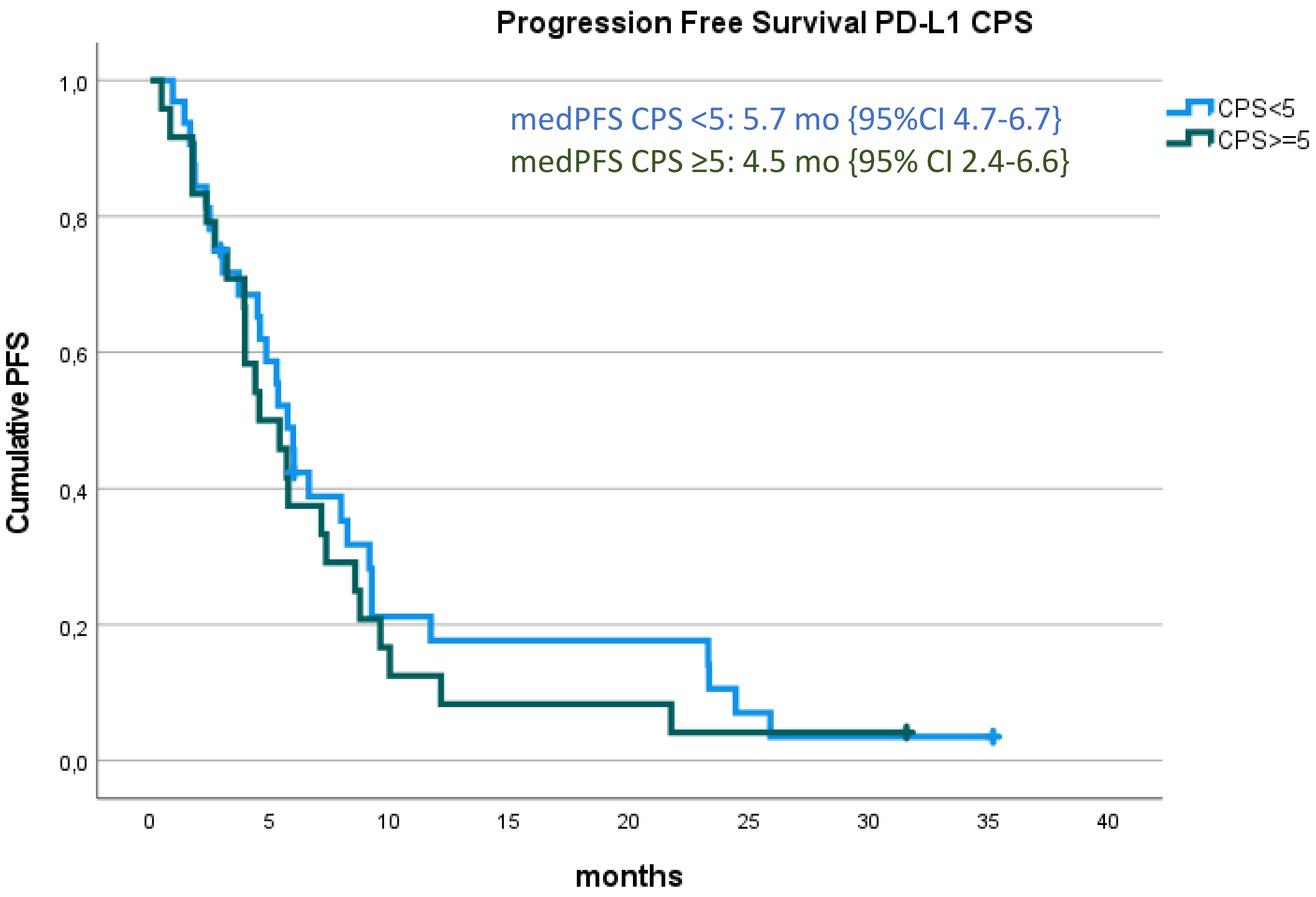

pts at risk								
CPS <5	32	18	6	5	5	2	1	1
CPS ≥5	24	12	3	2	2	1	1	

© 2023 Thuss-Patience P et al. *JAMA Network Open.*

Supplementary Fig S1 C

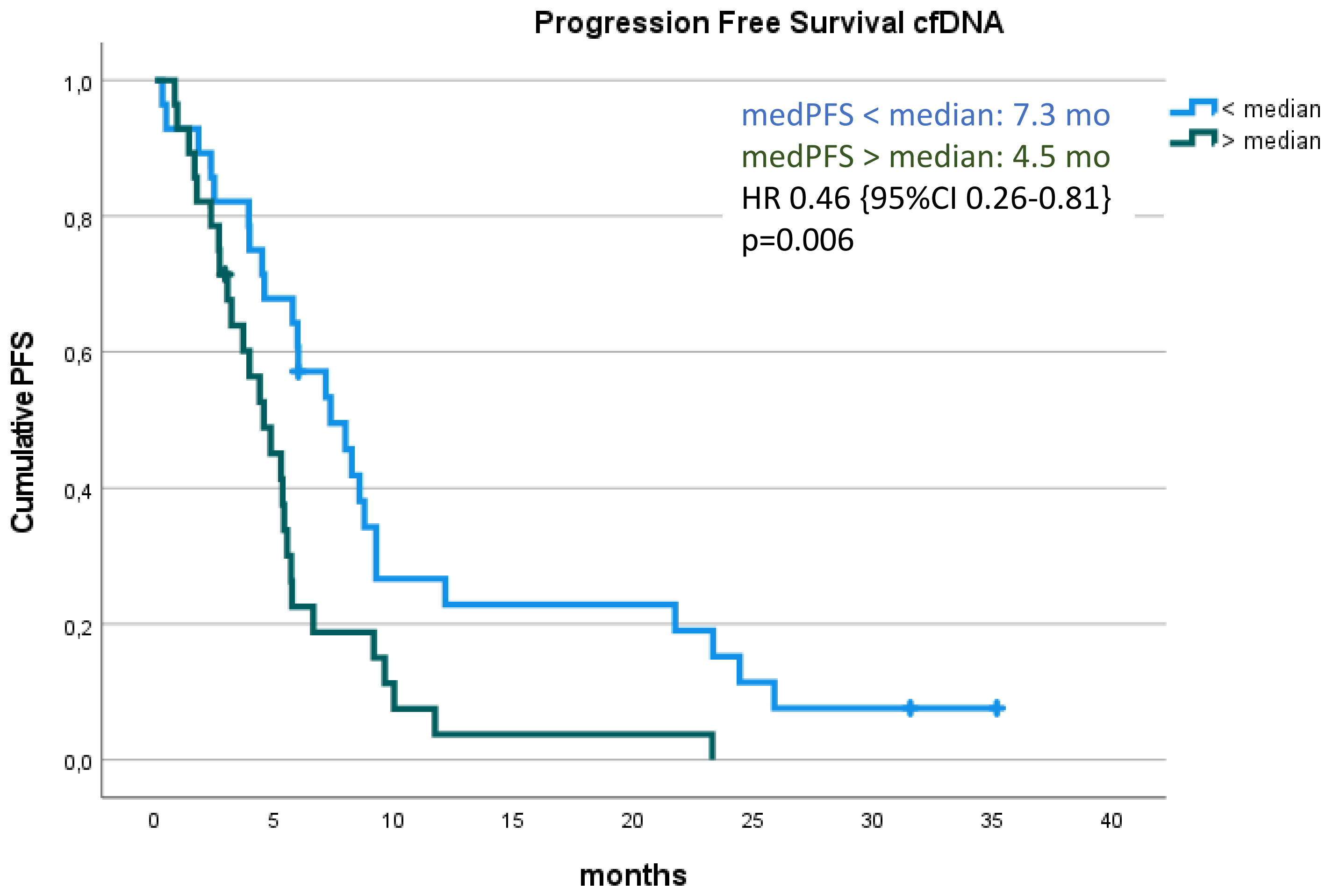

pts at risk								
cfDNA < med	28	19	7	6	6	3	2	1
cfDNA > med	28	12	2	1	1	0	0	0

© 2023 Thuss-Patience P et al. *JAMA Network Open.*

Supplementary Fig S1 D

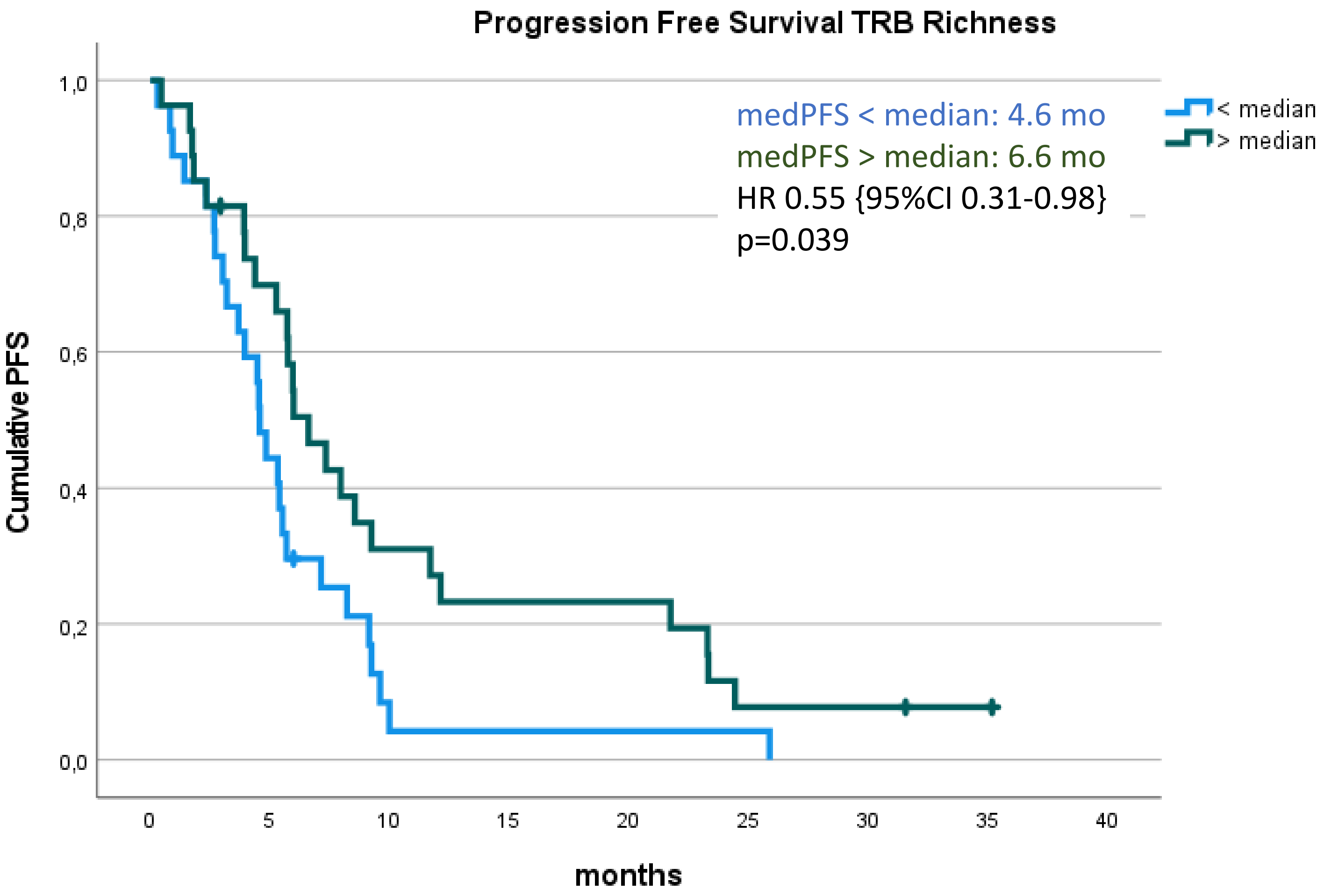

pts at risk								
TRB < med	27	12	1	1	1	1	0	0
TRB > med	27	18	8	6	6	2	2	1

© 2023 Thuss-Patience P et al. *JAMA Network Open.*

Supplementary Fig S2

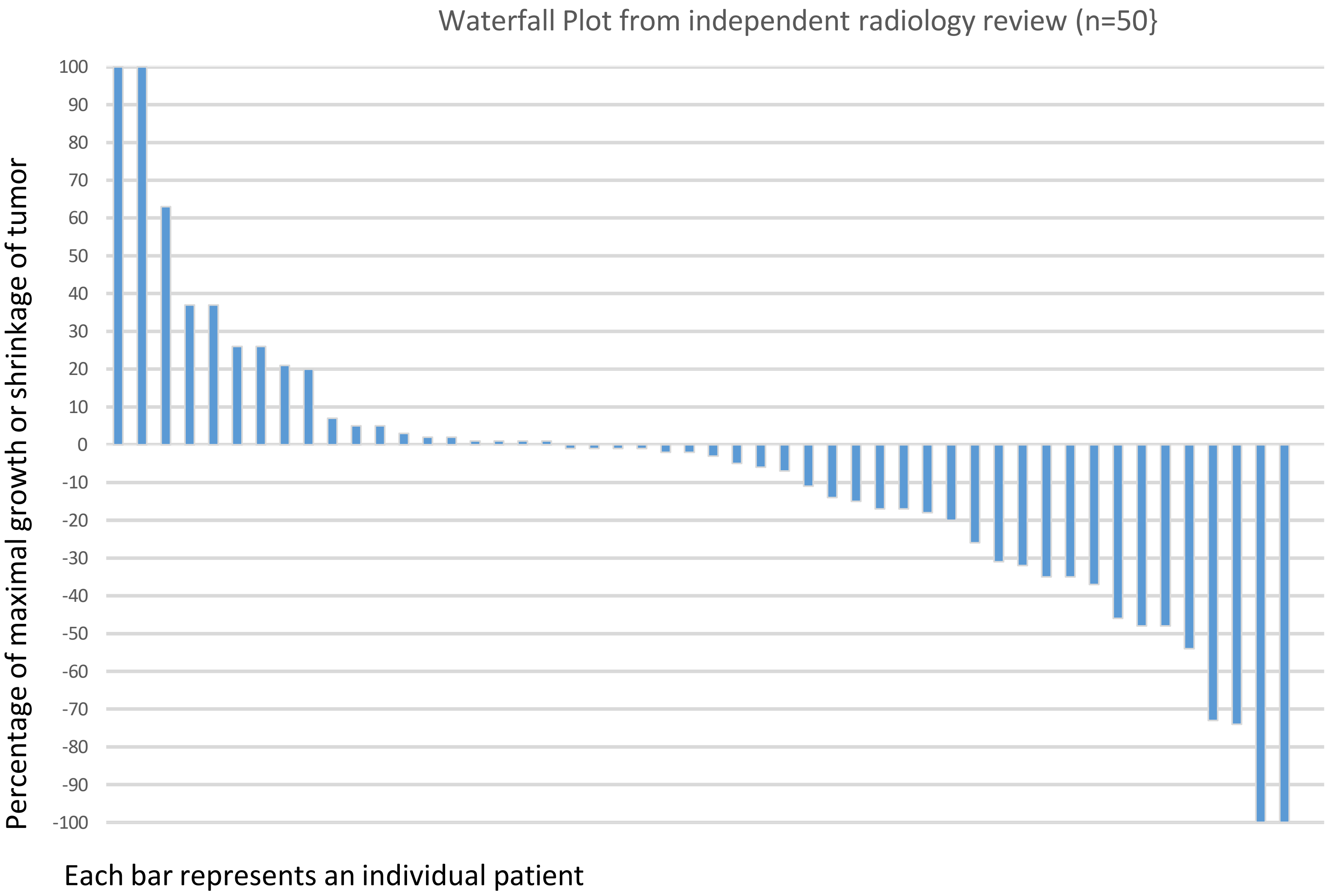

© 2023 Thuss-Patience P et al. *JAMA Network Open.*

Supplementary Fig S3

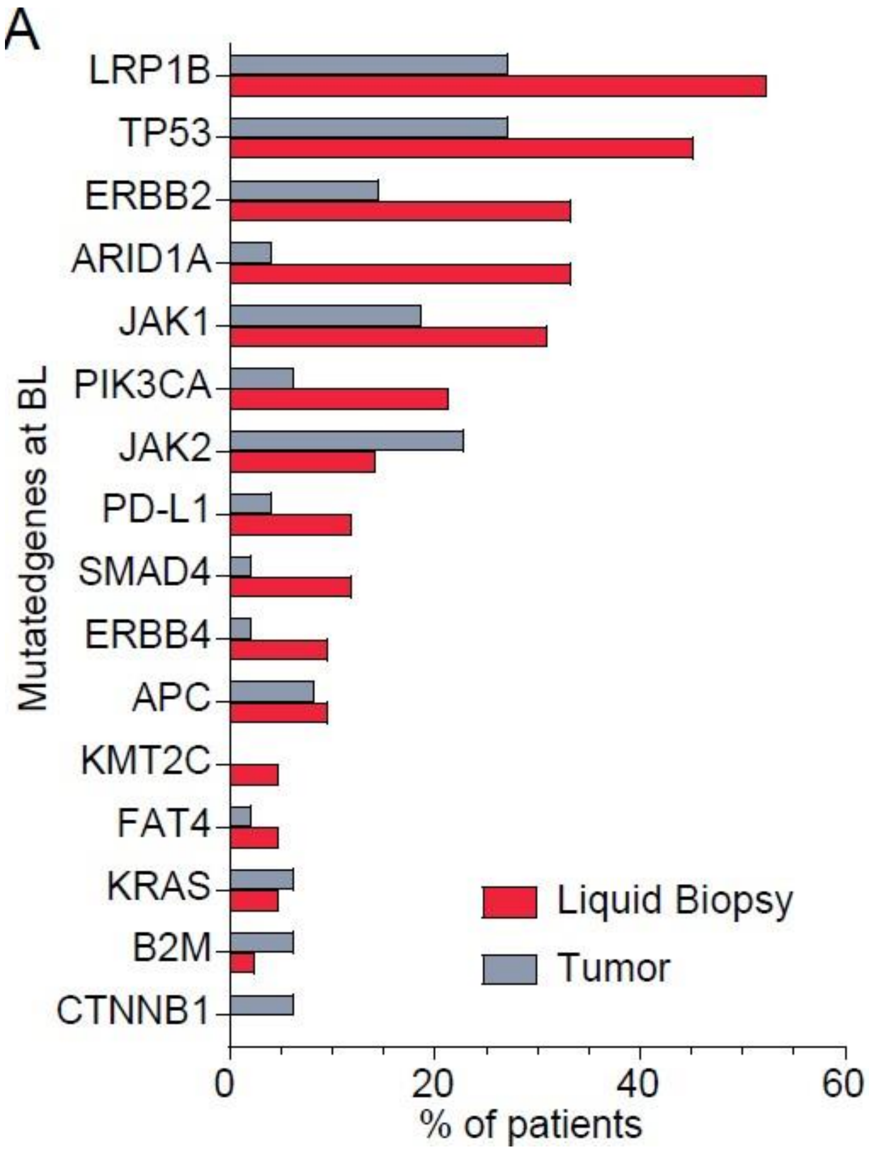

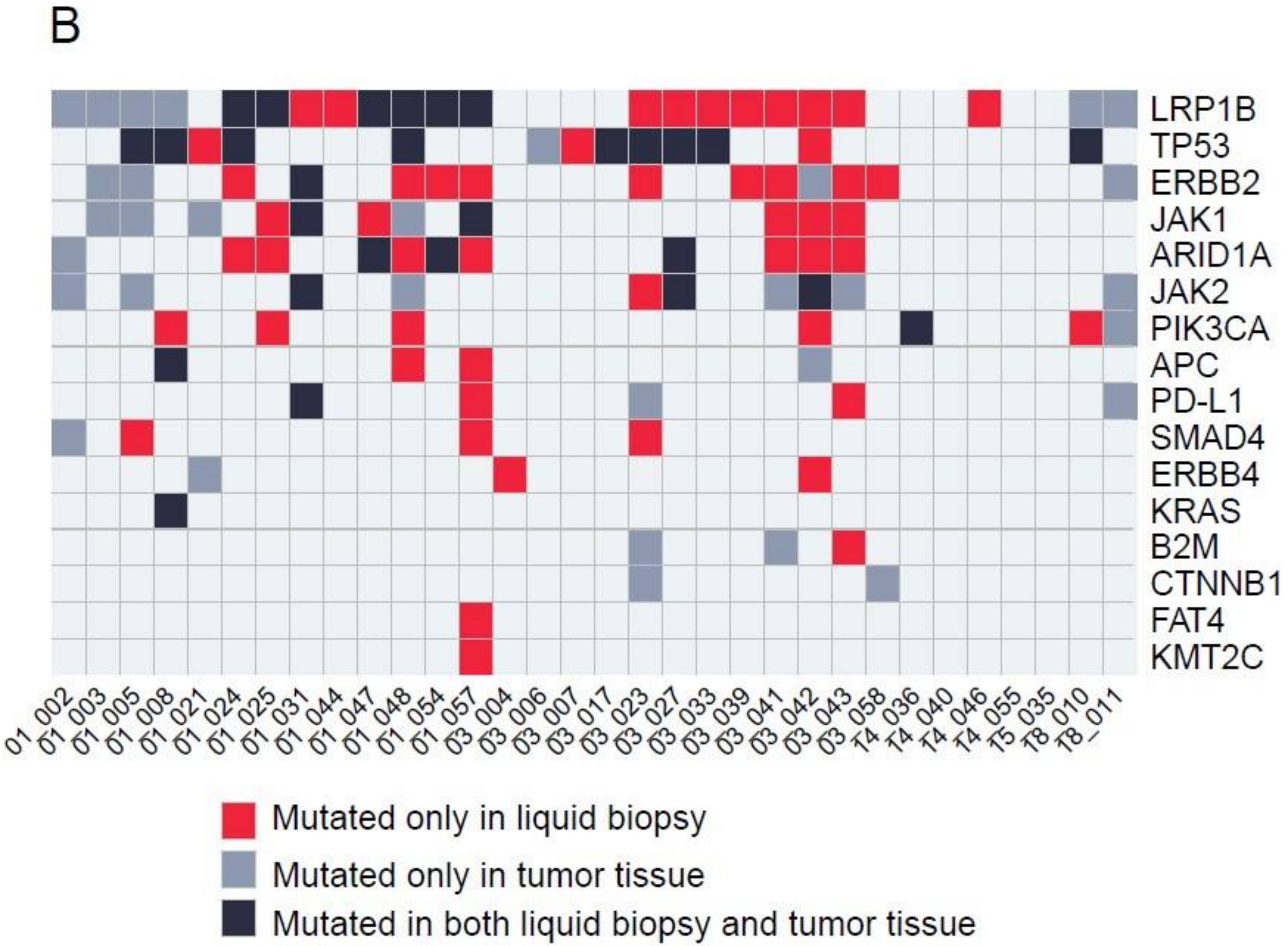

© 2023 Thuss-Patience P et al. *JAMA Network Open.*

Supplementary Fig S4 A

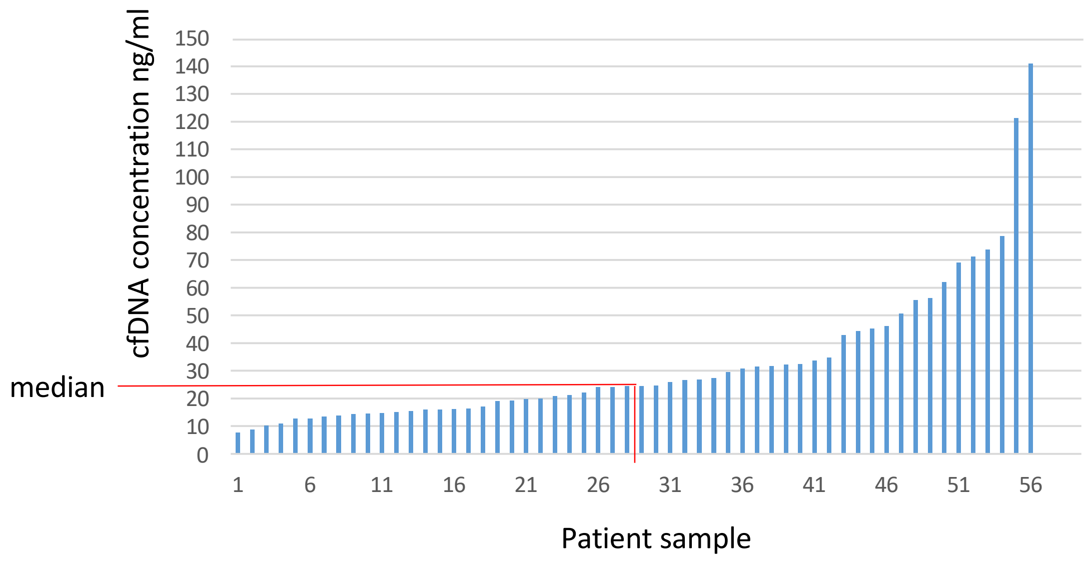

cfDNA at baseline (ng/ml) n=56, median = 24.35 ng/ml, range 7.7-141 ng/ml

© 2023 Thuss-Patience P et al. *JAMA Network Open.*

Supplementary Fig S4 B

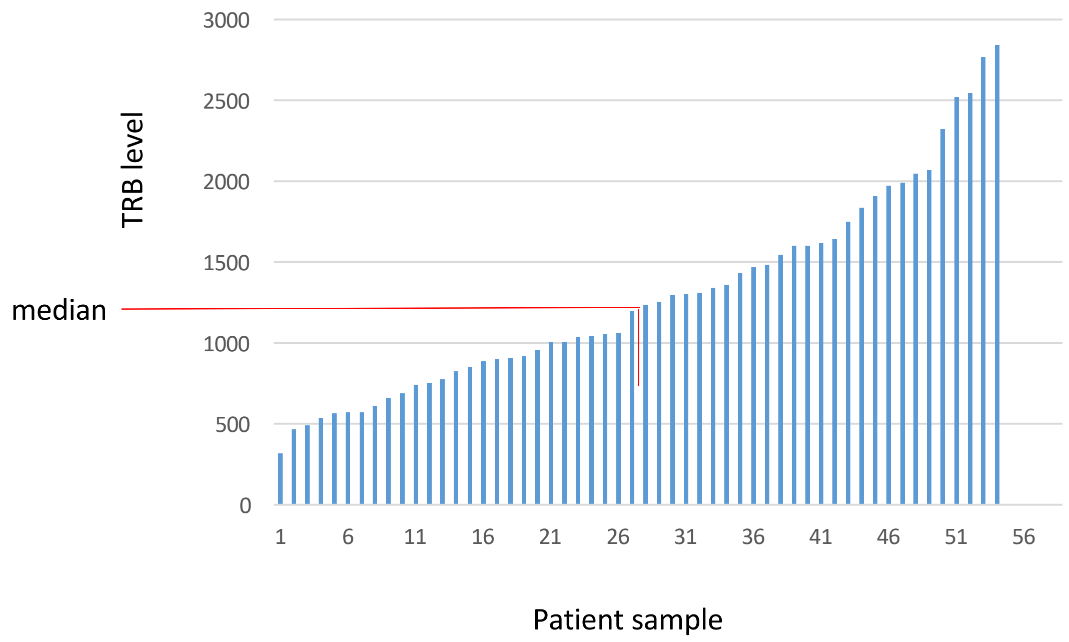

T-cell receptor beta (TRB) richness in liquid biopsy at baseline n=54, median = 1217 unique T-cell clones per sample, range 317-2840

© 2023 Thuss-Patience P et al. *JAMA Network Open.*

Supplementary Table S2

Translational research method description

cfDNA	Mutational analysis	T cell repertoire richness
Peripheral blood was collected in Cell-Free DNA BCT tubes (Streck, La Vista, Nebraska, USA} }. Plasma was obtained after centrifugation and taken for isolation of cfDNA using QIAamp Circulating Nucleic Acid Kit (QIAGEN, Hilden, Germany}. The amount of cfDNA per mL blood plasma was quantified on a Qubit system (ThermoFisher Scientific, Waltham, Massachusetts, USA} with high sensitivity reagents. Genomic DNA was extracted from paraffin-embedded tumor material using Maxwell® 16 FFPE Plus DNA Kit (Promega, Walldorf, Germany}.	A set of selected gene regions covering the most frequent mutation hotspots in gastric cancer (Supplementary Table S2} was amplified from 100 ng cfDNA or gDNA via targeted NGS using a QIAseq custom panel from QIAGEN (Hilden, Germany} and following the supplier's protocol. Sequencing and demultiplexing was performed as a dual-indexed run with 300 cycles on an Illumina Nextseq 550 instrument (Illumina, San Diego, California, USA}. Fastq files were processed, filtered and annotated on the CLC workbench (QIAGEN, Hilden, Germany}.	For TRB immunosequencing, genomic DNA was isolated from blood cell pellets using GenElute Mammalian Genomic DNA Miniprep Kit (Sigma, St. Louis, Missouri, USA}. Amplicon-based library generation, sequencing and file processing was performed as described from 250 ng of input leukocyte DNA [14,15]. As reference for sequence alignment, the default MiXCR library was used for TRB sequences. Each unique complementarity-determining region 3 nucleotide sequence was considered a clone. Non-productive reads and sequences with less than 2 read counts were not considered for further bioinformatics evaluation. TRB richness was defined as the total number of clones in a distinct blood sample.

© 2023 Thuss-Patience P et al. *JAMA Network Open.*

Supplementary Table S3

List of genes and lengths of covered regions in QIAseq custom DNA NGS panel.

Gene	Number of bp covered
FCGR3A	106
TP53	1282
B2M	390
ERBB2	3938
PIK3CA	437
SMAD4	577
ERBB4	1048
ARID1A	1898
FAT4	264
LRP1B	14713
KMT2C	247
FCGR2A	107
KRAS	110
CD274	933
JAK2	3629
CTNNB1	372
APC	1158
JAK1	3705

© 2023 Thuss-Patience P et al. *JAMA Network Open.*

Supplementary Table S4

Adverse events.

*CTC grade 5 due to an esophago-tracheal fistula

**Characteristic**				
**CTC Grade**	**1**	**2**	**3**	**4**
	**Patients, No. (%)**	**Patients, No. (%)**	**Patients, No. (%)**	**Patients, No. (%)**
**Hematologic AEs**				
Anemia	3 (5.)	8 (13.6)	3 (5.1)	-
Leucopenia	4 (6.8%)	6 (10.2%)	7 (11.9%)	-
Neutropenia	2 (3.4%)	4 (6.8%)	11 (18.6%)	3 (5.1%)
Anorexia	10 (16.9%)	11 (18.6%)	-	-
Dysphagia	7 (11.9%)	1 (1.7%)	2 (3.4%)	-
Nausea	13 (22.0%)	5 (8.5%)	2 (3.4%)	-
**Non hematologic AEs**				
Vomiting	6 (10.2%)	1 (1.7%)	1 (1.7%)	-
Diarrhea	15 (25.4%)	6 (10.2%)	2 (3.4%)	-
Constipation	7 (11.9%)	4 (6.8%)	-	-
Ascites	1 (1.7%)	2 (3.4%)	1 (1.7%)	-
Fatigue	24 (40.7%)	9 (15.3%)	1 (1.7%)	-
Alopecia	18 (30.5%)	5 (8.5%)	-	-
Mucositis oral	9 (15.3%)	4 (6.8%)	1 (1.7%)	-
Subileus	-	-	1 (1.7%)	-
leus tumor related	-	-	1 (1.7%)	-
Pain	19 (32.2%)	9 (15.3%)	6 (10.2%)	-
Dyspnoea	10 (16.9%)	6 (10.2%)	-	-
Allergic reaction	-	2 (3.4%)	-	-
Change in taste	11 (18.6%)	3 (5.1%)		-
Vision disorders	6 (10.2%)	2 (3.4%)	1 (1.7%)	-
Hearing impaired	-	1 (1.7%)	-	-
Peripheral neuropathy	10 (16.9%)	15 (25.4%)	6 (10.2%)	-
Dizziness	11 (18.6%)	1 (1.7%)	1 (1.7%)	-
Hypertension	3 (5.1%)	2 (3.4%)	4 (6.8%)	-
Fever	6 (10.2%)	4 (6.8%)	-	-
Non-neutropenic systemmic infections	-	5 (8.5%)	6 (10.2%)	(1(1.7%) CTC 5°)*
Neutropenic systemic infections	-	-	1 (1.7%)	1 (1.7%)
Soft tissue infection		1 (1.7%)	2 (3.4%)	-
Acute kidney injury				1 (1.7%)
Atrial fibrillation	-	-	2 (3.4%)	-
Thromboembolic event	-	1 (1.7%)	2 (3.4%)	-
Thrombophlebitis	-	1 (1.7%)	-	-
Hematemesis	-	1 (1.7%)	-	1 (1.7%)
Edema	7 (11.9%)	4 (6.8%)	-	-
**Laboratory abnormalities**				
Hypokalaemia	1 (1.7%)	1 (1.7%)	-	-

© 2023 Thuss-Patience P et al. *JAMA Network Open.*

Elevated CRP	2 (3.4%)	3 (5.1%)	2 (3.4%)	-
Elevated Creatinine	1 (1.7%)	2 (3.4%)	-	-
Elevated Bilirubin	-	1 (1.7%)	1 (1.7%)	
Elevated Alkaline phosphatase	-	1 (1.7%)		
Elevated yGT	-	1 (1.7%)	2 (3.4%)	
Elevated AST	-	-	1 (1.7%)	
Elevated ALT	-	-	1 (1.7%)	
Elevated NTproBNP	-	1 (1.7%)	-	
hypoalbuminemia	-	1 (1.7%)	-	
Elevated leukocyturia	2 (3.4%)	1 (1.7%)	-	
Elevated proteinuria	-	1 (1.7%)	-	
Haematuria	-	-	1 (1.7%)	
**Immune-related AEs**				
Generalised muscle weakness	10 (16.9%)	1 (1.7%)	1 (1.7%)	
Rash maculo papular	6 (10.2%)	1 (1.7%)	-	
Rash acneiform	5 (8.5%)	2 (3.4%)	-	
Pruritus	2 (3.4%)	-	-	
Hypophysitis	-	-	1 (1.7%)	
Hypothyroidism	5 (8.5%)	4 (6.8%)	-	
Hyperthyroidism	1 (1.7%)	3 (5.1%)	-	
Hyperglycemia	-	-	1 (1.7%)	

List of all adverse events reported, irrespective of any causal relation to the study medication. CTC = common toxicity criteria; *CTC grade 5 due to an esophao-tracheal fistula

© 2023 Thuss-Patience P et al. *JAMA Network Open.*

Supplementary Table S5

Multivariate Analysis of Prognostic Factors: Univariable and multivariable analysis of OS (Cox model}

**Prognostic factor*** (univariable p, logrank}, HR	**Statistical parameter**	**full model (n = 54***)**	**reduced model** (n = 54***)**	
**Age**	Hazard ratio	--	--	
**≥ 65 y**	95% confid. interval			
(0.32} HR 1.33 (0.75-2.38}	p			
**ECOG**	Hazard ratio	1.64	--	
**Score = 1**	95% confid. interval	0.87 – 3.09		
(0.044} HR 1.84 (1.01-3.38}	p	0.13		
**Tumor localization**	Hazard ratio	--	--	
**Gastric body**	95% confid. interval			
(0.13} HR 1.56 (0.88-2.78}	p			
**Histology**	Hazard ratio	--	--	
**Diffuse / mixed**	95% confid. interval			
(0.19} HR 1.47 (0.82-2.64}	p			
**Taxane pre-treatment**	Hazard ratio	--	--	
**yes**	95% confid. interval			
(0.26} HR 0.71 (0.39-1.29}	p			
**CPS**	Hazard ratio	--	--	
**≥ 5**	95% confid. interval			
(0.25} HR 0.70 (0.38-1.29}	p			
**cfDNA**	Hazard ratio	2.86	2.92	
**> median**	95% confid. interval	1.40 – 5.82	1.46 – 5.86	
(0.00022} HR 3.28 (1.70-6.35}	p	0.0038	0.0025	
**TRB**	Hazard ratio	0.56	0.56	
**> median**	95% confid. interval	0.28 – 1.10	0.29 – 1.08	
(0.0079} HR 0.43 (0.23-0.81}	p	0.094	0.084	

* The provided category denotes the group for which the relative risk is calculated relative to the complementary reference group. HR > 1.0 corresponds to a higher risk.

** After stepwise elimination of parameters with p > 0.1.

*** 5 patients excluded due to missing values.

© 2023 Thuss-Patience P et al. *JAMA Network Open.*
