# Supplementary material for: Ramucirumab, Avelumab, and Paclitaxel as Second-Line Treatment in Esophagogastric Adenocarcinoma: The Phase 2 RAP (AIO-STO-0218) Nonrandomized Controlled Trial
Source: JAMA Netw Open. 2024 Jan 23;7(1):e2352830. doi: 10.1001/jamanetworkopen.2023.52830 (PMC10807255; doi:10.1001/jamanetworkopen.2023.52830)
Supplement: Supplement 2. — Trial Protocol and Statistical Analysis Plan [file jamanetwopen-e2352830-s002.pdf]

Investigational Plan & Clinical Study Protocol

Avelumab + Paclitaxel/ Ramucirumab as second line treatment in gastro-esophageal adenocarcinoma: a phase II trial of the AIO

The RAP-Trial

Study of the Charité Comprehensive Cancer Center (CCCC) and
the Arbeitsgemeinschaft internistische Onkologie (AIO)

Charité-University Medicine Berlin
Medizinische Klinik mit Schwerpunkt Hämatologie, Onkologie u. Tumorimmunologie
Augustenburger Platz 1
13353 Berlin

Legal Sponsor: Charité-University Medicine Berlin
Charitéplatz 1
10117 Berlin

EudraCT-Nr.: 2018-002938-20
AIO-Nr.: AIO-STO-0218 (RAP)
Version: 1.3 vom 05.06.2020

Institution Name

Coordinating Investigator	Priv.-Doz. Dr. med. Peter Thuss-Patience<br>Charité-University Medicine Berlin – Campus Virchow Klinikum<br>Med. Klinik m.S. Hämatologie, Onkologie u. Tumorimmunologie<br>Augustenburger Platz 1<br>13353 Berlin<br>Germany<br>phone +49 30 450653193<br>peter.thuss@charite.de
Vice Coordinating Investigator	Priv.-Doz. Dr. med Alexander Stein<br>University Medical Center Hamburg-Eppendorf<br>II. Medizinische Klinik und Poliklinik<br>(Onkologie, Hämatologie, KMT mit Sektion Pneumologie)<br>Hubertus Wald Tumorzentrum - UCCH<br>Martinistr. 52<br>20246 Hamburg<br>Germany<br>a.stein@uke.de
Translational research coordinator	Priv.-Doz. Dr. med Alexander Stein<br>University Medical Center Hamburg-Eppendorf<br>II. Medizinische Klinik und Poliklinik<br>(Onkologie, Hämatologie, KMT mit Sektion Pneumologie)<br>Hubertus Wald Tumorzentrum - UCCH<br>Martinistr. 52<br>20246 Hamburg<br>Germany<br>a.stein@uke.de
Steeringcommittee	Priv.-Doz. Dr. med. Peter Thuss-Patience, Berlin<br>Priv.-Doz. Dr. med Alexander Stein, Hamburg<br>Prof. Dr. Salah-E. Al-Batran, Frankfurt
Legal sponsor	Charité-University Medicine Berlin<br>Charitéplatz 1<br>10117 Berlin<br>Germany
CRO (Data management)	KKS Koordinierungszentrum für Klinische Studien<br>Charité – Universitätsmedizin Berlin<br>Reinickendorfer Straße 61, 13347 Berlin<br>Germany
Monitoring	ClinAssess GmbH Werkstättenstraße 39b, 51379 Leverkusen<br>s.seiffert@clinassess.de
Safety management	PD Dr. med. Peter Thuss-Patience<br>Charité-University Medicine Berlin – Campus Virchow Klinikum<br>Med. Klinik m.S. Hämatologie, Onkologie u. Tumorimmunologie<br>Augustenburger Platz 1<br>13353 Berlin<br>Germany<br>Phone: +49 30 450 653 868 / 889<br>Fax: +49 30 450 553 908<br>magenkarzinom@charite.de

Biostatistics	Dr. Axel Hinke<br>Cancer Clinical Research Consulting<br>Elly-Heuss-Knapp-Str. 36<br>D-40595 Düsseldorf<br>Germany
Drug Supply	Merck KGaA<br>Headquarters of the Merck Group<br>Frankfurter Straße 250<br>64293 Darmstadt<br>Germany
Data Safety Monitoring Board (DSMB)	Dr. Axel Hinke<br>Prof. Dr. med. Hanno Riess<br>PD Dr. med. Markus Schuler

Approval of the Protocol (Version 1.3 of the 05.06.2020)

Signature	Date (DD Month YYYY)
Printed name of the Representative of the Sponsor	
Signature	Date (DD Month YYYY)
Printed name of Coordinating Investigator	
Coordinating Investigator's Institution	

Investigator's Agreement

I have read the attached protocol entitled

"Avelumab + Paclitaxel/ Ramucirumab as second line treatment in gastro-esophageal adenocarcinoma: a phase II trial of the AIO."

Version Final, 05.06.2020 and agree to abide by all provisions set forth therein.

I agree to comply with the International Conference on Harmonisation Tripartite Guideline on Good Clinical Practice (ICH-GCP), the requirements of the AMG, all applicable national regulations as well as the requirements of the appropriate Institutional Review Board/Independent Ethics Committee and any other institutional requirements.

I agree to ensure that the confidential information contained in this document will not be used for any purpose other than the evaluation or conduct of the clinical investigation without the prior written consent of the study sponsor. They only serve to inform the investigators, their staff, the ethics committee, the federal agency, the CRO and to inform the patient.

I consent to report every serious clinical adverse event to the CRO within 24 hours after awareness, whether it is related to study medication or not.

Signature

Date (DD Month YYYY)

Printed name of Investigator

Investigator's Institution

Signature

Date (DD Month YYYY)

Printed name of Deputy

Deputy's Institution

1 TABLE OF CONTENTS

1 TABLE OF CONTENTS 3

2 ABBREVIATIONS 8

3 SYNOPSIS 10

4 INTRODUCTION AND BACKROUND 21
4.1 Background 21
4.2 Medical need for this trial 22
4.3 Expected tolerability 23

5 STUDY OBJECTIVE 25

6 STUDY DESIGN 26
6.1 Primary endpoint 26
6.2 Secondary endpoints 26

7 STUDY POPULATION 27
7.1 Number of patients 27
7.2 Selection criteria 27
7.2.1 Inclusion criteria 27
7.2.2 Exclusion criteria 28

8 STUDY PROCEDURES AND METHODOLOGY 32
8.1 Study schedule overview 32
8.2 Treatment 32
8.2.1 Dosing and schedule 32
8.2.2 Premedication 33
8.2.3 Special precautions for administration 34
8.2.4 Treatment duration 34
8.2.5 Study medication 34
8.2.6 Concomitant medication 34

8.3 Assessment and guidelines for visits ... 38
8.3.1 Baseline assessments ... 39
8.3.2 Assessment during study ... 40
8.3.3 30 days safety follow-up (±7 days) ... 43
8.3.4 Extended safety follow-up ... 43
8.3.5 Follow up ... 44
8.4 Post-study treatment ... 44
8.5 Study Duration ... 44
8.6 Study Termination ... 44
8.6.1 Regular Data Analysis by the Independent Data Monitoring Committee. 44
8.6.2 Patient withdrawal ... 45
8.6.3 Study completion ... 46

9 DOSE MODIFICATION ... 47
9.1 Toxicity at start of the following cycle ... 47
9.2 Toxicity during the cycle ... 48
9.3 Guidelines for dose modifications for avelumab and managment of Avelumab-specific Adverse Events or Adverse Drug Reactions ... 49
9.3.1 Infusion-related reactions ... 49
9.3.2 Severese hypersensitivity reaction and flu-like symptoms ... 50
9.3.3 Tumor lysis syndrome ... 50
9.3.4 Immune-related Adverse Events ... 52
9.4 Expected toxicities of paclitaxel and ramucirumab and guidelines for dose modifications for Ramucirumab ... 58
9.5 Guidelines for dose modifications for paclitaxel ... 60

10 CRITERIA OF EVALUATION ... 61
10.1 Overall Survival Rate at 6 months ... 61
10.2 Overall Survival ... 61
10.3 Overall Survival Rate at 12 months of treatment ... 61
10.4 Progression free survival/Progression Free Survival Rate ... 61
10.5 Response rate ... 61
10.6 Duration of Response ... 62
10.7 Safety endpoints ... 62

11 TRANSLATIONAL RESEARCH 63
11.1 Translational research projects 63
11.2 Sampling time points and materials 63
11.3 Storage and future use of biological samples 65
11.4 Usage of translational data 66

12 ASSESSMENT OF ADVERSE EVENTS 67
12.1 Independent Data Monitoring Committee 67
12.2 Reference safety documents 68
12.3 Adverse event defintion 68
12.3.1 Adverse events 68
12.3.2 Serious adverse events 69
12.3.3 Unexpected adverse events/SUSAR 70
12.3.4 Other reportable events 70
12.4 Assessment of relationship – adverse drug reaction 70
12.4.1 Practical guidance for causality assessments: 71
12.5 Assessment of severity 71
12.6 Safety recording and reporting requirements 72
12.6.1 Recording periods 72
12.6.2 Recording and reporting requirements 72
12.6.3 Sponsor obligations 75
12.7 Handling of safety parameters 76
12.7.1 Adverse events 76
12.7.2 Treatment and follow-up of adverse events 76
12.7.3 Follow-up of abnormal laboratory test values 76
12.7.4 Overdose 77
12.7.5 Pregnancy and contraception 77
12.8 Adverse drug reaction with concomitant medication 80

13 DATA ANALYSIS AND STATISTICAL CONSIDERATIONS 81
13.1 General design and sample size estimation 81
13.2 Analysis populations 82
13.3 Statistical methods 82

14 DATA MANAGEMENT 84

14.1 Patient identification list 84
14.2 Data capture 84

15 QUALITY ASSURANCE 85
15.1 Standardization 85
15.2 Data Access 85
15.3 Monitoring/Source Data Verification (SDV) 85
15.4 Audits and inspections 86

16 REGULATORY AND LEGAL OBLIGATIONS 87
16.1 General provisions/Declaration of Helsinki 87
16.2 Patient protection 87
16.3 Competent authority 87
16.4 Independent Ethics Committee 88
16.5 Amendments 88
16.6 Study reports 89
16.7 Informed consent 89
16.8 Subject confidentiality 90
16.9 Study documentation and archive 91
16.10 Compensation 92

17 TRIAL SPONSORSHIP AND FINANCING 93

18 TRIAL INSURANCE 94

19 TRIAL REGISTRATION 95

20 PUBLICATION POLICY 96

21 APPENDIX 97
21.1 Bibliography 97
21.2 ECOG Performance Status 101
21.3 CTCAE v 5.0 101
21.4 RECIST v1.1 and modified RECIST 101
21.4.1 Definitions of measurable/non-measurable lesions 102

21.4.2 Tumor response evaluation 104

2 ABBREVIATIONS

**Abbreviation/Acronym**	**Definition**
ADR	Adverse Drug Reaction
AE	Adverse event
ANC	Absolute neutrophil count
ALT	Alanine aminotransferase
AST	Aspartate aminotransferase
BSA	Body surface area
CA	Competent authority
CA 19-9	Carbohydrate antigen 19-9
CA 72-4	Carbohydrate antigen 72-4
CEA	Carcinoembryonic antigen
eCRF	Electronic Case Report Form
CrP	C reactive Protein
CT	Computerized tomography
CTCAE	Common Terminology Criteria for Adverse Events
CTx	Chemotherapy
EBV	Epstein Barr Virus
ECG	Electrocardiogram
ECOG	Eastern Cooperative Oncology Group
FDA	Food and Drug Administration (U.S. government agency)
GCP	Good Clinical Practice
GCP-V	Verordnung über die Anwendung der Guten Klinischen Praxis (GCP) bei der Durchführung von klinischen Prüfungen mit Arzneimitteln zur Anwendung am Menschen
IB	Investigatinal Brochure
ICF	Informed consent form
IDMC	Independent Data Monitoring Committee
IEC	Independent ethics committee
IMP	Investigational medicinal product
INR	International normalized ratio
ITT	Intention-to-treat
IV	intravenous

MRI	Magnetic resonance imaging
MSI	Microsatellite Instability
NCI	National Cancer Institute
NYHA	New York Heart Association
OS	Overall survival
PD	Progressive disease
PTT	Partial thromboplastin time
RECIST	Response Evaluation Criteria in Solid Tumors
SADR	Serious adverse drug reaction
SAE	Serious adverse event
SAR	Serious adverse reaction
SDV	Source Data Verification
SLD	Sum of the longest diameters
SmPC	Summary of Product Characteristics
SUSAR	Suspected Unexpected Serious Adverse Reaction
UA	Urinanalysis
ULN	Upper limit of normal
VEGF	vascular endothelial growth factor
WBC	White blood cell count
WOCBP	Women of childbearing potential

3 SYNOPSIS

**Title**	Avelumab + Paclitaxel/ Ramucirumab as second line treatment in gastro-esophageal adenocarcinoma: a phase II trial of the AIO – The RAP-Trial
**Design**	Single arm multicenter phase II trial
**Indication**	Second-line treatment in patients with gastric or gastro-oesohageal junction adenocarcinoma following first-line therapy with platinum and fluoropyrimidine doublet with or without anthracycline, docetaxel or trastuzumab
**Sample Size**	N=59 patients (10% drop out rate is included)
**Study Duration**	Period of subjects signing ICF: 16 months<br>Period from first patient in to last patient visit: 40 months<br>Maximal treatment duration of individual subject: 1 year<br>Maximal follow up: 1 year after treatment discontinuation
**Endpoints**	Primary endpoint:<br>• Overall Survival Rate at 6 months<br>Secondary endpoints:<br>• Overall Survival<br>• OSR at 12 months<br>• Progression Free Survival<br>• Progression Free Survival Rate at 6 and 12 months according to RECIST v1.1<br>• Safety and tolerability (acc. to NCI CTC AE v5.0 and to the obtained data on vital signs, clinical parameters and feasibility of the regimen)<br>• Best response according to RECIST v1.1<br>• Confirmed response rate according to RECIST v1.1<br>• Duration of response<br>• Translational research (correlation of efficacy parameters with immunoprofiling (TCRβ & IgH); tumor-infiltrating lymphocytes (TiL) repertoire determination, quantitative ctDNA and clonal dynamics)<br>• Subgroup analyses of all primary and secondary endpoints in view of PD-L1 status<br>• Efficacy parameters (PFS and ORR) according to modified RECIST

**Trial Overview**	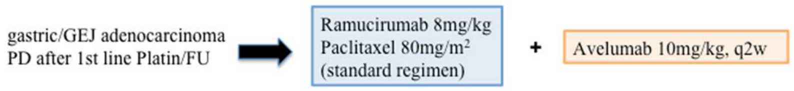
**Inclusion Criteria**	1. Signed written informed consent<br>2. Male or female ≥ 18 years of age<br>3. Histologically proven gastric adenocarcinoma including adenocarcinoma of the esophagogastric junction<br>4. Metastatic or locally advanced disease, not amenable to potentially curative resection<br>5. Documented objective radiological or clinical disease progression during or within 6 months of the last dose of first-line platinum and fluoropyrimidine doublet with or without anthracycline, docetaxel or trastuzumab. Neoadjuvant/adjuvant treatment is not counted unless progression occurs <6 months after completion of the treatment. In these cases neoadjuvant/adjuvant treatment is counted as first line.<br>6. Measurable or non-measurable but evaluable disease determined using guidelines RECIST 1.1<br>7. ECOG performance status 0-1<br>8. Life expectancy > 12 weeks<br>9. Adequate hematological, hepatic and renal functions:<br>a) Absolute neutrophil count (ANC) ≥ 1.5 x 109/L<br>b) Platelet count ≥ 100 x 109/L<br>c) Hemoglobin ≥ 9 g/dl (may have been transfused)<br>d) Total bilirubin ≤ 1.5 times the upper limit of normal (ULN) and AST and ALT ≤ 2.5 x ULN in absence of liver metastases, or ≤ 5 x ULN in presence of liver metastases; AP ≤ 5 x ULN<br>e) Estimated creatinine clearance ≥ 30 mL/min according to the Cockcroft-Gault formula (or local institutional standard method)<br>f) Urinary protein ≤ 1+ on dipstick or routine urinalysis (UA; if urinedipstick or routine analysis is ≥ 2+, a 24-hour urine collection for protein must demonstrate < 1000 mg of protein in 24 hours to allow participation in this protocol)<br>g) Adequate coagulation function as defined by International Normalized Ratio (INR) ≤ 1,5 ULN, and a partial thromboplastin time (PTT) ≤ 5 seconds above the ULN (unless receiving anticoagulation therapy). Patients receiving warfarin/phenprocomon must be switched to low molecular weight heparin and have achieved stable coagulation profile prior to first dose of protocol therapy.<br>10. Women of child-bearing potential must have a negative urine or serum pregnancy test

	11. Highly effective contraception for both male and female subjects throughout the study and for at least 30 days after last avelumab and at least 3 months after last ramucirumab treatment administration if the risk of conception exists<br>12. Ability to comply with scheduled assessments and with management of toxicities.
**Exclusion Criteria**	1. Other tumor type than adenocarcinoma (e.g. leiomyosarcoma, lymphoma) or a second cancer except in patients with squamous or basal cell carcinoma of the skin or carcinoma in situ of the cervix that has been effectively treated. Patients curatively treated for any other malignancy and disease-free for at least 5 years will be discussed with the sponsor before inclusion<br>2. Concurrent chronic systemic immune therapy, chemotherapy, or hormone therapy not indicated in the study protocol<br>3. Previous therapy with, paclitaxel or ramucirumab or pretreatment with a PD-1, PD-L1 inhibitor<br>4. Current treatment with any anti-cancer therapy ≤ 2 weeks prior to study treatment start unless rapidly progressing disease is measured<br>5. Previous exposure to a VEGF or VEGFR inhibitor or any antiangiogenic agent, or prior enrolment in this study<br>6. Major surgical procedure, open biopsy or significant traumatic injury within 4 weeks prior to start of study treatment; anticipation of need for major surgical procedure (e.g. impending bowel obstruction) during the course of the study<br>7. Grade 3-4 GI bleeding within 3 months prior to enrollment<br>8. History of deep vein thrombosis (DVT), pulmonary embolism (PE), or any other significant thromboembolism (venous port or catheter thrombosis or superficial venous thrombosis are not considered “significant”) during the 3 months prior to first dose of protocol therapy<br>9. Cirrhosis at a level of Child-Pugh B (or worse) or cirrhosis (any degree) and a history of hepatic encephalopathy or clinically meaningful ascites resulting from cirrhosis. Clinically meaningful ascites is defined as ascites from cirrhosis requiring diuretics or paracentesis<br>10. Known brain or leptomeningeal metastases<br>11. Known prior severe hypersensitivity to investigational product or any component in its formulations, including known severe hypersensitivity reactions to monoclonal antibodies (NCI CTCAE v5.0 Grade ≥ 3)<br>12. Other serious illness or medical conditions prior to study drug administration<br>a) Clinically significant (i.e., active) cardiovascular disease: cerebral vascular accident/stroke (< 6 months prior to enrollment), myocardial

	infarction (< 6 months prior to enrollment), unstable angina, congestive heart failure (≥ New York Heart Association Classification Class II), or serious cardiac arrhythmia requiring medication<br>b) Uncontrolled or poorly controlled hypertension despite optimal medical therapy<br>c) Current history of chronic diarrhea<br>d) Active disseminated intravascular coagulation<br>e) History of gastrointestinal perforation, fistulae or any clinically relevant arterial thromboembolic event within 6 months<br>f) Active infection that, in the opinion of the investigator, may increase the risk associated with study participation, study drug administration, or would impair the ability of the subject to receive study drug<br>g) Hepatitis B virus (HBV) or hepatitis C virus (HCV) infection at screening (positive HBV surface antigen or HCV RNA if anti-HCV antibody screening test positive)<br>h) Active autoimmune disease that might deteriorate when receiving an immuno-stimulatory agent. Patients with diabetes type I, vitiligo, psoriasis, or hypo- or hyperthyroid diseases not requiring immunosuppressive treatment are eligible.<br>i) Serious or non-healing wound, ulcer, or bone fracture within 28 days prior to first dose of protocol therapy<br>j) Prior organ transplantation including allogenic stem-cell transplantation<br>k) Other severe acute or chronic medical conditions including immune colitis, inflammatory bowel disease, immune pneumonitis, pulmonary fibrosis or psychiatric conditions including recent (within the past year) or active suicidal ideation or behavior; or laboratory abnormalities that may increase the risk associated with study participation or study treatment administration or may interfere with the interpretation of study results and, in the judgment of the investigator, would make the patient inappropriate for entry into this study<br>13. Current use of immunosuppressive medication, EXCEPT for the following:<br>a) intranasal, inhaled, topical steroids, or local steroid injection (e.g., intra-articular injection);<br>b) steroids as premedication for hypersensitivity reactions (e.g., CT scan premedication<br>c) short term steroids to prevent chemotherapy induced nausea

	14. The patient is receiving chronic antiplatelet therapy, including aspirin, nonsteroidal anti-inflammatory drugs (NSAIDs, including ibuprofen, naproxen, and others), dipyridamole or clopidogrel, or similar agents. Once-daily aspirin use (maximum dose 325 mg/day) is permitted<br>15. Vaccination within 4 weeks of the first dose of avelumab and while on trial is prohibited except for administration of inactivated vaccines<br>16. Subjects with interstitial lung disease that is symptomatic or may interfere with the detection or management of suspected drug-related pulmonary toxicity<br>17. Concurrent treatment with other experimental drugs or participation in another clinical trial with any investigational drug within 30 days but at least 5 half-lives of the investigational drug prior to treatment start<br>18. Known drug abuse/ alcohol abuse<br>19. Persisting toxicity related to prior therapy (NCI CTCAE v. 5.0 Grade > 1); however, alopecia, sensory neuropathy Grade ≤ 2, or other Grade ≤ 2 not constituting a safety risk based on investigator's judgment are acceptable<br>20. Subject pregnant or breast feeding, or planning to become pregnant within 3 months after the end of treatment<br>21. Subject (male or female) is not willing to use highly effective methods of contraception (per institutional standard) during treatment and for 30 days (male or female) after the end of treatment with avelumab and 3 months after the end of treatment with ramucirumab.<br>22. Patients known to have a HER2 positive cancer who have not been treated already with a HER2 targeting agent<br>23. Patients with a psychiatric illness or patients imprisoned or working in the Institution of the treating physician.
**Treatment, Dosage and Administration**	All eligible patients will receive avelumab and ramucirumab and paclitaxel until disease progression (according to RECIST v1.1), intolerable toxicity, withdrawal of consent or at a maximal treatment of 1 year. Thereafter treatment may continue outside of this trial.<br><br>*Avelumab* at a dose of 10mg/kg i.v. over 60 to 90 min, d1, d15, q28<br><br>*Ramucirumab* at dose of 8mg/kg i.v. over 60 minutes, d1, d15, q28<br><br>*Paclitaxel* at a dose of 80mg/m2 i.v. over 60 minutes, d1, d8 and d15, q28
**Assessments**	**Baseline assessments**<br><br>Consenting patients will have the following screening/baseline assessments performed within 4 weeks prior to the first treatment:<br><br>• Review of inclusion and exclusion criteria

	• Medical and medication history, physical examination including height, weight, vital signs (blood pressure, heart rate, respiratory rate, body temperature), ECOG-performance status<br>• 12-lead ECG<br>• Laboratory Tests (within 14 days before start of treatment)<br>o Hematology panel: hemoglobin, hematocrit, RBC count, platelet count, WBC count with differential count with neutrophils, lymphocytes, monocytes<br>o Chemistry panel: sodium, potassium, calcium, magnesium, phosphorus, chloride, serum creatinine, alkaline phosphatase, AST, ALT, total bilirubin, GGT, uric acid, total urea, total protein, albumin, amylase, lipase, creatine kinase, CRP, Glucose, LDH, cholesterol, triglyceride<br>o Coagulation panel: INR, aPTT<br>o Free T3 and T4 and TSH<br>o Serum tumor markers: CEA, CA 19-9, CA 72-4<br>o Urine dipstick (if protein ++, 24-hour urine analysis)<br>o Screening for hepatits: HBV surface antigen and anti-HCV; HCV RNA if anti-HCV antibody screening test is positive<br>o Serum or urin pregnancy test (beta-HCG) for women of childbearing potential (within 7 days before start of treatment)<br>o Blood draw (20ml) for translational research (2 Streck® tubes with 10ml each)+ 9ml EDTA tube<br>o Obtain paraffin-embedded tumor-tissue for translational research<br>• Tumor assessment by radiological imaging by CT chest and abdomen+ pelvis with iv contrast or CT chest + MRI abdomen + pelvis with iv contrast<br>The investigator will confirm the patient's eligibility after all baseline scans and laboratory results have been reviewed.<br>**Assessment during study**<br>Clinical assessment and blood samples will be taken once a week (break on day 21). Imaging with CT chest and abdomen+ pelvis with iv contrast or CT chest + MRI abdomen + pelvis with iv contrast and serum tumor markers will be done every 8 weeks.<br>**Assessment at day 1 each cycle (-2/+3 days thereafter)**

	The baseline assessments may be used at cycle 1, day 1 if within 7 days therapy of cycle 1 is started.<br>• Physical examination including height, weight, vital signs (blood pressure, heart rate, respiratory rate, body temperature), ECOG-performance status, assessment of toxicity, concomitant medication<br>• Laboratory tests<br>o Hematology panel: hemoglobin, hematocrit, RBC count, platelet count, WBC count with differential count with neutrophils, lymphocytes, monocytes<br>o Chemistry panel: sodium, potassium, calcium, magnesium, phosphorus, chloride, serum creatinine, alkaline phosphatase, AST, ALT, total bilirubin, GGT, uric acid, total urea, total protein, albumin, amylase, lipase, creatine kinase, CRP, Glucose, LDH<br>o Coagulation panel: INR, aPTT<br>o Free T3 and T4 and TSH<br>o Serum or urin pregnancy test (beta-HCG) for women of childbearing potential (within 7 days prior to start of the treatment) and every month Q4W while on treatment with study drug.<br>o Blood draw (20ml) for translational research (2 Streck® tubes) + 9ml EDTA tube only cycle 3, day 1<br>**Assessment at day 8 each cycle (-2/+3 days thereafter)**<br>• Physical examination including height, weight, vital signs (blood pressure, heart rate, respiratory rate, body temperature), ECOG-performance status, assessment of toxicity, concomitant medication<br>• Laboratory tests<br>o Hematology panel: hemoglobin, hematocrit, RBC count, platelet count, WBC count with differential count with neutrophils, lymphocytes, monocytes<br>**Assessment at day 15 each cycle (-2/+3 days thereafter)**<br>• Physical examination including height, weight, vital signs (blood pressure, heart rate, respiratory rate, body temperature), ECOG-performance status, assessment of toxicity, concomitant medication<br>• Laboratory tests<br>o Hematology panel: hemoglobin, hematocrit, RBC count, platelet count, WBC count with differential count with neutrophils, lymphocytes, monocytes

	o Chemistry panel: sodium, potassium, calcium, magnesium, phosphorus, serum creatinine, alkaline phosphatase, AST, ALT, total bilirubin<br>o Blood draw for translational research 20ml in 2 Streck® tubes + 9ml EDTA tube (only cycle 1)<br>**Tumor response assessment (every 8 weeks (±7 days))**<br>Tumor response evaluation has to be conducted every 8 weeks and is independent of actual therapy. Thus, even if study therapy is interrupted or delayed, tumor response assessment has to be done every 8 weeks (±7 days).<br>• Laboratory tests<br>o Serum tumor markers: CEA, CA 19-9, CA 72-4<br>• Disease assessment by radiological imaging with CT chest and abdomen+ pelvis with iv contrast or CT chest + MRI abdomen + pelvis with iv contrast<br>During treatment tumor response will be assessed by the investigator according to RECIST v1.1. All treatment decisions are based on the investigators assessment of response. A central independent radiology assessment reevaluating all scans will be performed after the end of treatment. Tumor assessment continues for one regular assessment after the local investigator identified progressive disease. The last tumor assessment is 8 weeks after identification of PD by local investigators. This is necessary in case the independent review disagrees with PD. In case treatment is discontinued for reasons other than PD tumor assessment continues up to 12 months after last treatment (or 2 months after PD).<br>**Final staging (end of treatment)**<br>The following assessments will be made if patient discontinues treatment:<br>• Physical examination including height, weight, vital signs (blood pressure, heart rate, respiratory rate, body temperature), ECOG-performance status, assessment of toxicity, concomitant medication<br>• Laboratory tests<br>o Hematology panel: hemoglobin, platelets, WBC with neutrophils, lymphocytes, monocytes<br>o Chemistry panel: sodium, potassium, calcium, magnesium, phosphorus, chloride, serum creatinine, alkaline phosphatase, AST, ALT, total bilirubin, GGT, uric acid, total urea, total protein, albumin, amylase, lipase, creatine kinase, CRP, Glucose, LDH, cholesterol, triglyceride

	o Coagulation: INR, aPTT<br>o Free T3 and free T4 and TSH<br>o Serum tumor markers: CEA, CA 19-9, CA 72-4<br>o Urine dipstick (if protein ++, 24-hour urine analysis)<br>o Serum or urin pregnancy test (beta-HCG) for women of childbearing potential<br>o Blood draw (20ml in 2 Streck® tubes) + 9ml EDTA tube for translational research<br>• Disease assessment by radiological imaging with CT chest and abdomen+ pelvis with iv contrast or CT chest + MRI abdomen + pelvis with iv contrast – unless disease progression seen in regular radiological imaging is the reason for disruption of therapy. In case of tumor progression, tumor assessment continues for one regular assessment after the local investigator identified PD. This means 8 weeks after identification of PD by local investigators another CT scan has to be performed.<br>**30 days safety follow-up (±7 days)**<br>• Physical examination, vital signs, performance status (ECOG), assessment of toxicity, concomitant medication<br>• Laboratory tests (hematology and chemistry panel), including free T3 and free T4 and TSH (if not done in the last 8 weeks)<br>**Extended safety follow-up**<br>Given the potential risk for delayed immune-related toxicities, safety follow-up must be performed every 30 days (±7 days) up to 90 days after the last dose of avelumab administration.<br>The first follow up 30 days after the last dose of avelumab is obligatory a site visit. The extended safety follow-up beyond 30 days after last study drug administration should also be performed via a site visit. Only if the patient is unable to come to the study center it can be done via telephone call.<br>**Follow up**<br>All subjects will be followed every 3 months ± 28 days for up to two years after start of treatment.<br>In case of progressive disease after study treatment only:<br>• survival, disease status, protracted toxicity, further treatment

	• One further tumor assessment by radiological imaging 8 weeks after the local investigator identified PD.<br><br>In any other case additionally:<br><br>• disease assessment (by radiological imaging via CT/MRI scan as listed above), physical examination including weight, vital signs, ECOG-performance status
**Translational Research**	The following translational research is currently planned, but may be adapted taking into account new research data<br><br>• Tumor-infiltrating lymphocytes (TiL) repertoire determination from tumor and liquid biopsy<br><br>• Liquid biopsy next-generation sequencing (NGS) immunoprofiling (TCRβ & IgH) before treatment initiation and before second avelumab dose to determine response predictive immune signature (diversification pattern as read-out for ongoing immune activation, TiL clone expansion in peripheral blood)<br><br>• Correlation of quantitative ctDNA and clonal dynamics with immune response signature to determine control of mutant subclones.<br><br>• FACS analysis of T-cell subpopulations<br><br>• In addition FFPE will be stained for PD-L1 (DAKO), analysed for microsatellite instability (MSI), Epstein Barr Virus association (EBV) and correlated with clinical efficacy.<br><br>Thus, the tumor block or alternatively 10 slides for TiL analysis and PD-L1 staining will be obtained at baseline. Prior to first treatment, d15 cycle 1, d1 cycle 3 and end of treatment or progression blood will be obtained and directly shipped. 2 10ml Streck® tubes will be used for blood sampling. 1x 9ml EDTA tube will be used for FACS analysis.
**Statistics**	The present trial is designed as a single arm phase II study, which aims to estimate the therapeutic efficacy of the experimental regimen.<br><br>The efficacy assumptions are derived from historical data.<br><br>The primary endpoint of the study is the overall survival rate at 6 months based on the ITT population.<br><br>Paclitaxel/ramucirumab could achieve an OS rate at 6 months of 65% in the Western population of the Rainbow trial. Although we expect worse prognostic parameters in our study population, we hope to achieve an OS rate of 65% at 6 months by combining paclitaxel / ramucirumab with avelumab.

	• The experimental therapy would be considered to be a highly promising candidate for further development (e.g. in a phase III trial), if the true OS rate amounted to 65% or more.<br>• On the other hand, the experimental therapy would be rated as insufficiently active, if the true OS rate is 50% or lower, as this suggests a distinct inferiority to paclitaxel / ramucirumab.<br>• Probability to accept the experimental therapy as promising (≥ 65% OS rate) with respect to efficacy, in spite of a true OS rate of ≤ 50%: 0.10 (type I error)<br>• Probability to reject the experimental therapy as not sufficiently efficient (≤ 50%), although the true OS rate is promising (≥ 65%): 0.2 (type II error, corresponding to a power of 80%).<br>To allow the option of earlier stopping for futility in case of unfavorable results, a standard two-stage phase II design according to Simon (1989) is applied. In the first stage, n = 33 patients with the endpoint (OS status at 6 months) available are analyzed, and the trial is stopped if the number of „successes" is only 16 or lower. Otherwise, the study is continued until a total of 53 patients evaluable for efficacy, (as defined in section 13.2, ITT population) have been recruited.<br>The final conclusion of the phase II trial will depend on the definite OS rate (and its confidence interval), as well as the information on type, frequency and severity of toxicities. Formally, more than 31 patients alive at 6 months are required to reject the null hypothesis defined above. Assuming a 10% drop out rate we are planning to include 59 pts.
**Independent Data Monitoring Committee (IDMC)**	The independent data monitoring committee will be informed about all SAE and discuss the safety findings and the development of the trial from the time of the first patient in at 3 months, at 6 months and every 6 months thereafter. This way a continuous safety monitoring is available. In case of any unexpectedly high toxicity a meeting of the IDMC will be arranged.<br>**Interim analysis**<br>Interim analysis for futility: see above. The IDMC will discuss the results with the sponsor and decide together with the sponsor about continuation of the trial.

4 INTRODUCTION AND BACKROUND

4.1 Background

Second-line chemotherapy prolongs survival in metastatic gastro-esophageal cancer compared to best supportive care. A randomised phase III trial from the Arbeitsgemeinschaft Internistische Onkologie (AIO) was the first trial to prove this survival benefit (Thuss-Patience et al. 2011). The positive effect on overall survival and quality of life could be confirmed in two larger subsequent trials (J. H. Kang et al. 2012; Ford et al. 2014). Irinotecan showed similar efficacy to paclitaxel in the second line setting (Hironaka et al. 2013).

Ramucirumab, a VEGF Receptor 2 antibody has been investigated in two randomized phase III trials in chemorefractory gastric cancer in the second-line setting (REGARD-trial (ramucirumab vs BSC; (Fuchs et al. 2014) and RAINBOW-trial (Wilke et al. 2014; Shitara et al. 2016)). In the RAINBOW trial ramucirumab + paclitaxel was compared to placebo + paclitaxel and showed an improvement of response rate and overall survival. This trial lead to the registration of ramucirumab in combination with paclitaxel, which is now the preferred standard treatment option in second line therapy.

Due to these data and the current best investigated standard treatment as second line in gastro-esophageal cancer is paclitaxel + ramucirumab.

Currently PD-1 and PD-L1 inhibitors are a very promising treatment option in gastro-esophageal adenocarcinoma which are investigated in a number of different trials. In patients who are responding to PD-1 blockade astonishingly long lasting responses could be detected (Muro et al. 2016; Chung et al. 2016). In a recently presented randomized phase III trial 493 patients with gastric cancer who were pretreated with at least 2 lines of prior palliative chemotherapy regimens received either nivolumab 3mg/kg or placebo. A clinically highly relevant and statistically significant prolongation of survival could be shown (HR 0.63; $p<0,0001$) and the rate of survival at 12 months was increased from 10.9% to 26.6% (Y.-K. Kang et al. 2017). In Caucasians similar efficacy of nivolumab can be expected and could be shown in a phase I/II trial (Janjigian et al. 2016). Approval of nivolumab as salvage treatment after available standard therapy can be expected.

Chung et al. (Chung et al. 2016) reported promising activity of avelumab monotherapy as maintenance or in second line in advanced gastric cancer patients in a phase Ib trial. Javelin 100 (NCT02625610) investigates in a randomized phase III the value of a maintenance therapy with avelumab after 1st-line FOLFOX therapy, with pending results. In contrast the Javelin 300 (NCT02625623) 3rd line phase III trial investigating avelumab monotherapy compared to paclitaxel or irinotecan has reported no survival benefit in a recent press release. Recently the results of Keynote 061 have also been reported: Pembrolizumab did not significantly improve overall survival compared with paclitaxel as second-line therapy for advanced gastric or gastro-oesophageal junction cancer with PD-L1 CPS of 1 or higher.(Shitara et al. 2018) These disappointing results emphasize the great need for trials investigating a combination therapy of checkpoint inhibition and chemotherapy to increase the proportion of patients who benefit from the novel immunotherapy (Smyth and Thuss-Patience 2018).

4.2 Medical need for this trial

Up to now the proportion of patients with gastro-esophageal cancer responding to PD-1 blockade is relatively small, with response rates in the range of 10-20% (Fuchs et al. 2017). There are no established biomarkers for response prediction. PD-L1 expression, EBV status, microsatellite instability, mutational load and a y-interferone gene signature are interesting candidates for response prediction, but none is established yet.

A very promising approach to enlarge the proportion of benefitting patients is the combination of chemotherapy with PD-1 blockade because of a chemotherapy induced increase of immunogenicity of tumor cells (Kroemer et al. 2013). Currently this approach is investigated in the 1st line setting with pembrolizumab in combination with chemotherapy (platin / 5-FU) (Keynote 062 trial) Results of a safety and efficacy cohort (Keynote-059) were recently presented and are very promising. Tolerabilty was good and efficacy promisingly increased (Bang et al. 2017).

Paclitaxel has also been investigated in combination with PD-1 antibodies. As neoadjuvant treatment paclitaxel + pembrolizumab was well tolerated and promising (Nanda et al. 2017).

Furthermore a synergistic effect of antiangiogenic treatment with checkpoint-inhibition could be shown (Lieu et al. 2014; Hodi et al. 2014; Chau, Bendell, Calvo, Santana-Davila, Arkenau, et al. 2017). In a phase I study ramucirumab was combined with pembrolizumab in treatment naïve and previously treated gastro-esophageal adenocarcinoma. In the

second line cohort 57% of patients experienced a decrease in target lesions size (Chau, Bendell, Calvo, Santana-Davila, Arkenau, et al. 2017).

Currently there are no treatment options incorporating PD-1 blockade in the second line setting in gastro-esophageal adenocarcinomas. For the randomized phase III Keynote 061 trial investigating pembrolizumab versus paclitaxel no survival benefit was reported in a recent press release. To improve survival with a monotherapy of a targeted agent compared to paclitaxel is difficult, which was recently shown by an international phase III trial comparing T-DM1 versus paclitaxel, which failed to show superiority in the second line setting (Thuss-Patience et al. 2017). Furthermore, the actual standard second line regimen is paclitaxel + ramucirumab not single agent paclitaxel.
Even if nivolumab may be available as salvage treatment for gastric cancer in the future its value as second line is unclear as active chemotherapies for this setting are available. Therefore, a combination of immune checkpoint blockade and chemotherapy is warranted, particularly in regard of recent positive data in lung cancer (Keynote 189) and promising data in 1st line gastric cancer (Keynote 059, cohort 2).

Due to these reasons a combination of PD-L1 inhibition with the best established second line chemotherapy (paclitaxel+ramucirumab) is the logical next step to improve survival of metastatic gastric cancer patients and to establish PD-L1 blockade in the second line setting in combination with the currently available best second line regimen.

4.3 Expected tolerability

So far there are no unexpected or overlapping toxicities reported in the trials combining PD-1 blockade and chemotherapy. Pembrolizumab + paclitaxel was well tolerated in 69 patient with breast cancer (Nanda et al. 2017).
The combination of ramucirumab and pembrolizumab was investigated in 41 previously treated patients with gastric cancer. The safety profile of ramucirumab combined with pembrolizumab was consistent with monotherapy treatment of each drug with no additive toxicities (Chau, Bendell, Calvo, Santana-Davila, Rodon Ahnert, et al. 2017).
Also the combination of chemotherapy, anti-angiogenesis and immunotherapy was investigated: Bendell et al. examined the combination of MPDL3280A (anti-PDL-1) + bevacizumab with or without FOLFOX in patients with colorectal cancer. MPDL3280A + bevacizumab with or without FOLFOX was well tolerated with no unexpected toxicities

in this trial. Clinical activity was observed with both treatment combinations. (Bendell et al. 2015)

5 STUDY OBJECTIVE

The primary clinical objective is to determine the efficacy of a standard second-line regimen (paclitaxel + ramucirumab) with avelumab in patients with metastatic gastro-oesophageal cancer in terms of overall survival rate (OSR) at 6 months (according to RECIST v1.1).

The main secondary objective is to determine safety and tolerability, according to NCI CTC AE v5.0 and to the obtained data on vital signs, clinical parameters and feasibility of the regimen. Further secondary objectives are to determine the efficacy of the therapy in terms of objective response rate (acc. to RECIST v1.1) including the duration of response, overall survival (OS), OSR at 12 month, progression free survival (PFS) and progression free survival rate (PFSR) at 6 months and at 12 months.

For efficacy parameters (PFS and ORR) an exploratory analysis according to modified RECIST will be performed.

The primary translational objective is the determination of an efficacy predictive immune signature (diversification pattern and TiL clone expansion in peripheral blood) and the subgroup analyses of all primary and secondary endpoints in view of PD-L1 status.

6 STUDY DESIGN

This is a single arm, multicenter phase II trial designed to assess the clinical performance of avelumab in combination with paclitaxel and ramucirumab as second-line treatment in patients with gastric or gastro-oesohageal junction adenocarcinoma.

6.1 Primary endpoint

- Overall Survival Rate (OSR) at 6 months

6.2 Secondary endpoints

- Overall Survival
- OSR at 12 month
- Progression Free Survival
- Progression Free Survival Rate at 6 and 12 month according to RECIST v1.1
- Safety and tolerability (acc. to NCI CTC AE v5.0 and to the obtained data on vital signs, clinical parameters and feasibility of the regimen)
- Best response according to RECIST v1.1
- Confirmed response rate according to RECIST v1.1
- Duration of response
- Translational research (correlation of efficacy parameters with immunoprofiling (TCRβ & IgH); tumor-infiltrating lymphocytes (TiL) repertoire determination, quantitative ctDNA and clonal dynamics)
- Subgroup analyses of all primary and secondary endpoints in view of PD-L1 status
- Efficacy parameters (PFS and ORR) according to modified RECIST

7 STUDY POPULATION

7.1 Number of patients

In this trial N=59 patients diagnosed with gastric or gastro-esophageal junction adenocarcinoma, with proven clinical/imaging tumor progression after 1st line chemotherapy will be enrolled (provided that the interim analysis does not advise for early closure). In the number of 59 patients 10% drop out rate is included (see statistics), 53 evaluable patients are needed.

7.2 Selection criteria

7.2.1 Inclusion criteria

1. Signed written informed consent
2. Male or female ≥ 18 years of age
3. Histologically proven gastric adenocarcinoma including adenocarcinoma of the esophagogastric junction
4. Metastatic or locally advanced disease, not amenable to potentially curative resection
5. Documented objective radiological or clinical disease progression during or within 6 months of the last dose of first-line platinum and fluoropyrimidine doublet with or without anthracycline, docetaxel or trastuzumab. Neoadjuvant/adjuvant treatment is not counted unless progression occurs <6 months after completion of the treatment. In these cases neoadjuvant/adjuvant treatment is counted as first line.
6. Measurable or non-measurable but evaluable disease determined using guidelines RECIST 1.1
7. ECOG performance status 0-1
8. Life expectancy > 12 weeks
9. Adequate hematological, hepatic and renal functions:
   a) Absolute neutrophil count (ANC) ≥ 1.5 x 10^9/L
   b) Platelet count ≥ 100 x 109/L
   c) Hemoglobin ≥ 9 g/dl (may have been transfused)

d) Total bilirubin ≤ 1.5 times the upper limit of normal (ULN) and AST and ALT ≤ 2.5 x ULN in absence of liver metastases, or ≤ 5 x ULN in presence of liver metastases; AP ≤ 5 x ULN
e) Estimated creatinine clearance ≥ 30 mL/min according to the Cockcroft-Gault formula (or local institutional standard method)
f) Urinary protein ≤ 1+ on dipstick or routine urinalysis (UA; if urinedipstick or routine analysis is ≥ 2+, a 24-hour urine collection for protein must demonstrate < 1000 mg of protein in 24 hours to allow participation in this protocol)
g) Adequate coagulation function as defined by International Normalized Ratio (INR) ≤ 1,5 ULN, and a partial thromboplastin time (PTT) ≤ 5 seconds above the ULN (unless receiving anticoagulation therapy). Patients receiving warfarin/phenprocomon must be switched to low molecular weight heparin and have achieved stable coagulation profile prior to first dose of protocol therapy.

10. Women of child-bearing potential must have a negative urine or serum pregnancy test
11. Highly effective contraception for both male and female subjects throughout the study and for at least 30 days after last avelumab and at least 3 months after last ramucirumab treatment administration if the risk of conception exists
12. Ability to comply with scheduled assessments and with management of toxicities.

7.2.2 Exclusion criteria

Patients with any of the following will not be eligible for participation:

1. Other tumor type than adenocarcinoma (e.g. leiomyosarcoma, lymphoma) or a second cancer except in patients with squamous or basal cell carcinoma of the skin or carcinoma in situ of the cervix that has been effectively treated. Patients curatively treated for any other malignancy and disease-free for at least 5 years will be discussed with the sponsor before inclusion
2. Concurrent chronic systemic immune therapy, chemotherapy, or hormone therapy not indicated in the study protocol
3. Previous therapy with, paclitaxel or ramucirumab or pretreatment with a PD-1, PD-L1 inhibitor
4. Current treatment with any anti-cancer therapy ≤ 2 weeks prior to study treatment start unless rapidly progressing disease is measured

5. Previous exposure to a VEGF or VEGFR inhibitor or any antiangiogenic agent, or prior enrolment in this study
6. Major surgical procedure, open biopsy or significant traumatic injury within 4 weeks prior to start of study treatment; anticipation of need for major surgical procedure (e.g. impending bowel obstruction) during the course of the study
7. Grade 3-4 GI bleeding within 3 months prior to enrollment
8. History of deep vein thrombosis (DVT), pulmonary embolism (PE), or any other significant thromboembolism (venous port or catheter thrombosis or superficial venous thrombosis are not considered “significant”) during the 3 months prior to first dose of protocol therapy
9. Cirrhosis at a level of Child-Pugh B (or worse) or cirrhosis (any degree) and a history of hepatic encephalopathy or clinically meaningful ascites resulting from cirrhosis. Clinically meaningful ascites is defined as ascites from cirrhosis requiring diuretics or paracentesis
10. Known brain or leptomeningeal metastases
11. Known prior severe hypersensitivity to investigational product or any component in its formulations, including known severe hypersensitivity reactions to monoclonal antibodies (NCI CTCAE v5.0 Grade ≥ 3)
12. Other serious illness or medical conditions prior to study drug administration
    a) Clinically significant (i.e., active) cardiovascular disease: cerebral vascular accident/stroke (< 6 months prior to enrollment), myocardial infarction (< 6 months prior to enrollment), unstable angina, congestive heart failure (≥ New York Heart Association Classification Class II), or serious cardiac arrhythmia requiring medication
    b) Uncontrolled or poorly controlled hypertension despite optimal medical therapy
    c) Current history of chronic diarrhea
    d) Active disseminated intravascular coagulation
    e) History of gastrointestinal perforation, fistulae or any clinically relevant arterial thromboembolic event within 6 months
    f) Active infection that, in the opinion of the investigator, may increase the risk associated with study participation, study drug administration, or would impair the ability of the subject to receive study drug

g) Hepatitis B virus (HBV) or hepatitis C virus (HCV) infection at screening (positive HBV surface antigen or HCV RNA if anti-HCV antibody screening test positive)
h) Active autoimmune disease that might deteriorate when receiving an immuno-stimulatory agent. Patients with diabetes type I, vitiligo, psoriasis, or hypo- or hyperthyroid diseases not requiring immunosuppressive treatment are eligible.
i) Serious or non-healing wound, ulcer, or bone fracture within 28 days prior to first dose of protocol therapy
j) Prior organ transplantation including allogenic stem-cell transplantation
k) Other severe acute or chronic medical conditions including immune colitis, inflammatory bowel disease, immune pneumonitis, pulmonary fibrosis or psychiatric conditions including recent (within the past year) or active suicidal ideation or behavior; or laboratory abnormalities that may increase the risk associated with study participation or study treatment administration or may interfere with the interpretation of study results and, in the judgment of the investigator, would make the patient inappropriate for entry into this study

13. Current use of immunosuppressive medication, EXCEPT for the following:
    a) intranasal, inhaled, topical steroids, or local steroid injection (e.g., intra-articular injection);
    b) steroids as premedication for hypersensitivity reactions (e.g., CT scan premedication
    c) short term steroids to prevent chemotherapy induced nausea
14. The patient is receiving chronic antiplatelet therapy, including aspirin, nonsteroidal anti-inflammatory drugs (NSAIDs, including ibuprofen, naproxen, and others), dipyridamole or clopidogrel, or similar agents. Once-daily aspirin use (maximum dose 325 mg/day) is permitted
15. Vaccination within 4 weeks of the first dose of avelumab and while on trial is prohibited except for administration of inactivated vaccines
16. Subjects with interstitial lung disease that is symptomatic or may interfere with the detection or management of suspected drug-related pulmonary toxicity
17. Concurrent treatment with other experimental drugs or participation in another clinical trial with any investigational drug within 30days but at least 5 half-lives of the investigational drug prior to treatment start
18. Known drug abuse/ alcohol abuse

19. Persisting toxicity related to prior therapy (NCI CTCAE v. 5.0 Grade > 1); however, alopecia, sensory neuropathy Grade ≤ 2, or other Grade ≤ 2 not constituting a safety risk based on investigator's judgment are acceptable
20. Subject pregnant or breast feeding, or planning to become pregnant within 3 months after the end of treatment
21. Subject (male or female) is not willing to use highly effective methods of contraception (per institutional standard) during treatment and for 30 days (male or female) after the end of treatment with avelumab and at least 3 months after the end of treatment with ramucirumab.
22. Patients known to have a HER2 positive cancer who have not been treated already with a HER2 targeting agent
23. Patients with a psychiatric illness or patients imprisoned or working in the Institution of the treating physician.

8 STUDY PROCEDURES AND METHODOLOGY

8.1 Study schedule overview

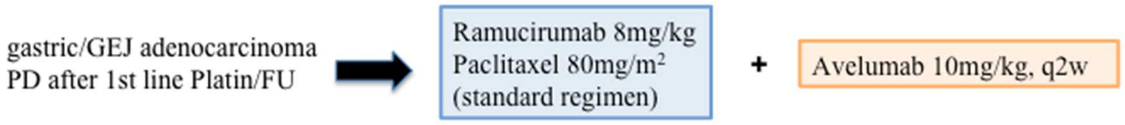

Figure 1 Study schedule overview

8.2 Treatment

Avelumab 10 mg/kg	d1, d15, q28
followed by Ramucirumab 8 mg/kg	d1, d15, q28
followed by Paclitaxel 80 mg/m²	d1, d8, d15, q28

Avelumab is not a standard of care for metastatic gastric cancer. Thus, avelumab will be supplied by Merck and defined as an investigational medicinal product (IMP). Merck will deliver avelumab to the local pharmacy for preparation and administration. The relevant information on the drug characteristics, storage, application, mode of action and adverse reactions is included in the Investigational Brochure (IB) and the Summary of Product Characteristics (SmPC, Fachinformation).

8.2.1 Dosing and schedule

The dose of the therapy is dependent upon the patient's baseline body weight in kilograms and the body surface. The first dose of treatment is dependent upon the patient's baseline body weight in kilograms and height. Patients should be weighed at the beginning of each cycle and may be dosed based on actual body weight. If there is a ≥10% change (increase or decrease) in body weight from the last dose calculation, then the dose of treatment must be recalculated. For patients undergoing repeated palliative drainage procedures to remove pleural or peritoneal fluid, dry weight will be defined as weight obtained after the drainage procedure and before fluid reaccumulation. In such circumstances, dry weight

will be used for dose calculation, if obtained ≤30 days prior to dose. If no recent dry weight is available, actual weight will be used.
Therapy has to be administered in the following order:

1. **Avelumab** at a dose of 10 mg/kg will be given by i.v. infusion over 60 to 90 min on day 1 and 15 of a 28-day cycle.
   Please refer to respective SmPC and IB for further details for administration of avelumab.
2. **Ramucirumab** at dose of 8 mg/kg will be given by i.v. infusion over 60 minutes on day 1 and 15 of a 28-day cycle.
   Please refer to the respective SmPC for further details for administration of ramucirumab.
3. **Paclitaxel** at a dose of 80 mg/m2 will be given by i.v. infusion over 60 minutes on day 1, 8 and 15 of a 28-day cycle.
   Premedication to avoid severe hypersensitivity reactions should be given. Please refer to the respective SmPC for further details for administration of paclitaxel.

8.2.2 Premedication

In order to mitigate infusion-related reactions, a premedication with

- antihistamine (for example 4 mg dimetindenmaleat)
- H2 blocker (for example 50mg ranitidin)
- 500 mg paracetamol i.v. or oral
- 10 mg dexamethason

before treatment infusions is recommended.
Premedication 30 to 60 minutes prior to the first 4 infusions of avelumab is mandatory. Premedication for subsequent avelumab infusions should be administered based upon clinical judgment and presence/severity of prior infusion reactions. This may be modified based on local treatment standards and guidelines, as appropriate. Also for ramucirumab a premedication with an antihistamine is recommended and after an infusion related toxicity a premedication is mandatory.
In the absence of any hypersensitivity reactions, and at the discretion of the treating physician, continuation of the use of dexamethasone is optional after the first 2 administrations of paclitaxel.

8.2.3 Special precautions for administration

Avelumab should be administered in a setting that allows for immediate access to an intensive care unit or equivalent environment and administration of therapy for anaphylaxis, such as the ability to implement immediate resuscitation measures. Steroids (dexamethasone 10 mg), epinephrine (1:1,000 dilution), allergy medications (IV antihistamines), bronchodilators, or equivalents, and oxygen should be available for immediate access. After adimistration of avelumab there must be a break of at least 30 minutes before therapy with ramucirumab is given.
Following avelumab infusions, patients must be observed for 30 minutes post-infusion for potential infusion-related reactions.

8.2.4 Treatment duration

Treatment with avelumab, paclitaxel and ramucirumab will be administered until progression (according to RECIST v1.1), intolerable toxicity, withdrawal of consent or at a maximal treatment of 1 year. Thereafter treatment may continue outside of this trial after discussion with the sponsor. (for details see section 8.6.2).

8.2.5 Study medication

The study medication (avelumab) will be supplied by Merck in vials of 200 mg and delivered to the local pharmacy for preparation and administration.
The relevant information on the drug characteristics, storage, application, mode of action and adverse reactions is included in the IB.
In addition, the SmPC for the used backbone drugs (paclitaxel, ramucirumab) will be supplied to ensure administration of drugs according to SmPC.

8.2.6 Concomitant medication

The investigator should instruct the patient to notify the study site about any new medications he/she takes after the start of the study drug. All medications (other than study drug) and significant non-drug therapies (including physical therapy and blood

transfusions) administered after the patient starts treatment with study drug must be listed in the eCRF.
Patients must be instructed not to take any additional medications (over-the-counter or other products) during the study without prior consultation with the investigator. All medications taken within 7 days of starting study treatment should be reported in the eCRF.

Permitted therapy

Radiation (palliative radiotherapy) of bone lesions during the study, if clinically indicated, can be considered after consultation with the sponsor, as long as no target lesion is irradiated.
Concomitant therapy includes any prescription medications or over-the-counter preparations used by a patient between the 7 days preceding the screening evaluation and the treatment discontinuation visit.
The use of inhaled corticosteroids for COPD and mineralocorticoids (e.g., fludrocortisone) is allowed.
Colony-stimulating factors, such as granulocyte colony-stimulating factor and erythropoietin, should only be used according to the ASCO and ASCO/ASH guidelines, respectively (Smith et al. 2006; Rizzo et al. 2010). Influenza vaccination should be given during influenza season only (approximately October to March). Patients must not receive live, attenuated influenza vaccine (e.g., FluMist®) within 4 weeks prior to first treatment or at any time during the study and for a period of 90 days after the discontinuation of avelumab but may receive inactivated vaccine.
Patients who use hormonal therapy like oral contraceptives, hormone-replacement therapy, prophylactic or therapeutic anticoagulation therapy (such as low molecular weight heparin or warfarin at a stable dose level) should continue their use.
Females of reproductive potential should use a highly effective means of contraception.
The use of analgesic agents during the conduct of the study is permitted at the discretion of the investigator. The chronic use of non-steroidal anti-inflammatory drugs (NSAIDs) with a high risk of bleeding (for example, indomethacin, ibuprofen, naproxen, or similar agents) is strongly discouraged unless at the discretion and responsibility of the investigator after careful assessment of the individual bleeding risk of the patient. Chronic use of analgesic agents with no or low bleeding risk (for example, paracetamol/acetaminophen, metamizole, dipyrone, or propyphenazone) is acceptable.

Patients experiencing febrile neutropenia, especially with diarrhea or dyspnea, should be managed in a hospital setting according to standard procedures, with the urgent initiation of intravenous antibiotic therapy.

Premedication should be given prior to administration of the study therapy as listed above. Additional premedication may be provided at investigator discretion. All premedication administered must be adequately documented in the eCRF. Patients should be premedicated with antihistamines, corticosteroids, paracetamol, etc., after experiencing a Grade 1 or 2 infusion-related reaction. If a Grade 3 or 4 infusion-related reaction occurs, patients should be treated with epinephrine, bronchodilators, and/or glucocorticoids for symptomatic bronchospasm and i.v. fluids and/or pressors for hypotension. For a second Grade 1 or 2 infusion-related reaction, administer dexamethasone 10 mg i.v. (or equivalent); then, for subsequent infusions, premedicate with dimetidin 4 mg i.v. (or equivalent), paracetamol 500 mg orally, and dexamethasone 10 mg I.V. (or equivalent).

Transfusions of red blood cells, platelets, or other blood products are permitted at the investigator's discretion during the conduct of the study.

All concomitant medications should be reported to the investigator and recorded on the appropriate eCRF.

Prohibited therapy

The concomitant use of herbal therapies is not recommended because their pharmacokinetics, safety profiles, and potential drug-drug interactions are generally unknown. However, their use for patients on study is allowed at the discretion of the investigator, provided that there are no known interactions with any study treatment. Herbal therapies intended for the treatment of cancer are prohibited.

Patients who are receiving a receptor activator of nuclear factor kappa B ligand inhibitor (**denosumab**) prior to enrolment must be willing and eligible to receive a bisphosphonate instead while on study; denosumab could potentially alter the activity and the safety of avelumab.

Patients should not receive any live, attenuated **vaccine** (e.g. FluMist®) at any time during the study while the patient is receiving avelumab and for a period of 90 days after the discontinuation of avelumab.

Patients are not allowed to receive immunostimulatory agents, including but not limited to interferon (IFN)-α, IFN-γ, or IL-2, during the entire study. These agents, in combination with avelumab, could potentially increase the risk for autoimmune

conditions. In addition, all patients should not receive other immunostimulatory agents for 10 weeks after the last dose of avelumab.

Patients should also not receive **immunosuppressive medications**, including but not limited to cyclophosphamide, azathioprine, methotrexate, and thalidomide. These agents could potentially alter the activity and the safety of avelumab. Systemic corticosteroids and anti−TNF-α agents may attenuate potential beneficial immunologic effects of treatment with avelumab, and should also not be administered (except as described in the exclusion criteria).

The above list of medications is not necessarily comprehensive. The investigator should consult the prescribing information for any concomitant medication and contact the Medical Monitor if questions arise regarding medications not listed above.

8.3 Assessment and guidelines for visits

Study Schedule Visit	Screening	During treatment			End of Treatment	Safety follow up	Follow up[15]
**Study week (wks)**	Baseline (within 4 wks prior to cycle 1)	d 1	d 8	d 15		After 30/60/90 days[7]	every 3 months
**Visit window**		+3/-2 days			±7 days	±7 days	± 28 days
**Informed consent**	X						
**Medical history, demographics**	X						
**Physical examination[1]**	X	X	X	X	X	X	
**Vital signs[2]**	X	X	X	X	X	X	
**ECG 12 lead**	X						
**Performance status (ECOG)**	X	X	X	X	X	X	X
**Concomitant medication**	X	X	X	X	X	X	
**Blood hematology panel[3]**	X	X	X	X	X	X	
**Blood chemistry panel large[4]**	X	X			X	X	
**Blood chemistry panel small[5]**				X			
**Blood coagulation[6]**	X	X			X	X	
**TSH, fT3, fT4**	X	X			X		
**Tumor markers (CEA, CA 19-9, CA 72-4)**	X	X[7]			X		X[13]
**Unrine analysis[8]**	X				X		
**HBV/HCV test[9]**	X						
**Pregnancy test[10]**	X	X			X		
**Blood draw translat. Research[11]**	X	X		X	X		
**Obtain tumor tissue**	X						
**Treatment[12]** – **Avelumab**		X		X			
**Treatment[12]** – **Ramucirumab**		X		X			
**Treatment[12]** – **Paclitaxel**		X	X	X			
**Tumor assessment (CT/MRI)[7]**	X	X			X		X[13]
**Further treatment ($3^{rd}/4^{th}$ line)**							X
**AE monitoring**	X	X	X	X	X	X[14]	X[14]
**Survival**	X	X	X	X	X	X	X

Table 1 Flow Chart

1: **Physical examination:** including weight, height (only baseline)
2: **Vital signs:** blood pressure, heart rate, respiratory rate, body temperature
3**: Hematology panel**: hemoglobin, hematocrit, RBC count, platelet count, WBC count with differential count with neutrophils, lymphocytes, monocytes
4: **Chemistry panel large**: sodium, potassium, calcium, magnesium, phosphorus, chloride, serum creatinine, alkaline phosphatase, AST, ALT, total bilirubin, GGT, uric acid, total urea, total protein, albumin, amylase, lipase, creatine kinase, CRP, Glucose, LDH
cholesterol, triglyceride only baseline and EOT

5: **Chemistry panel small**: sodium, potassium, calcium, magnesium, phosphorus, serum creatinine, alkaline phosphatase, AST, ALT, total bilirubin
6: **Coagulation panel**: INR, aPTT
7: **Tumor assessment:** every 8 weeks ±7 days by CT chest and abdomen+ pelvis with iv contrast or CT chest + MRI abdomen + pelvis with iv contrast and tumor markers. Assessment of tumor response continues until PD is identified by the local investigators and repeated once 8 weeks after identification of PD. If no PD is seen during study tumor assessment will be done every 3 months during FU until PD is identified by the local investigators and repeated once 8 weeks after identification of PD.
8: **Urine analysis:** dipstick, 24-hours urine analysis if protein ++
9: **Screening for hepatits:** HBV surface antigen and anti-HCV; HCV RNA if anti-HCV antibody screening test is positive
10: **Pregnancy test:** serum or urin pregnancy test (beta-HCG) in women of childbearing potential (at baseline within 7 days prior to start of treatment) and every month Q4W while on treatment with study drug.
11: **Translational project:** blood draw baseline , cycle 1 day 15, cycle 3 day 1 and EOT (20ml in 2 Streck® tubes + 9ml EDTA tube)
12: Treatment with avelumab, paclitaxel and ramucirumab will be administered until progression (according to RECIST v1.1), intolerable toxicity, withdrawal of consent or up to a maximal treatment of 1 year. Thereafter treatment may continue outside of this trial.
13: In case of no PD during or after study treatment, assessment of tumor response continues every 8 weeks until PD is identified by the local investigators and repeated once 8 weeks after identification of PD.
14: Immune related AEs and irSAEs that are in a reasonable causality (possible, probable, definitive) to Avelumab have to be reported up to 90 days after last treatment. Immune relates AEs and irSAEs have to be followed up until complete resolution..
15: **Follow up:** 30 days safety follow up should be on site, 60 and 90 days could be by phone if patient is not able to come to site. Follow up continues to a maximum of 1 year after last dose of treatment.

8.3.1 Baseline assessments

Consenting patients will have the following screening/baseline assessments performed within 4 weeks prior to the first treatment:

- Review of inclusion and exclusion criteria
- Medical and medication history, physical examination including height, weight, vital signs (blood pressure, heart rate, respiratory rate, body temperature), ECOG-performance status
- 12-lead ECG
- Laboratory Tests (within 14 days before start of treatment)
  - Hematology panel: hemoglobin, hematocrit, RBC count, platelet count, WBC count with differential count with neutrophils, lymphocytes, monocytes
  - Chemistry panel: sodium, potassium, calcium, magnesium, phosphorus, chloride, serum creatinine, alkaline phosphatase, AST, ALT, total bilirubin, GGT, uric acid, total urea, total protein, albumin, amylase, lipase, creatine kinase, CRP, Glucose, LDH, cholesterol, triglyceride
  - Coagulation panel: INR, aPTT
  - Free T3 and T4 and TSH
  - Serum tumor markers: CEA, CA 19-9, CA 72-4

    - Urine dipstick (if protein ++, 24-hour urine analysis)
    - Screening for hepatits: HBV surface antigen and anti-HCV; HCV RNA if anti-HCV antibody screening test is positive
    - Serum or urin pregnancy test (beta-HCG) for women of childbearing potential (within 7 days before start of treatment)
    - Blood draw (20ml) for translational research (2 Streck® tubes with 10ml each) + 9 ml in 1 EDTA tube
    - Obtain paraffin-embedded tumor-tissue for translational research
- Tumor assessment by radiological imaging by CT chest and abdomen+ pelvis with iv contrast or CT chest + MRI abdomen + pelvis with iv contrast

The investigator will confirm the patient's eligibility after all baseline scans and laboratory results have been reviewed.

8.3.2 Assessment during study

Clinical assessment and blood samples will be taken once a week (break on day 21). Imaging with CT chest and abdomen+ pelvis with iv contrast or CT chest + MRI abdomen + pelvis with iv contrast and serum tumor markers will be done every 8 weeks.

Assessment at day 1 each cycle (-2/+3 days thereafter)

The baseline assessments may be used at cycle 1, day 1 if within 7 days therapy of cycle 1 is started.

- Physical examination including height, weight, vital signs (blood pressure, heart rate, respiratory rate, body temperature), ECOG-performance status, assessment of toxicity, concomitant medication
- Laboratory tests
    - Hematology panel: hemoglobin, hematocrit, RBC count, platelet count, WBC count with differential count with neutrophils, lymphocytes, monocytes
    - Chemistry panel: sodium, potassium, calcium, magnesium, phosphorus, chloride, serum creatinine, alkaline phosphatase, AST, ALT, total bilirubin, GGT, uric acid, total urea, total protein, albumin, amylase, lipase, creatine kinase, CRP, Glucose, LDH
    - Coagulation panel: INR, aPTT
    - Free T3 and T4 and TSH

    - Serum or urin pregnancy test (beta-HCG) for women of childbearing potential (within 7 days prior to start of the treatment) and every month Q4W while on treatment with study drug.
    - Blood draw (20ml) for translational research (2 Streck® tubes) + 9 ml in 1 EDTA tube only cycle 3, day 1

Assessment at day 8 each cycle (-2/+3 days thereafter)

- Physical examination including height, weight, vital signs (blood pressure, heart rate, respiratory rate, body temperature), ECOG-performance status, assessment of toxicity, concomitant medication
- Laboratory tests
    - Hematology panel: hemoglobin, hematocrit, RBC count, platelet count, WBC count with differential count with neutrophils, lymphocytes, monocytes

Assessment at day 15 each cycle (-2/+3 days thereafter)

- Physical examination including height, weight, vital signs (blood pressure, heart rate, respiratory rate, body temperature), ECOG-performance status, assessment of toxicity, concomitant medication
- Laboratory tests
    - Hematology panel: hemoglobin, hematocrit, RBC count, platelet count, WBC count with differential count with neutrophils, lymphocytes, monocytes
    - Chemistry panel: sodium, potassium, calcium, magnesium, phosphorus, serum creatinine, alkaline phosphatase, AST, ALT, total bilirubin
        - Blood draw for translational research 20ml in 2 Streck® tubes (only cycle 1) + 9 ml in 1 EDTA tube only cycle 3, day 1

Tumor response assessment (every 8 weeks (±7 days))

Tumor response evaluation has to be conducted every 8 weeks and is independent of actual therapy. Thus, even if study therapy is interrupted or delayed, tumor response assessment has to be done every 8 weeks (±7 days).

- Laboratory tests
    - Serum tumor markers: CEA, CA 19-9, CA 72-4
- Disease assessment by radiological imaging with CT chest and abdomen+ pelvis with iv contrast or CT chest + MRI abdomen + pelvis with iv contrast

Assessment of tumor response continues until PD is identified by the local investigators and repeated once 8 weeks after identification of PD. (This is necessary to allow the independent radiology review to overrule the local PD assment.)
During treatment tumor response will be assessed by the investigator according to RECIST v1.1 (radiological imaging by CT chest, abdomen, pelvis with i.v. contrast or CT scan of the chest and MRI of the abdomen, pelvis). All treatment decisions are based on the assessment of the local radiologists and local investigators.
CT and/or MRI scans will be independently reviewed after end of treatment. Images must be pseudonymised and filed at a DVD. For independent central review please sent the collected images after the patient completed study to:

PD Dr. med. Peter Thuss-Patience
c/o Sekr. Prof Bullinger
Charité Universitätsmedizin Medizin
Campus Virchow Klinikum
Medizinische Klinik mit SP Hämatologie, Onkologie und Tumorimmunologie
Studienzentrale gastrointestinale Tumore
Augusnburger Platz 1
13353 Berlin

Final staging (end of treatment)

Regular staging every 8 weeks will be performed until disease progression and the final imaging is repeated 8 weeks after identification of PD. (This is necessary to allow the central reviewer to disagree with the date of first PD as assessed by local radiology.)

The following assessments will be made if patient discontinues treatment:

- Physical examination including height, weight, vital signs (blood pressure, heart rate, respiratory rate, body temperature), ECOG-performance status, assessment of toxicity, concomitant medication
- Laboratory tests
  - Hematology panel: hemoglobin, platelets, WBC with neutrophils, lymphocytes, monocytes
  - Chemistry panel: sodium, potassium, calcium, magnesium, phosphorus, chloride, serum creatinine, alkaline phosphatase, AST, ALT, total bilirubin, GGT, uric acid, total urea, total protein, albumin, amylase, lipase, creatine kinase, CRP, Glucose, LDH, cholesterol, triglyceride

    - Coagulation: INR, aPTT
    - Free T3 and free T4 and TSH
    - Serum tumor markers: CEA, CA 19-9, CA 72-4
    - Urine dipstick (if protein ++, 24-hour urine analysis)
    - Serum or urin pregnancy test (beta-HCG) for women of childbearing potential
    - Blood draw (20ml in 2 Streck® tubes) + 9 ml in 1 EDTA tube for translational research
- Disease assessment by radiological imaging with CT chest and abdomen+ pelvis with iv contrast or CT chest + MRI abdomen + pelvis with iv contrast – unless disease progression seen in regular radiological imaging is the reason for disruption of therapy. Final imaging is repeated 8 weeks after identification of PD. (This is necessary to allow the central reviewer to disagree with the date of first PD as assessed by local radiology.)

8.3.3 30 days safety follow-up (±7 days)

- Physical examination, vital signs, performance status (ECOG), assessment of toxicity, concomitant medication
- Laboratory tests (hematology and chemistry panel large see above), including free T3 and free T4 and TSH (if not done in the last 8 weeks)

8.3.4 Extended safety follow-up

Given the potential risk for delayed immune-related toxicities, safety follow-up must be performed every 30 days (±7 days) up to 90 days after the last dose of avelumab administration.

The first follow up 30 days after the last dose of avelumab is obligatory a site visit. The extended safety follow-up beyond 30 days after last study drug administration should also be performed via a site visit. Only if the patient is unable to come to the study center it can be done via telephone call.

8.3.5 Follow up

All subjects will be followed every 3 months ± 28 days for up to two years after start of treatment.

In case of progressive disease after study treatment only:

- survival, disease status, protracted toxicity, further treatment

In any other case additionally:

- disease assessment (by radiological imaging via CT/MRI scan as listed above), physical examination including weight, vital signs, ECOG-performance status

8.4 Post-study treatment

After termination of the study treatment due to whatever reason, treatment of the individual patient will be continued at the treating physician's discretion taking into account current available treatment guidelines or clinical recommendations.

8.5 Study Duration

Period of subjects signing ICF:	16 months
Period from first patient in to last patient visit:	40 months
Maximal treatment duration of individual subject:	1 year
Maximal treatment duration of individual subject:	1 year
Maximal follow up:	1 year after treatment discontinuation

8.6 Study Termination

8.6.1 Regular Data Analysis by the Independent Data Monitoring Committee

The independent data monitoring committee will be informed about all SAEs from the time of the first patient in at 3 months, at 6 months and every 6 months thereafter. This way a continuous safety monitoring is available.

In case of any unexpectedly high toxicity a meeting of the IDMC will be arranged.

Interim analysis

To allow the option of earlier stopping for futility in case of unfavorable results, a standard two-stage phase II design according to Simon (1989) is applied. In the first stage, n = 33 patients with the endpoint (OS at 6 months) available are analyzed, and the trial is stopped if the number of „successes" is only 16 or lower. Otherwise, the study is continued until a total of 53 patients evaluable for the primary endpoint have been recruited.
The IDMC will discuss the results with the sponsor and decide together with the sponsor about continuation of the trial.

8.6.2 Patient withdrawal

Patients will be withdrawn from therapy based on the following reasons:

- withdrawal of informed consent to the study
- withdrawal of consent for further treatment
- post-consent determination of ineligibility based on safety criteria
- pregnancy
- lack of therapeutic efficacy, as evidenced by progression
- treatment related toxicity according to dose modification criteria (see section 9 of the protocol)
- study drug (avelumab) delay for > 4 weeks due to toxicity. If the toxicity does not resolve within 4 weeks, avelumab will be discontinued unless it is determined by the treating investigator that the patient might benefit from continuation of avelumab
- if standard treatment (paclitaxel, ramucirumab) needs to be delayed or discontinued, avelumab can be continued and the patient is allowed to stay on the trial if the treating investigator determines that the patients might benefit from avelumab.
- physician's judgment following an adverse event, unacceptable toxicity
- termination by the sponsor, a regulatory authority or stopped by recommendation oft he IDMC
- any other reason for withdrawal that the study physician or patient indicates is in the overall best interest of the patient

- for selected protocol violation by decision of the coordinating investigator (including any use of any non-study systemic anti-cancer therapy)
- death
- lost to follow-up

Subjects who are permanently discontinued from receiving investigational product will be followed for safety unless consent is withdrawn or the subject is lost to follow-up. All subjects will be followed for survival. Subjects who decline to return to the site for evaluations will be offered follow-up by phone every 6-12 weeks as an alternative.

In this protocol, patients will be permitted to continue study treatment even if the criteria for progressive disease according to RECIST 1.1 are met for the first time if the treating physician decides that the patient clearly benefits from the treatment and the risk/benefit ratio is favorable. This case needs to be discussed with the coordinating investigator in Berlin. If CT confirms progressive disease at least 4 weeks later (confirmed PD) the treatment must be stopped.

Withdrawal of consent

If consent is withdrawn, the subject will not receive any further study treatment. If the patient does not also explicitly withdraw consent for further observation, further observation and further data documentation has to be continued. However, all patients are requested to perform an end-of-treatment visit. If a patient dies prior to the last scheduled study visit, the date and cause of death will be recorded.

8.6.3 Study completion

The study population will be analyzed for the primary endpoint (OS at 6 months) when the last patient included has passed the 6 months assessment. When the last patient has passed the 3 months safety assessment the final safety analyses will be conducted. Further follow up for survival will be performed. The completion of the overall survival follow up will be the end of the trial.

9 DOSE MODIFICATION

Toxicity will be graded according to NCI CTCAE, version 5.0. Treatment modifications described below are applied according to this severity grading. Toxicities of severity grade 1 only will not lead to any dose reduction or cycle delay. The same holds for adverse reactions without any potential of serious or life-threatening complications according to the judgment of the physician (e.g. alopecia).
Presumably, severe overlapping toxicity between chemotherapies will not occur. Thus, in case of toxicity requiring treatment modification, this alteration should reflect the causal relationship of the respective drug(s). For example, if the toxicity is unequivocally caused by only one drug, a dosage modification of the other drugs is not required.
If toxicity requires a dosing delay or interruption of any drug for more than 4 weeks, from the scheduled dosing day, that drug should be discontinued. The patient can remain on study with the remaining drugs and will continue to be evaluated according to study procedures.
If more than one different type of toxicity occurs concurrently, the most severe grade will determine the modification. In case of a necessary dose reduction the lower dose level will be applied throughout the rest of the therapy without re-escalation.
If toxicity requires a cycle delay of more than 4 weeks from the scheduled dosing day the patient is taken off protocol treatment.
In case of acute allergic reactions of grade 3 or 4, the respective agent should be discontinued permanently; in case of grade 1 or 2, it is up to the physician to continue treatment without dose modification, if this is in the best interest of the patient.
Each dose modification or treatment delay of more than 3 days has to be documented in the eCRF, including the respective reason.

9.1 Toxicity at start of the following cycle

Patients must meet the following criteria before each new cycle (d1):

- Absolute neutrophil count ≥ 1,5 x 10^9/l and platelet count ≥ 100 x 10^9/l
- Any bilirubin elevation has resolved to ≤ 1,5 ULN, AST/ALT ≤ 3 ULN (in case of liver metastasis ≤ 5 ULN)
- Recovery from any treatment-related grade 3/4 non-hematological toxicity (except alopecia) to baseline or ≤ grade 1.

Patients not meeting the above criteria on the date scheduled for the new cycle must suspend the whole treatment; upon fulfillment of the above criteria, treatment has to be resumed. Treatment must not be continued with one or two substances only.
The only exeption is, if one drug is discontinued permanently. In this case treatment can continue with the remaining drugs. In all other cases all three drugs shoul be given together.
Dose adjustments are at the investigator's discretion insofar as they must take account of the patient's clinical situation and the suspected causal relationship between the toxicities and administration of the anticancer drugs. Dose re-escalation is not permitted. If the above criteria necessitate postponement for more than 4 weeks, the patient should be withdrawn from the study.

9.2 Toxicity during the cycle

Patients must meet the following criteria before administering paclitaxel on day 8 and avelumab, ramucirumab and paclitaxel on day 15:

- Absolute neutrophil count ≥ 1 x 10^9/l and platelet count ≥ 75 x 10^9/l
- Any bilirubin elevation has resolved to ≤ 1,5 ULN, AST/ALT ≤ 3 ULN (in case of liver metastasis ≤ 5 ULN)
- Recovery from any treatment-related grade 3/4 non-hematological toxicity (except alopecia) to baseline or ≤ grade 1.

If patient does not meet criteria to continue paclitaxel on day 8 (+3/-2days), this administration must not be postponed, but has to be skipped completely.
Patients not meeting the above criteria on day 15 must suspend the whole treatment; upon fulfillment of the above criteria, treatment has to be resumed. Treatment must not be continued with one or two substances only.

9.3 Guidelines for dose modifications for avelumab and managment of Avelumab-specific Adverse Events or Adverse Drug Reactions

9.3.1 Infusion-related reactions

Symptoms

- Fever
- Chills
- Rigors
- Diaphoresis
- Dyspnoea, wheezing
- Headache

Management

NCI-CTCAE Grade	Treatment Modification for Avelumab
**Grade 1 – mild**<br>Mild transient reaction; infusion interruption not indicated; intervention not indicated.	Decrease the avelumab infusion rate by 50% and monitor closely for any worsening.
**Grade 2 – moderate**<br>Therapy or infusion interruption indicated but responds promptly to symptomatic treatment (for example, antihistamines, NSAIDs, narcotics, IV fluids); prophylactic medications indicated for ≤ 24 h.	Temporarily discontinue avelumab infusion.<br>Resume infusion at 50% of previous rate once infusion-related reaction has resolved or decreased to at least Grade 1 in severity, and monitor closely for any worsening.
**Grade 3 or Grade 4 – severe or life-threatening**<br>Grade 3: Prolonged (for example, not rapidly responsive to symptomatic medication and/or brief interruption of infusion); recurrence of symptoms following initial improvement; hospitalization indicated for clinical sequelae.<br>Grade 4: Life-threatening consequences; urgent intervention indicated.	Stop avelumab infusion immediately and disconnect infusion tubing from the subject.<br>Subjects have to be withdrawn immediately from study avelumab and must not receive any further avelumab treatment.
- If avelumab infusion rate has been decreased by 50% or interrupted due to an infusion reaction, it must remain decreased for the next scheduled infusion. If no infusion reaction is observed in the next scheduled infusion, the infusion rate may be returned to baseline at the subsequent infusions based on investigator's medical judgment.- If hypersensitivity reaction occurs, the subject must be treated according to the best available medical practice.	

IV = intravenous; NCI-CTCAE = National Cancer Institute-Common Terminology Criteria for Adverse Event; NSAIDs = nonsteroidal anti-inflammatory drugs.

Table 2 Treatment Modification for Symptoms of Infusion-Related Reactions

9.3.2 Severese hypersensitivity reaction and flu-like symptoms

If hypersensitivity reaction occurs, the subject must be treated according to the best available medical practice. Subjects should be instructed to report any delayed reactions to the Investigator immediately.
For prophylaxis of flu-like symptoms, 25 mg of indomethacin or comparable nonsteroidal anti-inflammatory drug (NSAID) dose (for example, ibuprofen 600 mg, naproxen sodium 500 mg) may be administered 2 hours before and 8 hours after the start of each dose of avelumab IV infusion. Alternative treatments for fever (for example, paracetamol) may be given to subjects at the discretion of the Investigator.

9.3.3 Tumor lysis syndrome

In addition, since avelumab can induce antibody-dependent cell-mediated cytotoxicity, there is a potential risk of tumor lysis syndrome. Should this occur, subjects should be treated per the local guidelines and the management algorithm below.

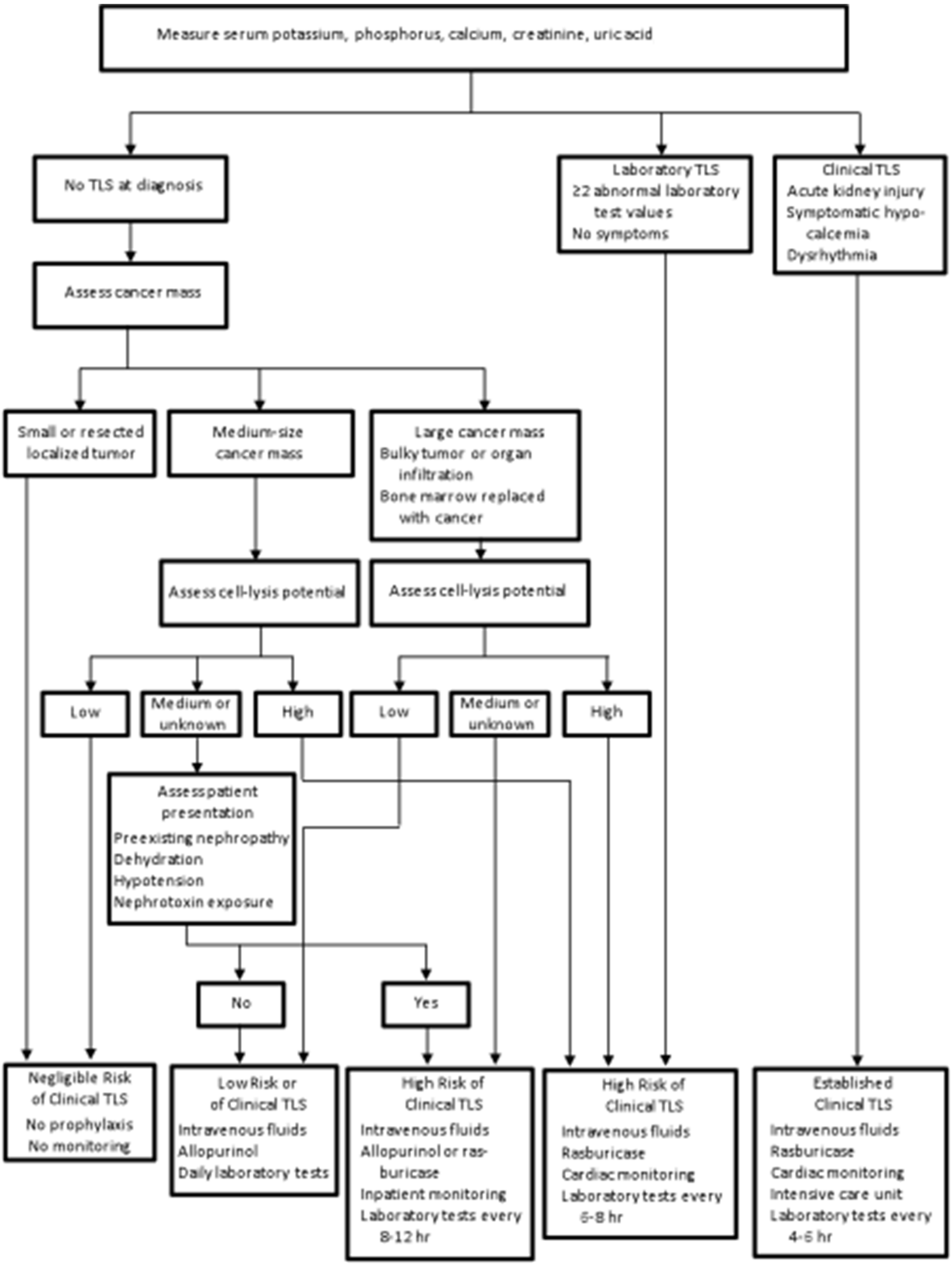

Figure 2 Management algorithm for tumor lysis syndrome

9.3.4 Immune-related Adverse Events

Gastrointestinal irAEs		
**Severity of Diarrhea/Colitis (NCI-CTCAE v4)**	**Initial Management**	**Follow-up Management**
Grade 1<br>Diarrhea: < 4 stools/day over Baseline<br>Colitis: asymptomatic	Continue avelumab therapy<br>Symptomatic treatment (e.g. loperamide)	Close monitoring for worsening symptoms<br>Educate subject to report worsening immediately<br>If worsens:<br>Treat as Grade 2, 3 or 4.
**Grade 2**<br>Diarrhea: 4 to 6 stools per day over Baseline; IV fluids indicated < 24 hours; not interfering with ADL<br>Colitis: abdominal pain; blood in stool	Withhold avelumab therapy<br>Symptomatic treatment	If improves to Grade ≤ 1:<br>Resume avelumab therapy<br><br>If persists > 5-7 days or recurs:<br>Treat as Grade 3 or 4.
**Grade 3 to 4**<br>Diarrhea (Grade 3): ≥ 7 stools per day over Baseline; incontinence; IV fluids ≥ 24 h; interfering with ADL<br>Colitis (Grade 3): severe abdominal pain, medical intervention indicated, peritoneal signs<br>Grade 4: life-threatening, perforation	Withhold avelumab for Grade 3.<br>Permanently discontinue avelumab for Grade 4 or recurrent Grade 3.<br><br>1.0 to 2.0 mg/kg/day prednisone IV or equivalent<br>Add prophylactic antibiotics for opportunistic infections<br>Consider lower endoscopy	If improves:<br>Continue steroids until Grade ≤ 1, then taper over at least 1 month; resume avelumab therapy following steroids taper (for initial Grade 3).<br><br>If worsens, persists > 3 to 5 days, or recurs after improvement:<br>Add infliximab 5mg/kg (if no contraindication). Note: infliximab should not be used in cases of perforation or sepsis.
**Dermatological irAEs**		
**Grade of Rash (NCI-CTCAE v4)**	**Initial Management**	**Follow-up Management**
**Grade 1 to 2**<br>Covering ≤ 30% body surface area	Continue avelumab therapy<br>Symptomatic therapy (for example, antihistamines, topical steroids)	If persists > 1 to 2 weeks or recurs:<br>Withhold avelumab therapy<br>Consider skin biopsy<br><br>Consider 0.5-1.0 mg/kg/day prednisone or equivalent. Once improving, taper steroids over at least 1 month, consider prophylactic antibiotics for opportunistic infections, and resume avelumab therapy following steroids taper.

		If worsens:<br>Treat as Grade 3 to 4.
**Grade 3 to 4**<br>**Grade 3:** Covering > 30% body surface area;<br>**Grade 4:** Life threatening consequences	Withhold avelumab for Grade 3.<br>Permanently discontinue for Grade 4 or recurrent Grade 3.<br>Consider skin biopsy<br>Dermatology consult<br>1.0 to 2.0 mg/kg/day prednisone or equivalent<br>Add prophylactic antibiotics for opportunistic infections	If improves to Grade ≤ 1:<br>Taper steroids over at least 1 month; resume avelumab therapy following steroids taper (for initial Grade 3).
**Pulmonary irAEs**		
**Grade of Pneumonitis (NCI-CTCAE v4)**	**Initial Management**	**Follow-up Management**
**Grade 1**<br>Radiographic changes only	Consider withholding avelumab therapy<br>Monitor for symptoms every 2 to 3 days<br>Consider Pulmonary and Infectious Disease consults	Re-assess at least every 3 weeks<br>If worsens:<br>Treat as Grade 2 or Grade 3 to 4.
**Grade 2**<br>Mild to moderate new symptoms	Withhold avelumab therapy<br>Pulmonary and Infectious Disease consults<br>Monitor symptoms daily; consider hospitalization<br>1.0 to 2.0 mg/kg/day prednisone or equivalent<br>Add prophylactic antibiotics for opportunistic infections<br>Consider bronchoscopy, lung biopsy	Re-assess every 1 to 3 days<br>If improves:<br>When symptoms return to Grade ≤ 1, taper steroids over at least 1 month, and then resume avelumab therapy following steroids taper<br>If not improving after 2 weeks or worsening:<br>Treat as Grade 3 to 4.
**Grade 3 to 4**<br>**Grade 3:** Severe new symptoms; New/worsening hypoxia;<br>**Grade 4:** Life-threatening	Permanently discontinue avelumab therapy.<br>Hospitalize.<br>Pulmonary and Infectious Disease consults.<br>1.0 to 2.0 mg/kg/day prednisone or equivalent<br>Add prophylactic antibiotics for opportunistic infections	If improves to Grade ≤ 1:<br>Taper steroids over at least 1 month<br>If not improving after 48 hours or worsening:<br>Add additional immunosuppression (for example, infliximab, cyclophosphamide, IV

	Consider bronchoscopy, lung biopsy	immunoglobulin, or mycophenolate mofetil)
**Hepatic irAEs**		
**Grade of Liver Test Elevation (NCI-CTCAE v4)**	**Initial Management**	**Follow-up Management**
**Grade 1**<br>**Grade 1** AST or ALT > ULN to 3.0 x ULN and/or Total bilirubin > ULN to 1.5 x ULN	Continue avelumab therapy	Continue liver function monitoring<br>If worsens:<br>Treat as Grade 2 or 3 to 4.
**Grade 2**<br>AST or ALT > 3.0 to ≤ 5 x ULN and/or total bilirubin > 1.5 to ≤ 3 x ULN	Withhold avelumab therapy<br>Increase frequency of monitoring to every 3 days.	If returns to Grade ≤ 1:<br>Resume routine monitoring; resume avelumab therapy.<br>If elevation persists > 5 to 7 days or worsens:<br>Treat as Grade 3 to 4.
**Grade 3 to 4**<br>AST or ALT > 5 x ULN and/or total bilirubin > 3 x ULN	Permanently discontinue avelumab therapy<br>Increase frequency of monitoring to every 1 to 2 days<br>1.0 to 2.0 mg/kg/day prednisone or equivalent<br>Add prophylactic antibiotics for opportunistic infections<br>Consult gastroenterologist/ hepatologist<br>Consider obtaining MRI/CT scan of liver and liver biopsy if clinically warranted	If returns to Grade ≤ 1:<br>Taper steroids over at least 1 month<br>If does not improve in > 3 to 5 days, worsens or rebounds:<br>Add mycophenolate mofetil 1 gram (g) twice daily<br>If no response within an additional 3 to 5 days, consider other immunosuppressants per local guidelines.
**Renal irAEs**		
**Grade of Creatinine Increased (NCI-CTCAE v4)**	**Initial Management**	**Follow-up Management**
**Grade 1**<br>Creatinine increased > ULN to 1.5 x ULN	Continue avelumab therapy	Continue renal function monitoring<br>If worsens:<br>Treat as Grade 2 to 3 or 4.
**Grade 2 to 3**<br>Creatinine increased > 1.5 and ≤ 6 x ULN	Withhold avelumab therapy<br>Increase frequency of monitoring to every 3 days<br>1.0 to 2.0 mg/kg/day prednisone or equivalent.<br>Add prophylactic antibiotics for opportunistic infections<br>Consider renal biopsy	If returns to Grade ≤1:<br>Taper steroids over at least 1 month, and resume avelumab therapy following steroids taper.<br>If worsens:<br>Treat as Grade 4.
**Grade 4**<br>Creatinine increased > 6 x ULN	Permanently discontinue avelumab therapy<br>Monitor creatinine daily	If returns to Grade ≤1:<br>Taper steroids over at least 1 month.

	1.0 to 2.0 mg/kg/day prednisone or equivalent.<br>Add prophylactic antibiotics for opportunistic infections<br>Consider renal biopsy<br>Nephrology consult	
**Cardiac irAEs**		
**Myocarditis**	**Initial Management**	**Follow-up Management**
New onset of cardiac signs or symptoms and / or new laboratory cardiac biomarker elevations (e.g. troponin, CK-MB, BNP) or cardiac imaging abnormalities suggestive of myocarditis.	Withhold avelumab therapy.<br>Hospitalize.<br>In the presence of life threatening cardiac decompensation, consider transfer to a facility experienced in advanced heart failure and arrhythmia management.<br>Cardiology consult to establish etiology and rule-out immune-mediated myocarditis.<br>Guideline based supportive treatment as per cardiology consult.*<br><br>Consider myocardial biopsy if recommended per cardiology consult.	If symptoms improve and immune-mediated etiology is ruled out, re-start avelumab therapy.<br><br>If symptoms do not improve/worsen, viral myocarditis is excluded, and immune-mediated etiology is suspected or confirmed following cardiology consult, manage as immune-mediated myocarditis.
Immune-mediated myocarditis	Permanently discontinue avelumab.<br>Guideline based supportive treatment as appropriate as per cardiology consult.*<br>1.0 to 2.0 mg/kg/day prednisone or equivalent<br>Add prophylactic antibiotics for opportunistic infections.	Once improving, taper steroids over at least 1 month.<br><br>If no improvement or worsening, consider additional immunosuppressants (e.g. azathioprine, cyclosporine A).
*Local guidelines, or eg. ESC or AHA guidelines<br>ESC guidelines website: https://www.escardio.org/Guidelines/Clinical-Practice-Guidelines<br>AHA guidelines website:<br>http://professional.heart.org/professional/GuidelinesStatements/searchresults.jsp?q=&y=&t=1001		
**Endocrine irAEs**		
**Endocrine Disorder**	**Initial Management**	**Follow-up Management**
**Grade 1 or Grade 2 endocrinopathies** (hypothyroidism, hyperthyroidism, adrenal insufficiency, type I diabetes mellitus)	Continue avelumab therapy<br>Endocrinology consult if needed<br><br>Start thyroid hormone replacement therapy (for hypothyroidism), anti-thyroid treatment (for hyperthyroidism), corticosteroids (for adrenal	Continue hormone replacement/suppression and monitoring of endocrine function as appropriate.

	insufficiency) or insulin (for Type I diabetes mellitus) as appropriate.<br><br>Rule-out secondary endocrinopathies (i.e. hypopituitarism / hypophysitis)	
**Grade 3 or Grade 4 endocrinopathies** (hypothyroidism, hyperthyroidism, adrenal insufficiency, type I diabetes mellitus)	Withhold avelumab therapy<br>Consider hospitalization<br>Endocrinology consult<br><br>Start thyroid hormone replacement therapy (for hypothyroidism), anti-thyroid treatment (for hyperthyroidism), corticosteroids (for adrenal insufficiency) or insulin (for type I diabetes mellitus) as appropriate.<br><br>Rule-out secondary endocrinopathies (i.e. hypopituitarism / hypophysitis)	Resume avelumab once symptoms and/or laboratory tests improve to Grade ≤ 1 (with or without hormone replacement/suppression).<br><br>Continue hormone replacement/suppression and monitoring of endocrine function as appropriate.
**Hypopituitarism/Hypophysitis** (secondary endocrinopathies)	If secondary thyroid and/or adrenal insufficiency is confirmed (i.e. subnormal serum FT4 with inappropriately low TSH and/or low serum cortisol with inappropriately low ACTH) :<br>Refer to endocrinologist for dynamic testing as indicated and measurement of other hormones (FSH, LH, GH/IGF-1, PRL, testosterone in men, estrogens in women)<br>Hormone replacement/suppressive therapy as appropriate<br>Perform pituitary MRI and visual field examination as indicated<br><br>If hypophysitis confirmed:<br>Continue avelumab if mild symptoms with normal MRI. Repeat the MRI in 1 month<br>Withhold avelumab if moderate, severe or life-threatening symptoms of hypophysitis and/or abnormal MRI. Consider hospitalization. Initiate corticosteroids (1 to 2 mg/kg/day prednisone or equivalent) followed by corticosteroids taper during at least 1 month.	Resume avelumab once symptoms and hormone tests improve to Grade ≤ 1 (with or without hormone replacement).<br><br>In addition, for hypophysitis with abnormal MRI, resume avelumab only once shrinkage of the pituitary gland on MRI/CT scan is documented.<br><br>Continue hormone replacement/suppression therapy as appropriate.

	Add prophylactic antibiotics for opportunistic infections.	
**Other irAEs (not described above)**		
**Grade of other irAEs (NCI-CTCAE v4)**	**Initial Management**	**Follow-up Management**
**Grade 2 or Grade 3 clinical signs or symptoms suggestive of a potential irAE**	Withhold avelumab therapy pending clinical investigation	If irAE is ruled out, manage as appropriate according to the diagnosis and consider re-starting avelumab therapy<br>If irAE is confirmed, treat as Grade 2 or 3 irAE.
**Grade 2 irAE or first occurrence of Grade 3 irAE**	Withhold avelumab therapy<br>1.0 to 2.0 mg/kg/day prednisone or equivalent<br>Add prophylactic antibiotics for opportunistic infections<br>Specialty consult as appropriate	If improves to Grade ≤ 1:<br>Taper steroids over at least 1 month and resume avelumab therapy following steroids taper.
**Recurrence of same Grade 3 irAEs**	Permanently discontinue avelumab therapy<br>to 2.0 mg/kg/day prednisone or equivalent<br>Add prophylactic antibiotics for opportunistic infections<br>Specialty consult as appropriate	If improves to Grade ≤ 1:<br>Taper steroids over at least 1 month.
**Grade 4**	Permanently discontinue avelumab therapy<br>to 2.0 mg/kg/day prednisone or equivalent and/or other immunosuppressant as needed<br>Add prophylactic antibiotics for opportunistic infections<br>Specialty consult.	If improves to Grade ≤ 1:<br>Taper steroids over at least 1 month
Requirement for 10 mg per day or greater prednisone or equivalent for more than 12 weeks for reasons other than hormonal replacement for adrenal insufficiency<br><br>Persistent Grade 2 or 3 irAE lasting 12 weeks or longer	Permanently discontinue avelumab therapy<br>Specialty consult	

Table 3 Management of Immune-mediated Adverse Reactions

Abbreviations: ACTH=adrenocorticotropic hormone; ADL=activities of daily living; ALT=alanine aminotransferase; AST=aspartate aminotransferase; BNP=B-type natriuretic peptide; CK-MB=creatine kinase MB; CT= computed tomography; FSH=follicle-stimulating hormone; GH=growth hormone; IGF-1=insulin-like growth factor 1; irAE=immune related adverse event; IV=intravenous; LH=luteinizing hormone; MRI=magnetic resonance imaging; NCI CTCAE=National Cancer Institute Common Terminology Criteria for Adverse Events; PRL=prolactin;T4=thyroxine; TSH=thyroid stimulating hormone; ULN=upper limit of normal.

9.4 Expected toxicities of paclitaxel and ramucirumab and guidelines for dose modifications for Ramucirumab

Because paclitaxel and ramucirumab are standard treatment and not the investigational medicinial product, dose modification can also be performed according to local guidelines at the discretion of the investigator.
For safety data regarding ramucirumab please refer to the SmPC.

In both studies, REGARD and RAINBOW, clinically meaningful efficacy outcomes were achieved with tolerable safety profiles for ramucirumab or ramucirumab and paclitaxel respectively. Overall, toxicities observed with ramucirumab were easily manageable with dose adjustments or supportive care. Table 4 depicts common toxicities observed with ramucirumab in combination with paclitaxel in the RAINBOW trial.

**event**	**ramucirumab + paclitaxel**		**ramucirumab + placebo**	
	CTC all grades (%)	CTC ≥3 (%)	CTC all grades (%)	CTC ≥3 (%)
**neutropenia**	54,4	40,7	31,0	18,8
**leukopenia**	33,9	17,4	21,0	6,7
**thrombocytopenia**	13,1	1,5	6,1	1,8
**hypoalbuminemia**	11,0	1,2	4,9	0,9
**hypertension**	25,1	14,7	5,8	2,7
**epistaxis**	30,6	0	7,0	0
**gastrointestinal bleeding**	10,1	3,7	6,1	1,5
**stomatitis**	19,6	0,6	7,3	0,6
**diarrhea**	32,4	3,7	23,1	1,5
**proteinuria**	16,8	1,2	6,1	0
**fatigue**	56,9	11,9	43,8	5,5
**edema**	25,1	1,5	13,7	0,6

Table 4 Toxicities of ramucirumab and paclitaxel observed in the RAINBOW trial

In line with other antiangiogenic treatments, ramucirumab was very well tolerated, and no unexpected toxicities were reported in the REGARD trial. The incidence of grade ≥ 3 adverse events (AEs) was similar between the two arms (57% with ramucirumab versus 58% with placebo). Hypertension was more common with ramucirumab (16% vs. 8%), although grade 3 hypertension was noted in only few patients (8% vs. 3%). Of note, grade ≥ 3 arterial thromboembolic events were slightly more common with ramucirumab (3% vs. 0%). On the other hand, ramucirumab was not associated with increased rates of proteinuria, bleeding, venous thrombosis, or gastrointestinal perforation. No increased rates of fatigue, decreased appetite, vomiting, anaemia and other notable toxic effect were

reported. In both study arms, 2% of the deaths were considered to be treatment related. Single agent ramucirumab is thus well tolerated with hypertension being the main issue. In the RAINBOW trial, overall, grade ≥ 3 AEs significantly increased with the addition of ramucirumab to paclitaxel (82% vs 63%). When combined with paclitaxel, the only other toxicity observed in addition to grade 3 hypertension (15% vs 3%) was a higher incidence of grade 3/4 neutropenia (41% vs. 19%) and leukopenia (17.4% vs. 6.7%), but not a higher rate of neutropenic fever (3.1% vs. 2.4%). AEs potentially associated with VEGF pathway inhibition were increased with ramucirumab, including proteinuria (17% vs. 6%) and bleeding (42% vs. 17%, mainly grade 1 and 2 epistaxis). In addition, fatigue (12% vs. 5%), diarrhea (4% vs. 1%), abdominal pain (6% vs. 3%) and peripheral neuropathy (8% cs. 5%) were more common in the ramucirumab plus paclitaxel group while neuropathy was mainly associated with a higher cumulative paclitaxel dose administered to patients in the combination arm. The increased frequency of AEs observed with ramucirumab, however, did not lead to a higher rate of treatment discontinuation. Accordingly, the incidence of treatment-related deaths was similar across treatment arms (4.0% vs 4.6%). A comparative analysis demonstrated that safety results achieved among patients from Western countries were consistent with those of the overall study population.

Dose adjustments for ramucirumab as per guidelines are below in Table 5 and should be followed. Further information (e.g. update of SmPC) must be considered. Additional guidelines for management of patients are also included below.

Event	Actions
**Reversible, non-life-threatening toxicity (for example, fatigue/anorexia/fever/laboratory abnormalities)**	First instance grade 3 or 4: 8 mg/kg (full dose) on recovery to Grade ≤1 within the same treatment cycle without interruption; Second instance grade 3 or 4: 6 mg/kg (first dose reduction) for next dose on recovery to Grade ≤1;Third instance grade 3 or 4: 5 mg/kg (second dose reduction) for next dose on recovery to Grade ≤1; Subsequent instance grade 3 or 4: discontinue (if a third dose reduction is required)
**Infusion-related reactions**	Reduce infusion rate by 50% for grade 1 or 2. Permanently discontinue for grade 3 or 4.
**Hypertension**	Interrupt ramucirumab for severe hypertension until controlled with medical management. Permanently discontinue if severe hypertension cannot be controlled with antihypertensive therapy.

**Proteinuria**	Interrupt ramucirumab for urine protein levels >2g/24h. Reinitiate treatment at a reduced dose of 6mg/kg every 2 weeks once the urine protein level return to <2g/24h. If the urine protein level >2g/24h reoccurs, interrupt ramucirumab and reduce the dose to 5mg/kg every 2 weeks once the urine protein level returns to <2g/24h Permanently discontinue if urine protein level 3g/24h, if there is a third occurance of 2g/24h, or if protein level does not return to <2g within 2 weeks or in the setting of nephrotic syndrome
**Wound healing complications**	Interrupt ramucirumab prior to scheduled surgery until the wound is fully healed
**Arterial thromboembolic events, or any PE/DVT occurring or worsening during anticoagulant therapy**	Permanently discontinue
**Gastrointestinal perforation**	Permanently discontinue
**Reversible Posterior Leukoencephalopathy (RPLS)**	Permanently discontinue
**Hepatic encephalopathy/serious liver impairments (e.g. hepatorenal syndrome)**	Permanently discontinue
**Grade 3 or 4 bleeding**	Permanently discontinue

Table 5 Dose modification guidelines of ramucirumab for non-hematologic toxicities

Dose modifications are not required for hematologic laboratory abnormalities unless associated with clinical symptoms. As an initial step, the dose of chemotherapy should be reduced first before any dose reductions for ramucirumab are considered.

9.5 Guidelines for dose modifications for paclitaxel

Because paclitaxel and ramucirumab are standard treatment and not the investigational medicinial product, dose modification can also be performed according to local guidelines at the discretion of the investigator.

For safety data regarding paclitaxel please refer to the SmPC.

Dose reduction of paclitaxel should be done according to toxicity messured by NCI CTCAE v5.0.

Following a hematological grade 4 toxicity or non-hematological grade 3 toxicity the paclitaxel dose should be reduced by $10mg/m^2$ for all futher cycles. A second reduction by $10mg/m^2$ is recommended if toxicity does not resolve or reappears. Please refer to the SmPC for further informations.

10 CRITERIA OF EVALUATION

10.1 Overall Survival Rate at 6 months

The Overall Survival Rate at 6 months (primary endpoint) will be determined by the proportion of ITT patients being alive 6 months after treatment start with first day first cycle divided by the total number of ITT patients, as defined in section 13.2.

10.2 Overall Survival

Overall survival is defined as time from first day first cycle to date of death from any cause (or censoring at the time point of last observation if the patient is still alive).

10.3 Overall Survival Rate at 12 months of treatment

The Overall Survival Rate at 12 months will be determined by the proportion of ITT patients being alive 12 months after treatment start with first day first cycle divided by the total number of ITT patients, as defined in section 13.2.

10.4 Progression free survival/Progression Free Survival Rate

Progression Free Survival (PFS) is defined as time from first day first cycle to date of first observed disease progression (according to RECIST v1.1) or death from any cause (or censoring at the time point of last date the patient was known to be without progression). A secondary analysis according to modified RECIST will be performed.
PFS and PFS rates at 6 and 12 months will be calculated according to Kaplan-Meier methods.

10.5 Response rate

The objective response rate is defined as the percentage of patients whose cancer shrinks (partial response, PR, based on RECIST criteria) or disappears (complete response, CR, based on RECIST criteria) during or after treatment. Objective response will be assessed according to RECIST v1.1. An exploratory analysis according to modified RECIST will

be performed. The confirmed objective response rate is based on remissions which are confirmed at least 4 weeks after the first date of response.
Objective response will be analysed according to RECIST 1.1 from the local investigator assessment and according to RECIST 1.1 and according to modified RECIST from the independent radiology assessment.

10.6 Duration of Response

Duration of response is the time from the day the criteria of response (CR or PR) were first met until the date of progression or the date the patient was last known to be without progression.

10.7 Safety endpoints

Safety assessments will include physical examinations including vital signs (blood pressure, heart rate), performance status (ECOG), clinical laboratory profile, concomitant medication and adverse events.
All observed toxicities and side effects will be graded according to NCI CTCAE v5.0 (NCI 2018) for all patients. The adverse events will also be analysed in accordance to their relation to the study treatment. Treatment related serious adverse events rate (SAE), defined as SAEs considered possibly, probably or definitely related to treatment, will be determined.

11 TRANSLATIONAL RESEARCH

11.1 Translational research projects

The planned translational research project is an integral part of this proof of concept trial and is aiming to investigate which patients achieve the maximal benefit. Therefore the purpuse of all translational investigations is to optimize the the experimental treatment of this trial (ramucirumab, paclitaxel, avelumab) for future studies.

The following translational research is currently planned, but may be adapted taking into account new research data

- Tumor-infiltrating lymphocytes (TiL) repertoire determination from tumor and liquid biopsy
- Liquid biopsy next-generation sequencing (NGS) immunoprofiling (TCRβ & IgH) before treatment initiation and before second avelumab dose to determine response predictive immune signature (diversification pattern as read-out for ongoing immune activation, TiL clone expansion in peripheral blood)
- Correlation of quantitative ctDNA and clonal dynamics with immune response signature to determine control of mutant subclones.
- FACS analysis of T-cell subpopulations
- In addition FFPE will be stained for PD-L1 (DAKO), analysed for microsatellite instability (MSI) and Epstein Barr Virus association (EBV) for subgroup analyses of all primary and secondary.

11.2 Sampling time points and materials

Tumor block:

The tumor block for TiL analysis, MSI, EBV and PD-L1 staining will be obtained at baseline.

We will obtain the most recent available **paraffin embedded tissue** from patients first diagnosis or metastases by the local pathologist (there is no planned new biopsie for this study). Preferrably we would like to get the tumor block or alternatively 12 unstained slides for further evaluation. The tissue will be immediately shipped using the prelabbeled envelopes to

PD Dr. med. Peter Thuss-Patience
c/o Sekr Prof Bullinger
Med. Klinik m.S. Hämatologie, Onkologie u. Tumorimmunologie
Charité Universitätsmedizin Berlin
Campus Virchow-Klinikum
Augustenburger Platz 1
13353 Berlin

Blood:

Blood samples for translational research will be taken at the following timepoints:

Time	Streck® Tube	EDTA tube
Prior to treatment	2 x 10ml (20ml) in Streck® tubes	1 x 9ml in EDTA tube
At day 15 cycle 1 (before second infusion of avelumab):	2 x 10ml in Streck® tube	1 x 9 ml in EDTA tube
at day 1 of cycle 3	2 x 10ml in Streck® tube	1 x 9 ml in EDTA tube
at progression or end of treatment	2 x 10ml in Streck® tube	1 x 9 ml in EDTA tube
	Streck® tubes:<br>Shippment **immediately** (!!) after each collection in prelabelled envelope to<br><br>**Universitätsklinikum Halle (Saale)**<br>**Klinik für Innere Medizin IV**<br>**AG Tumorimmunologie**<br>**Gebäude FG06 U01,**<br>**Büroräume 17.5, 17.6, 18,6**<br>**Ernst-Grube-Str. 40**<br>**06120 Halle (Saale)**	EDTA tube:<br>Shippment **immediately** (!!) after each collection in prelabelled envelope to<br><br>**PD Dr. med. Peter Thuss-Patience c/o Sekr Prof Bullinger**<br>**Med. Klinik m.S. Hämatologie, Onkologie u. Tumorimmunologie**<br>**Charité Universitätsmedizin Berlin**<br>**Campus Virchow-Klinikum**<br>**Augustenburger Platz 1**<br>**13353 Berlin**

Table 6: Translational research blood sampling

Lab kits, including the Streck® tubes, EDTA tubes, labels and working instructions will be provided.

Streck® tubes will be shipped to Halle,

Universitätsklinikum Halle (Saale)
Klinik für Innere Medizin IV
AG Tumorimmunologie
Gebäude FG06 U01, Büroräume 17.5, 17.6, 18,6
Ernst-Grube-Str. 40
06120 Halle (Saale)

EDTA tubes will be shipped to Berlin.

PD Dr. med. Peter Thuss-Patience
c/o Sekr Prof Bullinger
Med. Klinik m.S. Hämatologie, Onkologie u. Tumorimmunologie
Charité Universitätsmedizin Berlin
Campus Virchow-Klinikum
Augustenburger Platz 1
13353 Berlin

Each blood sample must be shipped as fast as possible directly after collection because analyses can only be done for a limited number of hours after collection.

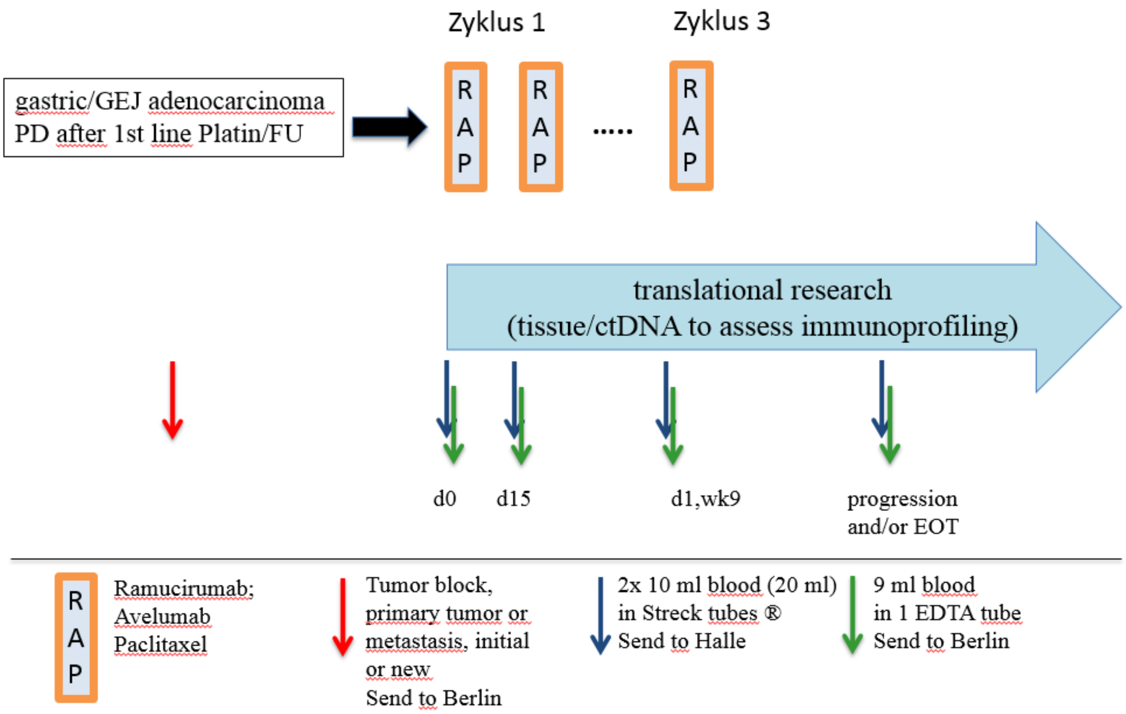

Figure 3 Overview of translational research

11.3 Storage and future use of biological samples

Upon receipt in the central laboratory the obtained blood will be separated into leucocytes and plasma and stored together. Leucocytes, plasma and the obtained tissue will be stored in the central laboratory until the preplanned analysis will be conducted when the clinical data from the trial are available. Further storage for up to 15 years from first patient in is intended to enable future evaluation of leucocyte, plasma or tissue based markers relevant for the further development of combination regimen with checkpoint inhibitors, chemotherapy and VEGF targeting drugs.

11.4 Usage of translational data

The translational data obtained will be analyzed and published in conjunction and under participation of the coordinating investigator and vize investigator and translational research coordinator.

12 ASSESSMENT OF ADVERSE EVENTS

It is the responsibility of the investigators to document all adverse events occurring during the study in the eCRF.

The following reportable events must be submitted to the sponsor within 24 hours (or immediately for death or life-threatening events) using the applicable safety report form provided. The sponsor will assume responsibility for submitting the reportable event(s) and ensure that any local reporting requirements are completed.

- Serious Adverse Events
- Exposure during Pregnancy or Breastfeeding (even if not associated with an adverse event)
- Occupational exposure (even if not associated with an adverse event)
- Potential drug-induced liver injury (Hy's Law cases; see https://www.fda.gov/downloads/Guidances/UCM174090.pdf): These events are considered important medical events and should be reported as SAEs.

Contact information for submission of reportable events to Sponsor:

Fax: [+49 30 450 553908]

OR

E-mail: [magenkarzinom@charite.de

Specifying:
PROTOCOL Number and/or Title
SUBJECT Number
SITE Number/PI Name
SAE/ONSET DATE

12.1 Independent Data Monitoring Committee

An independent data monitoring committee will follow the progress of the clinical trial, evaluate the safety parameters and will propose changes, ending or continuing of the trial to the sponsor.

12.2 Reference safety documents

The current edition of the SmPCs and the IB of Avelumab will be used as reference document and will be provided to the investigators in the Investigator's Site File.
With regards to the components Ramucirumab and Paclitaxel each of the drugs administered in the course of this study is market approved in Germany and all participating investigators will have broad experience with these therapies. Further Paclitaxel is available from several pharmaceutical companies (generic products) as well as in different formulations. Therefore, investigators will be provided with all relevant and up-to-date clinical and pre-clinical information (SmPC) on one example. It is the responsibility of the investigator to have at hand the most recent information on the specific medicinal product used during local routine at the respective study site.

12.3 Adverse event defintion

12.3.1 Adverse events

The International Conference on Harmonization (ICH) Guideline for Good Clinical Practice (GCP) E6(R1) defines an AE as:
Any untoward medical occurrence in a patient or clinical investigation subject administered a pharmaceutical product and which does not necessarily have a causal relationship with this treatment. An AE can therefore be any unfavorable and unintended sign (including an abnormal laboratory finding), symptom, or disease temporally associated with the use of a medicinal product, whether or not considered related to the medicinal product.
An AE includes but is not limited to any clinically significant worsening of a subject's pre-existing condition. An abnormal laboratory finding (including ECG finding) that requires an action or intervention by the investigator, or a finding judged by the investigator to represent a change beyond the range of normal physiologic fluctuation, should be reported as an AE.
Adverse events may be treatment emergent (i.e., occurring after initial receipt of investigational product) or non-treatment emergent. A non-treatment-emergent AE is any new sign or symptom, disease, or other untoward medical event that begins after written informed consent has been obtained but before the subject has received investigational product.

Elective treatment or surgery or preplanned treatment or surgery (that was scheduled prior to the subject being enrolled into the study) for a documented pre-existing condition, that did not worsen from baseline, is not considered an AE (serious or non-serious). An untoward medical event occurring during the prescheduled elective procedure or routinely scheduled treatment should be recorded as an AE or SAE.
The term AE is used to include both serious and non-serious AEs.

12.3.2 Serious adverse events

A serious adverse event is an AE occurring during any study phase (i.e., screening, run-in, treatment, wash-out, follow-up), at any dose of the study drugs that fulfils one or more of the following criteria:

- Results in death
- Is immediately life-threatening
- Requires in-patient hospitalization or prolongation of existing hospitalization
- Results in persistent or significant disability or incapacity
- Is a congenital abnormality or birth defect in offspring of the subject
- Is an important medical event that may jeopardize the patient or may require medical intervention to prevent one of the outcomes listed above
- Medical or scientific judgment should be exercised in deciding whether expedited reporting is appropriate in this situation. Examples of medically important events are intensive treatment in an emergency room or at home for allergic bronchospasm, blood dyscrasias, or convulsions that do not result in hospitalizations; or development of drug dependency or drug abuse.

A hospitalization meeting the regulatory definition for “serious” is any inpatient hospital admission that includes a minimum of an overnight stay in a health care facility.
Hospitalization for performing of protocol-required procedures or administration of study treatment or hospitalizations for procedures planned prior to study start and elective hospitalizations are not classified as an SAE.
Any clinically uncomplicated grade 3 or 4 leukopenia or neutropenia is not regarded as an SAE, though it must be documented as AE
Progression of the underlying malignant disease and symptoms caused by progression of the underlying tumor disease need not to be reported as SAE in this

protocol, unless progression or symptoms of progression are assessed as causally related to study medication or result in death.

SAEs must be submitted to the sponsor within 24 hours (or immediately for death or life-threatening events) using the applicable safety report form provided. SAEs are recorded continuously from time of signed informed consent until 30 days after last dose of combination. Immune related SAEs that are in a reasonable causality (possible, probable, definitive) to Avelumab must be reported up to 90 days after last treatment. Immune relates SAEs must be followed up until complete resolution.

12.3.3 Unexpected adverse events/SUSAR

An unexpected adverse event is any adverse drug event, the specificity or severity of which is not consistent with the current reference safety documents of the IMPs. Also, reports which add significant information on specificity or severity of a known, already documented adverse event constitute unexpected adverse events. An event more specific or more severe than described in the reference safety document would be considered "unexpected".

A suspected unexpected serious adverse reaction (SUSAR) is a serious adverse reaction, the nature, or severity of which is not consistent with the reference safety documents. All suspected adverse reactions related to the IMP which occur in this trial and are both unexpected and serious (SUSARs) are subject to expedited reporting.

SAEs which are "related" and "unexpected" meet the criteria for SUSAR.

12.3.4 Other reportable events

Pregnancy is a reportable event in this study.

12.4 Assessment of relationship – adverse drug reaction

The causal relationship to study drug is determined by a physician and should be used to assess all adverse events (AE). The causal relationship can be one of the following:

- Related: There is a reasonable causal relationship between study drug administration and the AE.

- Not related: There is not a reasonable causal relationship between study drug administration and the AE.

The term „reasonable causal relationship“ means there is evidence to suggest a causal relationship.

In case of a missing causality assessment in the eCRF or SAE reporting form, the event will be regarded as “probably related” unless further specified.

A serious ADR (SADR) is an adverse drug reaction that meets the definition of a serious event (provided below).

12.4.1 Practical guidance for causality assessments:

An adverse event will not be considered possibly related to study treatment if it:

- may be judged to be due to extraneous causes such as disease or environment or toxic factors.
- may be judged to be due to the subject’s clinical state or other therapy being administered.
- is not biologically plausible that the event is related to study medication.
- does not reappear or worsen when study treatment is re-administered.
- does not follow a temporal sequence from administration of study treatment.

An adverse event will be considered possibly related to study treatment if it:

- follows a temporal sequence from administration of study treatment.
- is a known response to the investigational product based on clinical or preclinical data.
- could not be explained by the known characteristics of the subject’s clinical state, environmental or toxic factors, or other therapy administered to the subject.
- disappears or decreases upon cessation or reduction of dose of study treatment.
- reappears or worsens when study treatment is re-administered.

12.5 Assessment of severity

Intensity of adverse events will be graded using the National Cancer Institute Common Terminology Criteria for Adverse Events (CTCAE), version 5.0.

If an adverse event occurs which is not contained in the CTCAE version 5.0, the five-point scale below will be used.

Grade 1:	Mild; asymptomatic or mild symptoms; clinical or diagnostic observations only; intervention not indicated
Grade 2:	Moderate: minimal, local or noninvasive intervention indicated; limiting age-appropriate instrumental ADL
Grade 3:	Severe or medically significant but not immediately life-threatening; hospitalization or prolongation of hospitalization indicated; disabling; limiting self-care ADL
Grade 4:	Life-threatening consequences; urgent intervention indicated
Grade 5:	Death related to AE

12.6 Safety recording and reporting requirements

12.6.1 Recording periods

Non-serious and serious adverse events are recorded continuously from time of signed informed consent until 30 days after last dose of combination treatment with IMPs. Immune related AEs and irSAEs that are in a reasonable causality (possible, probable, definitive) to Avelumab have to be reported up to 90 days after last treatment.
Immune relates AEs and irSAEs have to be followed up until complete resolution.
Pregnancies occurring in a study subject are recorded from time of signed informed consent until 6 months after last dose of IMPs. Pregnancies occurring in a partner of a study subject are recorded from time of signed informed consent until 6 months after last dose of IMPs.

12.6.2 Recording and reporting requirements

All adverse events:

The investigator is responsible for ensuring that all adverse events observed by the investigator or reported by patient are properly captured in the patients' medical records. Adverse events will be recorded in the AE page of the eCRF using a recognized medical term or diagnosis that accurately reflects the event. Adverse events will be assessed by the investigator for severity, relationship to the investigational product, possible etiologies, and whether the event meets criteria of an SAE and therefore requires expedited reporting.

The following variables will be collected for each AE:

- AE (verbatim)
- The date when the AE started and stopped
- Changes in NCI CTCAE grade and the maximum CTC grade attained
- Whether the AE is serious or not
- Investigator causality rating against avelumab (yes or no) or standard chemotherapies (ramucirumab and paclitaxel)
- Action taken with regard to IMPs:
    - none
    - study drug temporarily interrupted
    - study drug dose modifications
    - study drug permanently discontinued.
- Outcome:
    - recovered/resolved
    - recovered/resolved with sequelae
    - not recovered/not resolved
    - fatal
    - unknown (only applicable if patient is lost to follow-up);

In addition, the following variables will be collected for SAEs as applicable:

- Date AE met criteria for serious AE
- Date investigator became aware of serious AE
- Seriousness criterion date of hospitalization
- Date of discharge
- Probable cause of death
- Date of death
- Autopsy performed
- Description of AE
- Causality assessment in relation to other Study procedure(s)

Serious adverse events:

- For each subject any adverse event or abnormal laboratory test value that is serious whether or not considered causally related to the investigational products, or to the study procedure(s) occurring during the course of the study must be reported immediately (**within 24 hours**) after awareness to the sponsor via fax utilizing a

completed SAE Report Form. Missing information has to be sent as a follow-up report as soon as possible.

- Serious Adverse Events that are **unexpected** and **considered related** to IMPs and occur **after the completion of the trial** should be reported to the sponsor within one working day [ICH E2A III.E.3].
- If an investigator learns of any SAEs, including death, at any time **after the subject has been permanently withdrawn from study** (e.g. patient completed follow-up), and he/she considers there is a reasonable possibility that the event is related to study treatment, the investigator should notify the study sponsor.
- Potential drug-induced liver injury (Hy's Law cases): These events are considered important medical events and should be reported as SAEs

Pregnancies:

- Pregnancies occurring in a study subject or partner of a study subject are reported **within 24 hours** of knowledge of the event to the CRO using the Pregnancy Report Form.
- If an SAE is experienced in addition to or related to the pregnancy e.g. an induced or spontaneous abortion, also an SAE Report has to be sent to the CRO within 24 hours of first knowledge.

Abnormal laboratory results:

In general it is the investigator's responsibility to review all abnormal laboratory results and to determine if a given value represents a clinically significant change compared to previously obtained values and results in an Adverse Event or not.

- Abnormal laboratory test results will be recorded on the laboratory results pages of the eCRF. Laboratory-test-value abnormalities should additionally be considered an AE in case they are:
  - Accompanied by clinical symptoms
  - Leading to a change in study medication (e.g. dose modification, interruption or permanent discontinuation)
  - Requiring a change in concomitant therapy (e.g. addition of, interruption of, discontinuation of, or any other change in a concomitant medication, therapy or treatment)

- Any laboratory result abnormality fulfilling the criteria for a serious adverse event (SAE) should be reported as such, in addition to being recorded as an adverse event in the eCRF.

Occupational exposure (even if not associated with an adverse event)

This event has to be reported to the sponsor.

12.6.3 Sponsor obligations

The sponsor will ensure compliance with all regulatory reporting requirements including the notification of the appropriate Ethics Committees, Competent Authority and participating investigators of all serious adverse events occurring at the sites in accordance with national law, ICH Good Clinical Practice and European / EMA requirements.

- A sponsor representative will medically review all SAE reports and perform the expectedness assessment.
- A sponsor representative will forward SAE and pregnancy reports within one working day to the Coordinating Investigator (CI/LKP) and Merck.
- Every SAE, being assessed by either the investigator or the sponsor as suspected to be related to IMP und assessed as being either unexpected or unexpected with regard to outcome or severity of the event will be reported by the sponsor as SUSAR to the competent authority, responsible ethics committee and investigators of the trial in line with the national regulations in effect (German drug law [AMG] and GCP-V § 13).
    - Fatal or life-threatening SUSARs must be reported as soon as possible, but no later than 7 days; further important information to these cases may be reported as follow-up within additional 8 days. All others SUSARs have to be reported no later than 15 days.
    - Also all adverse events which can change the benefit-risk ratio of the study drugs or otherwise fulfil the criteria outlined in GCP-V §13 Abs.4 have to be handled/reported as SUSARs.

12.7 Handling of safety parameters

12.7.1 Adverse events

Adverse events can be spontaneously reported or elicited during open-ended questioning, examination, or evaluation of a subject. (In order to prevent reporting bias, subjects should not be questioned regarding the specific occurrence of one or more AEs.)
It will be left to the investigator's clinical judgment to determine whether an adverse event is related and of sufficient severity to require the subject's removal from treatment or from the study. A subject may also voluntarily withdraw from treatment due to what he or she perceives as an intolerable adverse event. If either of these situations arises, the subject should be strongly encouraged to undergo an end-of-treatment assessment and be under medical supervision until symptoms cease or the condition becomes stable.

12.7.2 Treatment and follow-up of adverse events

During the course of the study all AEs and SAEs should be proactively followed up for each subject. Every effort should be made to obtain a resolution for all events, even if the events continue after discontinuation/study completion. The investigator is responsible for following all SAEs until resolution, until the subject returns to baseline status, or until the condition has stabilized with the expectation that it will remain chronic, even if this extends beyond study participation and has to report it to the sponsor. At least 14 days after first report of the SAE a follow up report has to be sent to the sponsor.

12.7.3 Follow-up of abnormal laboratory test values

In the event of unexplained abnormal laboratory test values, the tests should be repeated immediately and followed up until they have returned to the normal range and/or an adequate explanation of the abnormality is found. If a clear explanation is established it should be recorded on the eCRF.

12.7.4 Overdose

There is currently no specific treatment for overdose with avelumab, paclitaxel or ramucirumab. The investigator will use clinical judgment to treat any overdose.

12.7.5 Pregnancy and contraception

Reproductive status

For this trial, male subjects will be considered to be of non-reproductive potential if they have azoospermia (whether due to having had a vasectomy or due to an underlying medical condition).

Female subjects will be considered of non-reproductive potential if they are either:

- postmenopausal (defined as at least 12 months with no menses without an alternative medical cause; in women < 45 years of age a high follicle stimulating hormone (FSH) level in the postmenopausal range may be used to confirm a post-menopausal state in women not using hormonal contraception or hormonal replacement therapy. In the absence of 12 months of amenorrhea, a single FSH measurement is insufficient.);

OR

- have had a hysterectomy and/or bilateral oophorectomy, bilateral salpingectomy or bilateral tubal ligation/occlusion, at least 6 weeks prior to screening;

OR

- has a congenital or acquired condition that prevents childbearing.

Counseling of study subjects and partners:

Subjects should be informed that taking the study medication may involve unknown risks to the fetus (unborn baby) if pregnancy were to occur during the study. In order to participate in the study subjects of childbearing potential must adhere to the contraception requirements. If there is any question that a subject of childbearing potential will not reliably comply with the requirements for contraception, that subject should not be entered into the study.

- Maternal exposure: Women of childbearing potential (WOCBP) must use appropriate method(s) of contraception. WOCBP should use an adequate method to avoid pregnancy for 30 days after the last dose of IMPs and 3 months after the

last dose of ramucirumab. A female patient must be instructed to immediately inform the investigator if she becomes pregnant during the study. Monitoring of the patient should continue until conclusion of the pregnancy.

- Paternal exposure: Men who are sexually active with WOCBP must use any contraceptive method with a failure rate of less than 1% per year. Men receiving IMPs and who are sexually active with WOCBP will be instructed to adhere to contraception for a period of 30 days after the last dose of investigational products. Male subjects must refrain from donating sperm during the study and for 30 days after the last dose of IMP and 3 months after the last dose of ramucirumab. A male study subject must be instructed to immediately inform the investigator if a pregnancy occurs in his partner during the study and up to 6 months after last dose of IMP.

At a minimum, subjects must agree to the use of two methods of contraception, with one method being highly effective and the other method being either highly effective or less effective as listed below.

In case hormonal contraception is used the second method of contraception must be a barrier method.

Highly effective methods of contraception

- Hormonal methods of contraception including oral contraceptive pills (combination of estrogen and progesterone), vaginal ring, injectables, implants and intrauterine devices (IUDs)
- Nonhormonal IUDs, such as ParaGard®
- Bilateral tubal ligation
- Vasectomy
- Complete Abstinence*

*Complete abstinence is defined as complete avoidance of heterosexual intercourse and is an acceptable form of contraception for all study drugs. Acceptable alternate methods of highly effective contraception must be discussed in the event that the subject chooses to forego complete abstinence.

Unacceptable methods of contraception

- Diaphragm with spermicide
- Cervical cap with spermicide

- Vaginal sponge
- Condom
- Withdrawal (coitus interruptus)
- Progestin only pills by WOCBP subject or male subject's WOCBP partner

Periodic abstinence (calendar, symptothermal, post-ovulation methods)

Pregnancies

Pregnancy itself, or pregnancy of a subject's partner, is not regarded as an adverse event unless there is a suspicion that the investigational product under study may have interfered with the effectiveness of a contraceptive medication. Congenital abnormalities/birth defects and spontaneous miscarriages should be reported and handled as SAEs. Elective abortions without complications should not be handled as AEs. The outcome of any conception occurring from the date of the first dose until 6 months (female subjects) or 6 months (partners of male subjects) after the last dose (spontaneous miscarriage, elective termination, ectopic pregnancy, normal birth or congenital abnormality) should be followed up and documented even if the subject was withdrawn from the study.

Subjects who become pregnant during the study period must not receive additional doses of investigational product but will not be withdrawn from the study until the necessary safety follow-up has been completed. The investigator should counsel the subject; discuss the risks of continuing the pregnancy, and possible effects on the fetus. The pregnancy will be followed for outcome of the mother and child (including any premature terminations) and should be reported to the sponsor.

Pregnancy of a subject's partner is not considered to be an AE. However, the outcome of all pregnancies (spontaneous miscarriage, elective termination, ectopic pregnancy, normal birth, or congenital abnormality) occurring from the date of the informed consent until 6 months after the last dose should, if possible, be followed up and documented. The investigator should counsel the subject's partner; discuss the risks of continuing the pregnancy, and possible effects on the fetus.

Where a report of pregnancy is received, prior to obtaining information about the pregnancy, the Investigator must obtain the consent of the subject's partner. Sponsor will provide a partner ICF in line with local procedures and submit it to the relevant Ethics Committees (ECs)/Institutional Review Boards (IRBs) prior to use.

12.8 Adverse drug reaction with concomitant medication

The investigators must be aware that for all concomitant medications the regulations of post marketing reporting for suspected adverse drug reactions apply, i.e. reporting to the marketing authorization holder or the local regulatory bodies.

13 DATA ANALYSIS AND STATISTICAL CONSIDERATIONS

13.1 General design and sample size estimation

The study is planned as single arm phase II study.

The primary endpoint of the study is the overall survival rate at 6 months based on the ITT population.

The experimental therapy would be considered to be a highly promising candidate for further development (e.g. in a phase III trial), if the true OS rate amounted to 65% or more.

Paclitaxel/ramucirumab could achieve an OS rate at 6 months of 65% in the Western population of the Rainbow trial, which set paclitaxel/ramucirumab as a standard treatment (Shitara et al. 2016). The Western patient population included in the Rainbow trial had better prognostic parameters than we expect for our study population (no pretreatment with taxanes, we expect 30% of patients being pretreated with a taxane either perioperatively of during 1st-line (Al-Batran et al. 2017). Only 30% of GE-junction tumors in the Rainbow population which have a slightly worse prognosis than tumor locations in the gastric body (Dikken et al. 2013), we expect 50% of GE-junction tumors in our study population). Although we expect worse prognostic parameters in our study population, we hope to achieve an OS rate of 65% at 6 months by combining paclitaxel / ramucirumab with avelumab.

On the other hand, the experimental therapy would be rated as insufficiently active, if the true OS rate is 50% or lower, as this suggests a distinct inferiority to paclitaxel / ramucirumab.

An overall survival rate of 45% at 6 months was detected in the metaanalysis pooling data from the three randomised trials comparing chemotherapy with best supportive care (Janowitz et al. 2016). In 410 pts chemotherapy significantly reduced the risk of death (hazard ratio (HR)=0.63, 95% confidence interval (CI)=0.51-0.77, P<0.0001) with a 6 months survival rate in the chemotherapy arm of 45%. Administering a triple combination of paclitaxel/ramucirumab + avelumab, we need to be clearly better than a monotherapy, therefore an OS rate at 6 months of less than 50% is considered as insufficient.

The following error levels are defined:

- Probability to accept the experimental therapy as promising (≥ 65% OS rate) with respect to efficacy, in spite of a true OS rate of ≤ 50%: 0.10 (type I error)
- Probability to reject the experimental therapy as not sufficiently efficient (≤ 50%), although the true OS rate is promising (≥ 65%): 0.2 (type II error, corresponding to a power of 80%).

According to these parameters, and allowing for the option of earlier stopping for futility in case of unfavorable results, a standard two-stage phase II design according to Simon (Simon 1989) is applied. In the first stage, n = 33 patients with the endpoint available are analyzed, and the trial is stopped if the number of "successes" is only 16 or lower. Otherwise, the study is continued until a total of 53 patients evaluable for efficacy as defined in section 13.2 have been recruited. The final conclusion of the phase II trial will depend on the definite OS rate (and its confidence interval), as well as the information on type, frequency and severity of toxicities. Formally, more than 31 patients alive at 6 months are required to reject the null hypothesis defined above. Assuming a 10% drop out rate, and in order to achieve adequate power in the per-protocol analysis (see below), we are planning to include 59 pts.

13.2 Analysis populations

The intention-to-treat population is defined as patients who received at least one dose of all three components of treatment (avelumab, ramucirumab and paclitaxel). The IIT population is evaluable for toxicity and as well for efficacy (intention-to-treat, ITT). The safety population consists of all patients who received at least 1 dose of any treatment. In addition, a per-protocol population is defined, consisting of all patients having received at least two full treatment cycles according to the protocol and with available survival status at 6 months after first dose of treatment.

13.3 Statistical methods

The primary endpoint is calculated by deviding the number of patients alive a 6 months by the total number of patients in the ITT population (ITT as defined in section 13.2). Exact 80%, 90% and 95% confidence intervals (two-sided) will be provided for this proportion. Formally, the lower boundary of the 80% confidence interval corresponds to the sample size estimation approach described above.

All other efficacy and toxicity parameters will be evaluated in an explorative or descriptive manner, providing proportions, means, medians, ranges, standard deviations and/or confidence intervals, or Kaplan-Meier estimates, as appropriate.

If p values are calculated (e.g. for comparison of subgroups), they will be presented explicitly without referring to hypotheses or a significance level. Usually, no error adjustment for multiple testing will be performed. Thus the p values will reflect the comparison-wise error and not the experiment-wise error. All p values will be two-sided if not stated otherwise.

Comparisons of the categorical data, e.g. response rates, will be performed using chi^2 test, Fisher's exact test or a trend test according to Cochran/Armitage, as appropriate. Secondary endpoints of time-to-event type, i.e. progression-free survival and overall survival, will be estimated according to Kaplan-Meier, and exploratively compared (between subgroups, or to historical data) using the logrank test. Hazard ratios (with confidence intervals) may be derived from corresponding Cox proportional hazard models.

Further details will be prospectively defined in a statistical analysis plan to be written before any efficacy data are analyzed.

14 DATA MANAGEMENT

14.1 Patient identification list

All included patients have to be documented in a confidential patient identification list. This list contains the patient specific numbers (patient-number) together with date of birth and the full name of the patient. Patient related data will be just transmitted in pseudonymized form. The identification list will stay at each center.

14.2 Data capture

All data will be entered directly at the center by the site staff using an electronic data capture system (EDC system, eCRF) which is also utilized for query management. Automatic edit checks will validate data directly during entry into the study database. Data will further be evaluated manually for consistency, accuracy and completeness regularly. After completion of data capture and subsequent data cleaning, the study database will be closed and the data will be transferred into the statistic software for analysis.

15 QUALITY ASSURANCE

15.1 Standardization

Criteria for assessing efficacy and safety endpoints will be standardized by using NCI-CTCAE Version 5.0 for safety issues, RECIST Version 1.1 and irRECIST for efficacy parameters. Every center has to reveal their laboratory norm values and their validation through certification.

15.2 Data Access

All source data have to be in the patients file under the responsibility of the investigator. Documentation in the eCRF must correspond to source data in the patient file. For this trial source data are defined as:

- medical and demographical data
- results of laboratory and imaging data
- selection criteria
- signed informed consent form (original)

15.3 Monitoring/Source Data Verification (SDV)

The monitoring will be conducted according to local requirements.
Monitoring will be performed by an external monitor. The study monitor will review the eCRF data for completeness and accuracy during the monitoring visits (source data verification / SDV). The study monitor will point out any discrepancies between source data and the data captured in the eCRF. The monitor will issue electronic queries to site staff to initiate discrepancy resolution. Discrepancies which require eCRF data corrections have to be re-solved by authorized site personnel by answering these monitoring queries.
The frequency of on-site visits will depend on the number of recruited patients. The monitor must be given access to subject medical records and other study-related records needed to verify the entries on the eCRF. The investigator agrees to cooperate with the monitor to ensure that any problems detected in the course of these monitoring visits, including delays in completing case report forms, are resolved. The investigator has to

ensure that all data required according to this protocol will be entered promptly in the eCRF.
Quality control of data will be done by reviewing the data entered into the trial software for consistency, accuracy and completeness. During on-site visits the correct transmission of data into the eCRF (source data verification) as well as informed consent forms, selection criteria, efficacy and safety parameters will be reviewed. The complete scale of the monitoring will be defined by the trial specific monitoring plan.

15.4 Audits and inspections

To ensure quality of data, study integrity, and compliance with the protocol and the various applicable regulations and guidelines, the sponsor may conduct site visits to institutions participating to protocols.
The investigator, by accepting to participate to this protocol, agrees to co-operate fully with any quality assurance visit undertaken by third parties, including representatives from the sponsor, national and/or foreign regulatory authorities or company supplying the product under investigation, as well as to allow direct access to documentation pertaining to the clinical trial (including eCRFs, source documents, hospital subject charts and other study files) to these authorized individuals.
The investigator must inform the sponsor immediately in case a regulatory authority inspection will be scheduled.

16 REGULATORY AND LEGAL OBLIGATIONS

16.1 General provisions/Declaration of Helsinki

This study is conducted in agreement with the ICH Harmonized Tripartite Guideline on Good Clinical Practice, valid since 17.01.1997, the Declaration of Helsinki (in its current version)) and the respective national laws in its current version). The Principle Investigator has more than two years of experience in the conduction of clinical drug trials.

16.2 Patient protection

The responsible investigator will ensure that this study is conducted in agreement with either the Declaration of Helsinki (in its current version) or the laws and regulations in its current version.

The protocol has been written, and the study will be conducted according to the ICH Harmonized Tripartite Guideline for Good Clinical Practice (reference: http://www.ich.org/fileadmin/Public_Web_Site/ICH_Products/Guidelines/Efficacy/E6/E6_R2__Step_4_2016_1109.pdf). The protocol will be approved by Independent Ethics Committees.

16.3 Competent authority

Prior to the start of the trial an application for authorization by the competent Higher Federal Authority is submitted by the sponsor including a copy of the protocol and other information and documents required by the competent national Higher Federal Authority. A copy of the written approval must be available before the start of recruitment of subjects into the study. All changes of the study protocol or other study document classified „substantial“ as well as adverse events will be announced to the CA (according to the appropriate Directives and national legal requirements). Once a year or whenever it is questioned the CA will get information about all SAR and about the security of the affected subjects, according to the appropriate Directives and national legal requirements. Recommendations and tips of the CA will be taken up into the study protocol. The sponsor will inform the CA about the course of the investigation in security aspects

according to the appropriate Directives and national legal requirements and also about the end and the results of the investigation.

16.4 Independent Ethics Committee

Prior to the start of the trial an application for the favorable opinion for Germany is submitted on behalf of the sponsor to the central independent, interdisciplinary ethics committee responsible under federal law for the principle investigator and to the local ethics committees responsible for the other participating institutions including a copy of the protocol, proposed informed consent form and other information and documents required by the ethics committees for their opinion. A copy of the written favorable opinion of the protocol and informed consent form must be available before the start of recruitment of subjects into the study. All changes of the study protocol or other study document classified “substantial” as well as adverse events, will be announced to the Independent Ethics Committee (IEC), according to the local requirements, e.g. for Germany §13, (2) und (3) GCP-V. Once a year or whenever it is questioned the IEC will get information about all SAR and about the security of the affected subjects, (e.g. according to §13. (6) GCP-V). Recommendations and tips of the IEC will be taken up into the study protocol. The sponsor will inform the IEC about the course of the Investigation in security aspects (e.g. according §13 GCP-V, (1) till (6)) and also about the end and the results of the investigation (e.g. according to §13 GCP-V, (8) and (9)).
The investigator cannot influence the decisions of the IEC. A list of the IEC members will be ordered.

16.5 Amendments

The appendices, attached to this protocol and referred to in the protocol, form an integral part of the protocol. No changes or amendments to this protocol may be made by the Investigator. The sponsor must submit and obtain favorable opinion/approval from the IEC and competent Higher Federal Authority for all subsequent protocol amendments. For changes to the informed consent form favorable opinion from the IEC might be necessary.

16.6 Study reports

After the end of the trial a clinical trial report will be written and provided to the IEC and competent Higher Federal Authority independent of the completion or a premature closure of the trial.

16.7 Informed consent

The informed consent form will be submitted together with the study protocol to the independent ethics committees (IEC) for review and approval. If requested, modifications must be incorporated. A copy of the written approval of the IEC must be available before starting the trial and dispensing any trial medication to trial subjects. The informed consent form must not be altered by the investigator except for contact data of the investigators. Changes to the informed consent form also have to be approved by the IEC. The revised form will be sent to all sites to replace the preceding version.
Before a subject's participation in the clinical study, the investigator must obtain written informed consent from the subject. All subjects will be informed of the aims of the study, the possible adverse events, the anticipated benefits, the procedures and possible hazards to which he/she will be exposed, and the mechanism of treatment allocation the subjects also will be informed about alternative treatments. Subjects will be informed of their insurance protection and the obligations which are linked to insurance. They will be informed as to the strict confidentiality of their subject data, but that their medical records may be reviewed for trial purposes by authorized individuals other than their treating physician. It will be emphasized that the participation is voluntary and that the subject is allowed to refuse further participation in the protocol whenever he/she wants. This will not prejudice the subject's subsequent care. The informed consent procedure must conform to the ICH guidelines on Good Clinical Practice.
The informed consent consists of three parts: consent to the diagnostic and therapeutic procedures of the trial, consent to the collection and storage of biological material, and consent to the processing and storage of data. The latter one includes consent to inspections where records may be reviewed by authorized individuals (other than their treating physician) of the sponsor or surveillance authorities / ethics committees. If the subject does not consent to the collection, processing and storage of his data, inclusion in the study is not possible and the subject's refusal should be documented in the medical notes. The subject must be informed about the aims, methods, anticipated benefits, and

potential hazards of the study and before any protocol-specific screening procedures or any study treatment are administered. The collection and storage of biological material in this clinical trial is optional; consent to this part of the trial is not necessary for the participation in this clinical trial.
The investigator is also responsible for asking the subject if the subject agrees to have his/her primary care physician informed of the subject's participation in the clinical study. If the subject agrees to such notification, the investigator shall inform the subject's primary care physician of the subject's participation in the clinical study.
If a potential subject is illiterate or visually impaired, the investigator must provide an impartial witness to read the informed consent form to the subject and must allow for questions. Thereafter, both the subject and the witness must sign the informed consent form to attest that informed consent was freely given and understood.
Adequate explanations of the aims, methods, anticipated benefits, and potential hazards of the study, the mechanism of treatment allocation must be given. The subject will have enough time to decide to participate in the study or not.
The acquisition of informed consent and the subject's agreement or refusal of his/her notification of the primary care physician must be documented in the subject's medical records, and the informed consent form must be signed and personally dated by the subject and by the investigator. One signed original of the informed consent form must be retained in accordance with institutional policy and another original must be provided to the subject. Treatment cannot start before the subject has signed the informed consent, meets all inclusion and no exclusion criteria and is registered.
With signing the informed consent form the investigator confirms that an individual clarification conversation has taken place and that the subject has signed the informed consent form.

16.8 Subject confidentiality

The investigator must ensure that the subject's confidentiality is maintained. On the case report forms, subjects should be identified by their subject study number and only on the SAE report form additionally the age.
In compliance with ICH-GCP Guidelines, it is required that the investigator and institution permit authorized representatives of the sponsor, and of regulatory agencies direct access to review the subject's original medical records for verification of study-related procedures and data. Direct access includes examining, analyzing, verifying, and

reproducing any records and reports that are important to the evaluation of the study. The investigator is obligated to inform and obtain the consent of the subject to permit named representatives to have access to his/her study-related records without violating the confidentiality of the subject. The investigator must keep a list for the identification of the subjects (including name, birthday, gender, date of informed consent, date of registration).

16.9 Study documentation and archive

The investigator must maintain a list of appropriately qualified persons to whom he/she has delegated study duties, including all those authorized to make entries and/or corrections on case report forms.

Source documents are original documents, data, and records from which the subject's case report form data are obtained. These include but are not limited to hospital records, clinical and office charts, laboratory and pharmacy records, diaries, microfiches, radiographs, and correspondence.

The investigator and study staff are responsible for maintaining a comprehensive and centralized filing system of all study-related (essential) documentation, suitable for inspection at any time by representatives from the study sponsor and/or applicable regulatory authorities. Elements include:

- Subject files containing completed case report forms, informed consent forms, and subject identification list.
- Study files containing the protocol with all amendments, the summary of product characteristics, copies of pre-study documentation, and all correspondence to and from the IEC.
- If kept, proof of receipt, Investigational Product Accountability Record, Return of Investigational Product for Destruction, Final Investigational Product Reconciliation Statement, and all drug-related correspondence.

In addition, all original source documents supporting entries in the case report forms must be maintained and be readily available.

All study documents and source documents must be kept for at least 10 years from submission of the final study report. Should the investigator wish to assign the study records to another party or move them to another location, he/she must notify the sponsor in writing of the new responsible person and/or the new location.

16.10 Compensation

Subjects will not be paid for participating in this clinical trial.

17 TRIAL SPONSORSHIP AND FINANCING

The Charité will be the legal sponsor according to German law an finances the trial. The study will be financially supported by an independent research grant provided by Merck upon a proposal of the principal investigator PD Dr. Peter Thuss-Patience. Merck will provide Avelumab for all patients.

18 TRIAL INSURANCE

According to the respective national law (e.g. § 40 (1) Nr. 8 und (3) German drug law (AMG)) the sponsor has taken out insurance policy for all subjects participating in the trial at HDI Global SE with the contract no 5701032603017. This insurance covers the sponsor, the investigator and his co-workers against liability in the event that a subject's health is injured during the course of the clinical trial.
The insurance policy provides benefits, even when no one else is liable for the damage death of or injury to any subject during the trial.

A certificate of insurance and conditions will be provided to the investigators and to every study participant with the informed consent form.

19 TRIAL REGISTRATION

Before start of the study the trial will be registered at the public registry ClinicalTrials.Gov.

20 PUBLICATION POLICY

After completing of all analyses, the statistical analysis and the conclusion will be published by the coordinating principal investigators (PD Dr. Thuss-Patience and PD Dr. Stein). Publication will be independent of the results, whether they were positive or negative. The manuscript completed for publication is accepted as final report.

All participating sites recruiting at least 10% of the patients will become a co-authorship if possible according to the publication policy of the journal. Persons involved in planning, conducting and evaluating the trial will be offered co-authorships. All co-authors will get the option to comment on the manuscript before publication.

21 APPENDIX

21.1 Bibliography

Al-Batran, Salah-Eddin, Nils Homann, Harald Schmalenberg, Hans-Georg Kopp, Georg Martin Haag, Kim Barbara Luley, Wolff H. Schmiegel, et al. 2017. "Perioperative Chemotherapy with Docetaxel, Oxaliplatin, and Fluorouracil/Leucovorin (FLOT) versus Epirubicin, Cisplatin, and Fluorouracil or Capecitabine (ECF/ECX) for Resectable Gastric or Gastroesophageal Junction (GEJ) Adenocarcinoma (FLOT4-AIO): A Multicenter, Randomized Phase 3 Trial." Journal of Clinical Oncology 35 (15_suppl): 4004–4004. https://doi.org/10.1200/JCO.2017.35.15_suppl.4004.

Bang, Yung-Jue, Kei Muro, Charles S. Fuchs, Talia Golan, Ravit Geva, Hiroki Hara, Shadia Ibrahim Jalal, et al. 2017. "KEYNOTE-059 Cohort 2: Safety and Efficacy of Pembrolizumab (Pembro) plus 5-Fluorouracil (5-FU) and Cisplatin for First-Line (1L) Treatment of Advanced Gastric Cancer." Journal of Clinical Oncology 35 (15_suppl): 4012–4012. https://doi.org/10.1200/JCO.2017.35.15_suppl.4012.

Bendell, Johanna C., John D. Powderly, Christopher Hanyoung Lieu, S. Gail Eckhardt, Herbert Hurwitz, Howard S. Hochster, Janet E. Murphy, et al. 2015. "Safety and Efficacy of MPDL3280A (Anti-PDL1) in Combination with Bevacizumab (Bev) and/or FOLFOX in Patients (Pts) with Metastatic Colorectal Cancer (MCRC)." Journal of Clinical Oncology 33 (3_suppl): 704–704. https://doi.org/10.1200/jco.2015.33.3_suppl.704.

Chau, Ian, Johanna C. Bendell, Emiliano Calvo, Rafael Santana-Davila, Hendrik-Tobias Arkenau, Gu Mi, Jin Jin, et al. 2017. "Ramucirumab (R) plus Pembrolizumab (P) in Treatment Naive and Previously Treated Advanced Gastric or Gastroesophageal Junction (G/GEJ) Adenocarcinoma: A Multi-Disease Phase I Study." Journal of Clinical Oncology 35 (15_suppl): 4046–4046. https://doi.org/10.1200/JCO.2017.35.15_suppl.4046.

Chau, Ian, Johanna C. Bendell, Emiliano Calvo, Rafael Santana-Davila, Jordi Rodon Ahnert, Nicolas Penel, Hendrik-Tobias Arkenau, et al. 2017. "Interim Safety and Clinical Activity in Patients (Pts) with Advanced Gastric or Gastroesophageal Junction (G/GEJ) Adenocarcinoma from a Multicohort Phase 1 Study of Ramucirumab (R) plus Pembrolizumab (P)." Journal of Clinical Oncology 35 (4_suppl): 102–102. https://doi.org/10.1200/JCO.2017.35.4_suppl.102.

Chung, Hyun Cheol, Hendrik-Tobias Arkenau, Lucjan Wyrwicz, Do-Youn Oh, Keun-Wook Lee, Jeffrey R. Infante, Sung Sook Lee, et al. 2016. "Avelumab (MSB0010718C; Anti-PD-L1) in Patients with Advanced Gastric or Gastroesophageal Junction Cancer from JAVELIN Solid Tumor Phase Ib Trial: Analysis of Safety and Clinical Activity." Journal of Clinical Oncology 34 (15_suppl): 4009–4009. https://doi.org/10.1200/JCO.2016.34.15_suppl.4009.

Dikken, Johan L., Raymond E. Baser, Mithat Gonen, Michael W. Kattan, Manish A. Shah, Marcel Verheij, Cornelis J. H. van de Velde, Murray F. Brennan, and Daniel G. Coit. 2013. "Conditional Probability of Survival Nomogram for 1-, 2-, and 3-Year

Survivors after an R0 Resection for Gastric Cancer." Annals of Surgical Oncology 20 (5): 1623–30. https://doi.org/10.1245/s10434-012-2723-6.

Ford, Hugo E. R., Andrea Marshall, John A. Bridgewater, Tobias Janowitz, Fareeda Y. Coxon, Jonathan Wadsley, Wasat Mansoor, et al. 2014. "Docetaxel versus Active Symptom Control for Refractory Oesophagogastric Adenocarcinoma (COUGAR-02): An Open-Label, Phase 3 Randomised Controlled Trial." The Lancet. Oncology 15 (1): 78–86. https://doi.org/10.1016/S1470-2045(13)70549-7.

Fuchs, Charles S., Toshihiko Doi, Raymond Woo-Jun Jang, Kei Muro, Taroh Satoh, Manuela Machado, Weijing Sun, et al. 2017. "KEYNOTE-059 Cohort 1: Efficacy and Safety of Pembrolizumab (Pembro) Monotherapy in Patients with Previously Treated Advanced Gastric Cancer." Journal of Clinical Oncology 35 (15_suppl): 4003–4003. https://doi.org/10.1200/JCO.2017.35.15_suppl.4003.

Fuchs, Charles S., Jiri Tomasek, Cho Jae Yong, Filip Dumitru, Rodolfo Passalacqua, Chanchal Goswami, Howard Safran, et al. 2014. "Ramucirumab Monotherapy for Previously Treated Advanced Gastric or Gastro-Oesophageal Junction Adenocarcinoma (REGARD): An International, Randomised, Multicentre, Placebo-Controlled, Phase 3 Trial." Lancet (London, England) 383 (9911): 31–39. https://doi.org/10.1016/S0140-6736(13)61719-5.

Hironaka, Shuichi, Shinya Ueda, Hirofumi Yasui, Tomohiro Nishina, Masahiro Tsuda, Takehiko Tsumura, Naotoshi Sugimoto, et al. 2013. "Randomized, Open-Label, Phase III Study Comparing Irinotecan with Paclitaxel in Patients with Advanced Gastric Cancer without Severe Peritoneal Metastasis after Failure of Prior Combination Chemotherapy Using Fluoropyrimidine plus Platinum: WJOG 4007 Trial." Journal of Clinical Oncology: Official Journal of the American Society of Clinical Oncology 31 (35): 4438–44. https://doi.org/10.1200/JCO.2012.48.5805.

Hodi, F. Stephen, Donald Lawrence, Cecilia Lezcano, Xinqi Wu, Jun Zhou, Tetsuro Sasada, Wanyong Zeng, et al. 2014. "Bevacizumab plus Ipilimumab in Patients with Metastatic Melanoma." Cancer Immunology Research 2 (7): 632–42. https://doi.org/10.1158/2326-6066.CIR-14-0053.

Janjigian, Yelena Yuriy, Johanna C. Bendell, Emiliano Calvo, Joseph W. Kim, Paolo Antonio Ascierto, Padmanee Sharma, Patrick Alexander Ott, et al. 2016. "CheckMate-032: Phase I/II, Open-Label Study of Safety and Activity of Nivolumab (Nivo) Alone or with Ipilimumab (Ipi) in Advanced and Metastatic (A/M) Gastric Cancer (GC)." Journal of Clinical Oncology 34 (15_suppl): 4010–4010. https://doi.org/10.1200/JCO.2016.34.15_suppl.4010.

Janowitz, Tobias, Peter Thuss-Patience, Andrea Marshall, Jung Hun Kang, Claire Connell, Natalie Cook, Janet Dunn, Se Hoon Park, and Hugo Ford. 2016. "Chemotherapy vs Supportive Care Alone for Relapsed Gastric, Gastroesophageal Junction, and Oesophageal Adenocarcinoma: A Meta-Analysis of Patient-Level Data." British Journal of Cancer 114 (4): 381–87. https://doi.org/10.1038/bjc.2015.452.

Kang, Jung Hun, Soon Il Lee, Do Hyoung Lim, Keon-Woo Park, Sung Yong Oh, Hyuk-Chan Kwon, In Gyu Hwang, et al. 2012. "Salvage Chemotherapy for Pretreated Gastric Cancer: A Randomized Phase III Trial Comparing Chemotherapy plus Best Supportive Care with Best Supportive Care Alone." Journal of Clinical

Oncology: Official Journal of the American Society of Clinical Oncology 30 (13): 1513–18. https://doi.org/10.1200/JCO.2011.39.4585.

Kang, Yoon-Koo, Taroh Satoh, Min-Hee Ryu, Yee Chao, Ken Kato, Hyun Cheol Chung, Jen-Shi Chen, et al. 2017. "Nivolumab (ONO-4538/BMS-936558) as Salvage Treatment after Second or Later-Line Chemotherapy for Advanced Gastric or Gastro-Esophageal Junction Cancer (AGC): A Double-Blinded, Randomized, Phase III Trial." Journal of Clinical Oncology 35 (4_suppl): 2–2. https://doi.org/10.1200/JCO.2017.35.4_suppl.2.

Kroemer, Guido, Lorenzo Galluzzi, Oliver Kepp, and Laurence Zitvogel. 2013. "Immunogenic Cell Death in Cancer Therapy." Annual Review of Immunology 31: 51–72. https://doi.org/10.1146/annurev-immunol-032712-100008.

Lieu, C., J. Bendell, J. D. Powderly, M. J. Pishvaian, H. Hochster, S. G. Eckhardt, R. Funke, C. Rossi, D. Waterkamp, and H. Hurwitz. 2014. "1049OSAFETY AND EFFICACY OF MPDL3280A (ANTI-PDL1) IN COMBINATION WITH BEVACIZUMAB (BEV) AND/OR CHEMOTHERAPY (CHEMO) IN PATIENTS (PTS) WITH LOCALLY ADVANCED OR METASTATIC SOLID TUMORS." Annals of Oncology 25 (suppl_4): iv361–iv361. https://doi.org/10.1093/annonc/mdu342.2.

Muro, Kei, Hyun Cheol Chung, Veena Shankaran, Ravit Geva, Daniel Catenacci, Shilpa Gupta, Joseph Paul Eder, et al. 2016. "Pembrolizumab for Patients with PD-L1-Positive Advanced Gastric Cancer (KEYNOTE-012): A Multicentre, Open-Label, Phase 1b Trial." The Lancet. Oncology 17 (6): 717–26. https://doi.org/10.1016/S1470-2045(16)00175-3.

Nanda, Rita, Minetta C. Liu, Christina Yau, Smita Asare, Nola Hylton, Laura Van't Veer, Jane Perlmutter, et al. 2017. "Pembrolizumab plus Standard Neoadjuvant Therapy for High-Risk Breast Cancer (BC): Results from I-SPY 2." Journal of Clinical Oncology 35 (15_suppl): 506–506. https://doi.org/10.1200/JCO.2017.35.15_suppl.506.

Rizzo, J. Douglas, Melissa Brouwers, Patricia Hurley, Jerome Seidenfeld, Murat O. Arcasoy, Jerry L. Spivak, Charles L. Bennett, et al. 2010. "American Society of Clinical Oncology/American Society of Hematology Clinical Practice Guideline Update on the Use of Epoetin and Darbepoetin in Adult Patients with Cancer." Journal of Clinical Oncology: Official Journal of the American Society of Clinical Oncology 28 (33): 4996–5010. https://doi.org/10.1200/JCO.2010.29.2201.

Shitara, Kohei, Kei Muro, Yasuhiro Shimada, Shuichi Hironaka, Naotoshi Sugimoto, Yoshito Komatsu, Tomohiro Nishina, et al. 2016. "Subgroup Analyses of the Safety and Efficacy of Ramucirumab in Japanese and Western Patients in RAINBOW: A Randomized Clinical Trial in Second-Line Treatment of Gastric Cancer." Gastric Cancer: Official Journal of the International Gastric Cancer Association and the Japanese Gastric Cancer Association 19 (3): 927–38. https://doi.org/10.1007/s10120-015-0559-z.

Shitara, Kohei, Mustafa Özgüroğlu, Yung-Jue Bang, Maria Di Bartolomeo, Mario Mandalà, Min-Hee Ryu, Lorenzo Fornaro, et al. 2018. "Pembrolizumab versus Paclitaxel for Previously Treated, Advanced Gastric or Gastro-Oesophageal Junction Cancer (KEYNOTE-061): A Randomised, Open-Label, Controlled,

Phase 3 Trial." Lancet (London, England), June. https://doi.org/10.1016/S0140-6736(18)31257-1.

Simon, R. 1989. "Optimal Two-Stage Designs for Phase II Clinical Trials." Controlled Clinical Trials 10 (1): 1–10.

Smith, Thomas J., James Khatcheressian, Gary H. Lyman, Howard Ozer, James O. Armitage, Lodovico Balducci, Charles L. Bennett, et al. 2006. "2006 Update of Recommendations for the Use of White Blood Cell Growth Factors: An Evidence-Based Clinical Practice Guideline." Journal of Clinical Oncology: Official Journal of the American Society of Clinical Oncology 24 (19): 3187–3205. https://doi.org/10.1200/JCO.2006.06.4451.

Smyth, Elizabeth, and Peter C. Thuss-Patience. 2018. "Immune Checkpoint Inhibition in Gastro-Oesophageal Cancer." Oncology Research and Treatment 41 (5): 272–80. https://doi.org/10.1159/000489099.

Thuss-Patience, Peter C., Albrecht Kretzschmar, Dmitry Bichev, Tillman Deist, Axel Hinke, Kirstin Breithaupt, Yasemin Dogan, Bernhard Gebauer, Guido Schumacher, and Peter Reichardt. 2011. "Survival Advantage for Irinotecan versus Best Supportive Care as Second-Line Chemotherapy in Gastric Cancer--a Randomised Phase III Study of the Arbeitsgemeinschaft Internistische Onkologie (AIO)." European Journal of Cancer (Oxford, England: 1990) 47 (15): 2306–14. https://doi.org/10.1016/j.ejca.2011.06.002.

Thuss-Patience, Peter C., Manish A. Shah, Atsushi Ohtsu, Eric Van Cutsem, Jaffer A. Ajani, Hugo Castro, Wasat Mansoor, et al. 2017. "Trastuzumab Emtansine versus Taxane Use for Previously Treated HER2-Positive Locally Advanced or Metastatic Gastric or Gastro-Oesophageal Junction Adenocarcinoma (GATSBY): An International Randomised, Open-Label, Adaptive, Phase 2/3 Study." The Lancet. Oncology 18 (5): 640–53. https://doi.org/10.1016/S1470-2045(17)30111-0.

Wilke, Hansjochen, Kei Muro, Eric Van Cutsem, Sang-Cheul Oh, György Bodoky, Yasuhiro Shimada, Shuichi Hironaka, et al. 2014. "Ramucirumab plus Paclitaxel versus Placebo plus Paclitaxel in Patients with Previously Treated Advanced Gastric or Gastro-Oesophageal Junction Adenocarcinoma (RAINBOW): A Double-Blind, Randomised Phase 3 Trial." The Lancet. Oncology 15 (11): 1224–35. https://doi.org/10.1016/S1470-2045(14)70420-6.

21.2 ECOG Performance Status

Grade	ECOG performance status
**0**	Fully active, able to carry on all pre-disease performance without restriction
**1**	Restricted in physically strenuous activity but ambulatory and able to carry out work of a light or sedentary nature, e.g., light house work, office work
**2**	Ambulatory and capable of all selfcare but unable to carry out any work activities; up and about more than 50% of waking hours
**3**	Capable of only limited selfcare; confined to bed or chair more than 50% of waking hours
**4**	Completely disabled; cannot carry on any selfcare; totally confined to bed or chair
**5**	death

Table 7 ECOG Performance status

21.3 CTCAE v 5.0

In the present study, adverse events and/or adverse drug reactions will be recorded according to the Common Terminology Criteria for Adverse Events (CTCAE), version 5.0.

At the time this protocol was issued, the full CTC document was available on the NCI web site, at the following address: https://evs.nci.nih.gov/ftp1/CTCAE/CTCAE_5.0/

Another option is via the EORTC Headquarters web site http://www.eortc.org/, which provides a link to the appropriate CTC web site. This link will be updated if the CTC address is changed.

21.4 RECIST v1.1 and modified RECIST

Conventional response criteria may not be adequate to characterize the anti-tumor activity of immunotherapeutic agents like avelumab, which can produce delayed responses that may be preceded by initial apparent radiological progression, including the appearance of new lesions. Therefore, modified response criteria have been developed that account

for the possible appearance of new lesions and allow radiological progression to be confirmed at a subsequent assessment. In this protocol, patients will be permitted to continue study treatment even if the criteria for progressive disease according to RECIST 1.1 are met for the first time if the treating physician decides that the patient clearly benefits from the treatment and the risk/benefit ratio is favorable. This case needs to be discussed with the coordinating investigator in Berlin. If CT confirms progressive disease at least 4 weeks later (confirmed PD) the treatment must be stopped.

Modified RECIST is derived from RECIST, Version 1.1 conventions (Eisenhauer, Therasse et al. 2009) and immune-related response criteria (irRC) (Wolchok, Hoos et al. 2009). When not otherwise specified, RECIST v1.1 conventions will apply.

	RECIST v1.1	Modified RECIST
New lesions after baseline	Define progression.	New measurable lesions are added into the total tumor burden and followed.
Non-target lesions	May contribute to the designation of overall progression.	Contribute only in the assessment of a complete response.
Radiographic progression	First instance of > 20% increase in the sum of diameters or unequivocal progression in non-target disease.	Determined only on the basis of measurable disease; *must* be confirmed by a consecutive assessment > 4 weeks from the date first documented .

RECIST = Response Evaluation Criteria in Solid Tumors.

Table 8 Modified RECIST and RECIST, Version 1.1: Summary of changes

21.4.1 Definitions of measurable/non-measurable lesions

All measurable and non-measurable lesions should be assessed at screening and at the protocol-specified tumor assessment timepoints. Additional assessments may be performed, as clinically indicated for suspicion of progression. The investigator will evaluate response to treatment using RECIST 1.1.

Measurable lesions

Tumor Lesions. Tumor lesions must be accurately measured in at least one dimension (longest diameter in the plane of measurement is to be recorded) with a minimum size as follows:

10 mm by computed tomography (CT) or magnetic resonance imaging (MRI) scan (CT/MRI scan slice thickness/interval no greater than 5 mm)

10-mm caliper measurement by clinical examination (lesions that cannot be accurately measured with calipers should be recorded as non-measurable).

Malignant Lymph Nodes. To be considered pathologically enlarged and measurable, a lymph node must be >15 mm in the short axis when assessed by CT scan (CT scan slice thickness recommended to be no greater than 5 mm). At baseline and follow-up, only the short axis will be measured and followed.

Non-measurable lesions

Non-measurable tumor lesions encompass small lesions (longest diameter <10mm or pathological lymph nodes with short axis ≥10 but <15 mm), as well as truly non-measurable lesions. Lesions considered truly non-measurable include leptomeningeal disease, ascites, pleural or pericardial effusion, inflammatory breast disease, lymphangitic involvement of skin or lung, peritoneal spread, and abdominal mass/abdominal organomegaly identified by physical examination that is not measurable by reproducible imaging techniques.

Special considerations regarding lesion measurability

Bone lesions, cystic lesions, and lesions previously treated with local therapy require particular comment, as outlined below.

Bone Lesions. Bone scan, positron emission tomography (PET) scan, or plain films are not considered adequate imaging techniques for measuring bone lesions. However, these techniques can be used to confirm the presence or disappearance of bone lesions. Lytic bone lesions or mixed lytic–blastic lesions, with identifiable soft tissue components, that can be evaluated by cross-sectional imaging techniques such as CT or MRI can be considered as measurable lesions if the soft tissue component meets the definition of measurability described above. Blastic bone lesions are non-measurable.

Cystic Lesion. Lesions that meet the criteria for radiographically defined simple cysts should not be considered as malignant lesions (neither measurable nor non-measurable) since they are, by definition, simple cysts. Cystic lesions thought to represent cystic metastases can be considered as measurable lesions, if they meet the definition of measurability described above. However, if non-cystic lesions are present in the same patient, these are preferred for selection as target lesions.

21.4.2 Tumor response evaluation

Definitions of target/non-target lesions

Target lesions. When more than one measurable lesion is present at baseline, all lesions up to a maximum of five lesions total (and a maximum of two lesions per organ) representative of all involved organs should be identified as target lesions and will be recorded and measured at baseline. This means that, for instances in which patients have only one or two organ sites involved, a maximum of two lesions (one site) and four lesions (two sites), respectively, will be recorded. Other lesions (albeit measurable) in those organs will be recorded as non-measurable lesions (even if the size is >10 mm by CT scan).

Target lesions should be selected on the basis of their size (lesions with the longest diameter) and be representative of all involved organs, but in addition, should lend themselves to reproducible repeated measurements. It may be the case that, on occasion, the largest lesion does not lend itself to reproducible measurement, in which circumstance, the next largest lesion that can be measured reproducibly should be selected.

Lymph nodes merit special mention since they are normal anatomical structures that may be visible by imaging even if not involved by tumor. As noted above, pathological nodes that are defined as measurable and may be identified as target lesions must meet the criterion of a short axis of >15 mm by CT scan. Only the short axis of these nodes will contribute to the baseline sum. The short axis of the node is the diameter normally used by radiologists to judge if a node is involved by solid tumor. Nodal size is normally reported as two dimensions in the plane in which the image is obtained (for CT, this is almost always the axial plane; for MRI, the plane of acquisition may be axial, sagittal, or coronal). The smaller of these measures is the short axis. For example, an abdominal node that is reported as being 20 mm x 30 mm has a short axis of 20 mm and qualifies as a malignant, measurable node. In this example, 20 mm should be recorded as the node measurement. All other pathological nodes (those with short axis ≥10 mm but <15 mm) should be considered non-target lesions. Nodes that have a short axis of <10 mm are considered non-pathological and should not be recorded or followed. Lesions irradiated within 3 weeks prior to Cycle 1, Day 1 may not be counted as target lesions.

Non-target lesions. All other lesions (or sites of disease), including pathological lymph nodes, should be identified as non-target lesions and should also be recorded at baseline. Measurements are not required.

It is possible to record multiple non-target lesions involving the same organ as a single item on the Case Report Form (CRF) (e.g., “multiple enlarged pelvic lymph nodes” or “multiple liver metastases”).

After baseline, changes in non-target lesions will contribute only in the assessment of complete response (i.e., a complete response is attained only with the complete disappearance of all tumor lesions, including non-target lesions) and will not be used to assess progressive disease.

New lesions. During the study, all new lesions identified and recorded after baseline must be assessed at all tumor assessment timepoints. New lesions will also be evaluated for measurability with use of the same criteria applied to prospective target lesions at baseline per RECIST, (e.g., non-lymph node lesions must be <10 mm; see note for new lymph node lesions below). Up to a maximum of five new lesions total (and a maximum of two lesions per organ), all with measurements at all timepoints, can be included in the tumor response evaluation. New lesion types that would not qualify as target lesions per RECIST cannot be included in the tumor response evaluation.

New lesions that are not measurable at first appearance but meet measurability criteria at a subsequent timepoint will be measured from that point on and contribute to the sum of longest diameters (SLD), if the maximum number of 5 measurable new lesions being followed has not been reached.

Calculation of sum of the diameters

A sum of the diameters (longest for non-nodal lesions, short axis for nodal lesions) for all target lesions will be calculated as a measure of tumor burden.

The sum of the diameters is calculated at baseline and at each tumor assessment for the purpose of classification of tumor responses.

Sum of the diameters at baseline: The sum of the diameters for all target lesions identified at baseline prior to treatment on day 1.

Sum of the diameters at tumor assessment: For every on-study tumor assessment collected per protocol or as clinically indicated, the sum of the diameters at tumor assessment will be calculated using tumor imaging scans. All target lesions and all new measurable

lesions that have emerged after baseline will contribute to the sum of the diameters at tumor assessment. Hence, each net percentage change in tumor burden per assessment with use of modified RECIST accounts for the size and growth kinetics of both old and new lesions as they appear.

Note: In the case of new lymph nodes, RECIST v1.1 criteria for measurability (equivalent to baseline target lesion selection) will be followed. That is, if at first appearance the short axis of a new lymph node lesion >15 mm, it will be considered a measureable new lesion and will be tracked and included in the SLD. Thereafter, the lymph node lesion will be measured at subsequent timepoints and measurements will be included in the SLD, even if the short axis diameter decreases to <15 mm (or even <10 mm). However, if it subsequently decreases to <10 mm, and all other lesions are no longer detectable (or have also decreased to a short axis diameter of <10 mm if lymph nodes), then a response assessment of CR may be assigned.

If at first appearance the short axis of a new lymph node is >10 mm and <15 mm, the lymph node will not be considered measurable but will still be considered a new lesion. It will not be included in the SLD unless it subsequently becomes measurable (short axis diameter >15 mm).

The appearance of new lymph nodes with diameter <10 mm should not be considered pathological and not considered a new lesion.

Response criteria

Evaluation of Target Lesions

Complete Response (CR): Disappearance of all target lesions. Lymph nodes that shrink to □10 mm short axis are considered normal.

Partial Response (PR): At least a 30% decrease in the sum of the diameters of all target and all new measurable lesions, taking as reference the baseline sum of diameters, in the absence of CR.

Note: The appearance of new measurable lesions is factored into the overall tumor burden but does not automatically qualify as progressive disease until the sum of the diameters increases by > 20% when compared with the sum of the diameters at nadir.

Stable Disease (SD): Neither sufficient shrinkage to qualify for PR nor sufficient increase to qualify for PD, taking as reference the smallest sum of the diameters while on study.

Progressive Disease (PD): At least a 20% increase in the sum of diameters of all target and all new measurable lesions, taking as reference the smallest sum on study (nadir SlD;

this includes the baseline sum if that is the smallest on study). In addition to the relative increase of 20%, the sum must also demonstrate an absolute increase of at least 5 mm.

Impact of New Lesions on Modified RECIST

New lesions alone do not qualify as progressive disease according to modified RECIST. However, their contribution to total tumor burden is included in the sum of the diameters, which is used to determine the overall modified RECIST tumor response.

Evaluation of best overall response using modified RECIST

TimePoint Response

It is assumed that at each protocol-specified timepoint, a response assessment occurs. Table provides a summary of the overall response status calculation at each timepoint for patients who have measurable disease at baseline.

Missing assessments and „not evaluable"designation

When no imaging/measurement is done at all at a particular timepoint, the patient is not evaluable (NE) at that timepoint. If only a subset of lesion measurements are made at an assessment, usually the case is also considered NE at that timepoint, unless a convincing argument can be made that the contribution of the individual missing lesion(s) would not change the assigned time point response. This would be most likely to happen in the case of PD. For example, if a patient had a baseline sum of 50 mm with three measured lesions and at follow-up only two lesions were assessed but those gave a sum of 80 mm, the patient will have achieved PD status, regardless of the contribution of the missing lesion.

% Change in sum of the diameters (including measurable new lesions when present)	Target lesion definition	Non-target lesion definition	New measurable lesions	New unmeasurable lesions	Overall modified RECIST timepoint response
- 100% a	CR	CR	No	No	CR
- 100% a	CR	Non-CR or not all evaluated	No	No	PR
≤ - 30%	PR	Any	Yes or no	Yes or no	PR
> - 30% to < + 20%	SD	Any	Yes or no	Yes or no	SD
Not all evaluated	Not evaluated	Any	Yes or no	Yes or no	NE
≥ + 20%	PD	Any	Yes or no	Yes or no	PD

CR = complete response; NE = not evaluable; PD = progressive disease; PR = partial response; RECIST = Response Evaluation Criteria in Solid Tumors; SD = stable disease.

[a] When lymph nodes are included as target lesions, the % change in the sum of the diameters may not be 100% even if complete response criteria are met since a normal lymph node is defined as having a short axis of < 10 mm. Any pathological lymph nodes (whether target or non-target) must have reduction in short axis to < 10 mm in order to meet the definition of CR.

Table 9 Modified RECIST Timepoint Response Definition

Best overall response using modified RECIST: all timepoints

The best overall response is determined once all the data for the patient are known.

The best overall response according to modified RECIST is interpreted as below:

CR: Complete disappearance of all tumor lesions (target and non-target) and no new measurable or unmeasurable lesions, confirmed by a consecutive assessment 4 weeks from the date first documented. All lymph nodes short axes must be < 10 mm.

PR: Decrease in the sum of the diameters of all target and all new measurable lesions ≥ 30% relative to baseline, in the absence of CR, confirmed by a consecutive assessment > 4 weeks from the date first documented.

SD: Criteria for CR, PR, and PD are not met.

PD: Increase in the sum of the diameters of all target and all new measurable lesions ≥ 20% relative to the nadir, which must be confirmed by a consecutive assessment > 4 weeks from the date first documented as follows: The confirmatory assessment shows an additional measurable increase in tumor burden as measured by the sum of the diameters of all target and all new measurable lesions.

Statistical analysis plan (as included in the protocol)

13 DATA ANALYSIS AND STATISTICAL CONSIDERATIONS

13.1 General design and sample size estimation

The study is planned as single arm phase II study.
The primary endpoint of the study is the overall survival rate at 6 months based on the ITT population.
The experimental therapy would be considered to be a highly promising candidate for further development (e.g. in a phase III trial), if the true OS rate amounted to 65% or more.
Paclitaxel/ramucirumab could achieve an OS rate at 6 months of 65% in the Western population of the Rainbow trial, which set paclitaxel/ramucirumab as a standard treatment (Shitara et al. 2016). The Western patient population included in the Rainbow trial had better prognostic parameters than we expect for our study population (no pretreatment with taxanes, we expect 30% of patients being pretreated with a taxane either perioperatively of during 1st-line (Al-Batran et al. 2017). Only 30% of GE-junction tumors in the Rainbow population which have a slightly worse prognosis than tumor locations in the gastric body (Dikken et al. 2013), we expect 50% of GE-junction tumors in our study population). Although we expect worse prognostic parameters in our study population, we hope to achieve an OS rate of 65% at 6 months by combining paclitaxel / ramucirumab with avelumab.
On the other hand, the experimental therapy would be rated as insufficiently active, if the true OS rate is 50% or lower, as this suggests a distinct inferiority to paclitaxel / ramucirumab.
An overall survival rate of 45% at 6 months was detected in the metaanalysis pooling data from the three randomised trials comparing chemotherapy with best supportive care (Janowitz et al. 2016). In 410 pts chemotherapy significantly reduced the risk of death (hazard ratio (HR)=0.63, 95% confidence interval (CI)=0.51-0.77, P<0.0001) with a 6 months survival rate in the chemotherapy arm of 45%. Administering a triple combination of paclitaxel/ramucirumab + avelumab, we need to be clearly better than a monotherapy, therefore an OS rate at 6 months of less than 50% is considered as insufficient.
The following error levels are defined:

- Probability to accept the experimental therapy as promising (≥ 65% OS rate) with respect to efficacy, in spite of a true OS rate of ≤ 50%: 0.10 (type I error)
- Probability to reject the experimental therapy as not sufficiently efficient (≤ 50%), although the true OS rate is promising (≥ 65%): 0.2 (type II error, corresponding to a power of 80%).

According to these parameters, and allowing for the option of earlier stopping for futility in case of unfavorable results, a standard two-stage phase II design according to Simon (Simon 1989) is applied. In the first stage, n = 33 patients with the endpoint available are analyzed, and the trial is stopped if the number of "successes" is only 16 or lower. Otherwise, the study is continued until a total of 53 patients evaluable for efficacy as defined in section 13.2 have been recruited. The final conclusion of the phase II trial will depend on the definite OS rate (and its confidence interval), as well as the information on

type, frequency and severity of toxicities. Formally, more than 31 patients alive at 6 months are required to reject the null hypothesis defined above. Assuming a 10% drop out rate, and in order to achieve adequate power in the per-protocol analysis (see below), we are planning to include 59 pts.

13.2 Analysis populations

The intention-to-treat population is defined as patients who received at least one dose of all three components of treatment (avelumab, ramucirumab and paclitaxel). The IIT population is evaluable for toxicity and as well for efficacy (intention-to-treat, ITT). The safety population consists of all patients who received at least 1 dose of any treatment. In addition, a per-protocol population is defined, consisting of all patients having received at least two full treatment cycles according to the protocol and with available survival status at 6 months after first dose of treatment.

13.3 Statistical methods

The primary endpoint is calculated by deviding the number of patients alive a 6 months by the total number of patients in the ITT population (ITT as defined in section 13.2). Exact 80%, 90% and 95% confidence intervals (two-sided) will be provided for this proportion. Formally, the lower boundary of the 80% confidence interval corresponds to the sample size estimation approach described above.

All other efficacy and toxicity parameters will be evaluated in an explorative or descriptive manner, providing proportions, means, medians, ranges, standard deviations and/or confidence intervals, or Kaplan-Meier estimates, as appropriate.
If p values are calculated (e.g. for comparison of subgroups), they will be presented explicitly without referring to hypotheses or a significance level. Usually, no error adjustment for multiple testing will be performed. Thus the p values will reflect the comparison-wise error and not the experiment-wise error. All p values will be two-sided if not stated otherwise.
Comparisons of the categorical data, e.g. response rates, will be performed using chi² test, Fisher's exact test or a trend test according to Cochran/Armitage, as appropriate.
Secondary endpoints of time-to-event type, i.e. progression-free survival and overall survival, will be estimated according to Kaplan-Meier, and exploratively compared (between subgroups, or to historical data) using the logrank test. Hazard ratios (with confidence intervals) may be derived from corresponding Cox proportional hazard models.
Further details will be prospectively defined in a statistical analysis plan to be written before any efficacy data are analyzed.

Post Hoc Multivariate Analysis:

Univariable and multivariable prognostic analyses were performed using Cox models. Parameters with a univariable $p < 0.1$ were included in the multivariable model, to be reduced by a stepwise backwards procedure at a threshold of $p < 0.1$. SPSS and statistical analyses were performed in "R".
This analysis included age, ECOG, Lauren classification, localization of primary tumor, pretreatment with taxanes, post discontinuation therapy, PD-L1 CPS, cfDNA at baseline and peripheral blood T cell receptor repertoire richness (TRB richness).
