# Supplementary material for: Ramucirumab, Avelumab, and Paclitaxel as Second-Line Treatment in Esophagogastric Adenocarcinoma: The Phase 2 RAP (AIO-STO-0218) Nonrandomized Controlled Trial
Source: JAMA Netw Open. 2024 Jan 23;7(1):e2352830. doi: 10.1001/jamanetworkopen.2023.52830 (PMC10807255; doi:10.1001/jamanetworkopen.2023.52830)
Supplement: Supplement 3. — Data Sharing Statement [file jamanetwopen-e2352830-s003.pdf]

Data Sharing Statement

Thuss-Patience. Ramucirumab, Avelumab, and Paclitaxel as Second-Line Treatment in Esophagogastric Adenocarcinoma: The Phase 2 RAP (AIO-STO-0218) Nonrandomized Controlled Trial. *JAMA Netw Open*. Published online January 23, 2024. doi:10.1001/jamanetworkopen.2023.52830

Data
Data available: No

Additional Information
Explanation for why data not available: all relevant data are included in the manuscript
